# Cellular Mechanisms Underlying Behavioral State-Dependent Bidirectional Modulation of Motor Cortex Output

**DOI:** 10.1016/j.celrep.2015.04.042

**Published:** 2015-05-14

**Authors:** Julia Schiemann, Paolo Puggioni, Joshua Dacre, Miha Pelko, Aleksander Domanski, Mark C.W. van Rossum, Ian Duguid

**Affiliations:** 1 Centre for Integrative Physiology and Patrick Wild Centre, University of Edinburgh, Hugh Robson Building, George Square, Edinburgh EH8 9XD, UK; 2 Institute for Adaptive and Neural Computation, School of Informatics, University of Edinburgh, Edinburgh EH8 9AB, UK; 3 Neuroinformatics Doctoral Training Centre, School of Informatics, University of Edinburgh, Edinburgh EH8 9AB, UK

## Abstract

Neuronal activity in primary motor cortex (M1) correlates with behavioral state, but the cellular mechanisms underpinning behavioral state-dependent modulation of M1 output remain largely unresolved. Here, we performed in vivo patch-clamp recordings from layer 5B (L5B) pyramidal neurons in awake mice during quiet wakefulness and self-paced, voluntary movement. We show that L5B output neurons display bidirectional (i.e., enhanced or suppressed) firing rate changes during movement, mediated via two opposing subthreshold mechanisms: (1) a global decrease in membrane potential variability that reduced L5B firing rates (L5B_suppressed_ neurons), and (2) a coincident noradrenaline-mediated increase in excitatory drive to a subpopulation of L5B neurons (L5B_enhanced_ neurons) that elevated firing rates. Blocking noradrenergic receptors in forelimb M1 abolished the bidirectional modulation of M1 output during movement and selectively impaired contralateral forelimb motor coordination. Together, our results provide a mechanism for how noradrenergic neuromodulation and network-driven input changes bidirectionally modulate M1 output during motor behavior.

## Highlights

• During movement, reduced V_m_ variance lowers spike probability in L5B output neurons
• Noradrenaline mediates a tonic depolarization in L5B_enh_ neurons during movement
• Noradrenaline selectively enhances signal-to-baseline ratio of L5B_enh_ neurons
• Blocking noradrenaline receptors in M1 reduces contralateral forepaw motor coordination

## Graphical Abstract

**Figure:**
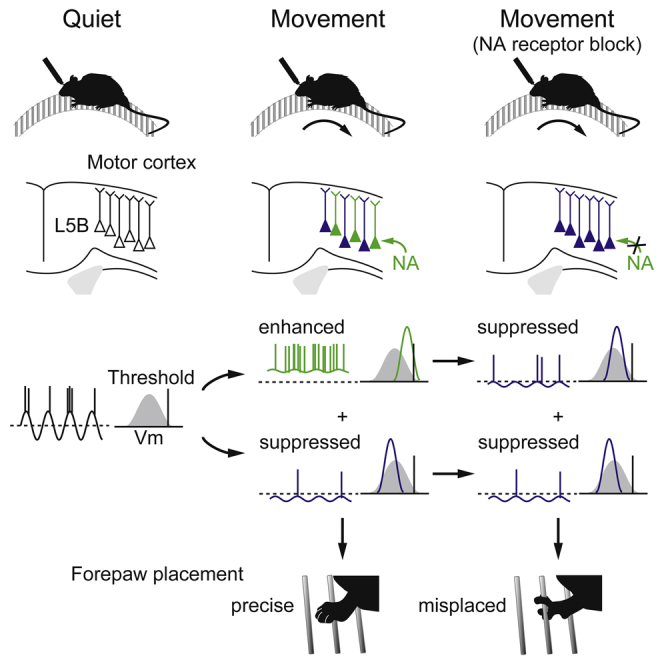

## Teaser

Schiemann et al. show that, in mouse motor cortex, layer 5B pyramidal neuron firing rates are suppressed or enhanced during movement due to a global reduction in membrane potential variability and coincident noradrenaline-mediated depolarization in a subpopulation of neurons, respectively. Blocking noradrenergic input to M1 impairs motor coordination.

## Introduction

Neuronal activity in layer 5 (L5) of primary motor cortex (M1) correlates with rhythmic voluntary movements (Armstrong and Drew, 1984a, 1984b). During walking or running, pyramidal neurons display changes in firing rate that reflect periods of coordinated muscle activity (Armstrong and Drew, 1984a; Beloozerova et al., 2003). Although spontaneous locomotor activity can be controlled by central pattern generators (CPGs) in the spinal cord (Forssberg et al., 1980; Grillner, 1981; Grillner and Zangger, 1979), descending motor commands from M1 are integrated with ongoing rhythmic spinal cord signals and sensory input from the periphery to initiate, adjust, and maintain locomotor function (Armstrong and Drew, 1984a; Beloozerova et al., 2003; Orlovsky, 1972; Ueno and Yamashita, 2011). In lower mammals, such as cats, rabbits, and mice, discrete subpopulations of L5 output neurons display enhanced or suppressed (i.e., bidirectional) firing rate changes during locomotion (Armstrong and Drew, 1984a; Beloozerova et al., 2003; Costa et al., 2004). In rodents, these changes can be either abrupt, sustained changes—so-called on-off responses—or gradual frequency changes linked to the velocity of running (Costa et al., 2004). Although we are now beginning to understand how patterns of motor cortex activity relate to changes in behavioral state in rodents (i.e., quiet wakefulness to movement), the cellular mechanisms underpinning bidirectional modulation of M1 output during self-paced movement remain largely unresolved.

Several mechanisms could underlie the bidirectional modulation of M1 output, such as a change in cortical state-dependent network-driven input structure, intracortical or long-range excitatory input, and/or neuromodulation. During quiet wakefulness or slow-wave sleep, cortical networks remain in a synchronized state that consists of slow, large-amplitude oscillations in neuronal population activity (Cowan and Wilson, 1994; Steriade et al., 1993c). During active behavior, cortical networks enter an activated state characterized by a reduction in slow oscillations and, in some cases, an increase in higher frequency activity (Steriade et al., 1993b; Timofeev et al., 2001). This change profoundly alters the subthreshold V_m_ dynamics and spike output patterns of cortical pyramidal neurons (Castro-Alamancos, 2004; Castro-Alamancos and Oldford, 2002; Constantinople and Bruno, 2011; Crochet and Petersen, 2006). Thalamic activation promotes the cortical awake state and direct depolarization of superficial and deep-layer pyramidal neurons (Castro-Alamancos and Connors, 1996; Castro-Alamancos and Oldford, 2002; Constantinople and Bruno, 2013; Hirata and Castro-Alamancos, 2010; Poulet et al., 2012), suggesting input from the thalamus could contribute to changes in M1 output during motor behavior. Anatomical and functional connectivity mapping have shown the presence of a strong top-down laminar organization of local excitatory microcircuits in M1, with feedforward projections from layer 2/3 (L2/3) targeting multiple classes of projection neurons in L5 (Kaneko et al., 1994; Weiler et al., 2008). Given that L2/3 pyramidal neurons can display dense clustered activity during head-restrained locomotion in mice (Dombeck et al., 2009), changes in descending excitation from upper-layer pyramidal neurons could be a mechanism for generating bidirectional modulation of M1 output. Alternatively, neuromodulators are important for cortical processing, with noradrenaline and acetylcholine release being associated with changes in arousal, vigilance, and behavioral state (Berridge and Waterhouse, 2003; Carter et al., 2010; Castro-Alamancos and Gulati, 2014; Constantinople and Bruno, 2011; Eggermann et al., 2014; Fu et al., 2014; Polack et al., 2013; Steriade et al., 1993a). Thus, how local, long-range, and neuromodulatory inputs regulate L5 pyramidal neuron V_m_ dynamics during changes in behavioral state remains to be fully established.

Here we combined in vivo patch-clamp recordings in awake mice with selective pharmacology to investigate the cellular mechanisms underpinning behavioral state-dependent modulation of motor cortex output. We found that changing behavioral state, from quiet wakefulness to movement, bidirectionally modulated (i.e., enhanced or suppressed) M1 output via two opposing subthreshold mechanisms: (1) a global decrease in network-driven, slow, large-amplitude V_m_ fluctuations, which reduced V_m_ variability, spike probability, and firing rates in L5B pyramidal neurons (L5B_suppressed_ neurons); and (2) a coincident increase in excitatory drive to a subpopulation of L5B neurons (L5B_enhanced_), which depolarized mean V_m_ and enhanced firing rates. We found that the movement-related tonic depolarization in L5B_enh_ neurons was dependent on the interplay between ascending motor thalamic input, which maintained V_m_ near threshold, and noradrenergic input from the locus coeruleus (LC). The behavioral state-dependent release of noradrenaline increased the signal-to-baseline ratio (SBR) for movement-evoked responses in L5B_enh_ neurons. Selectively blocking noradrenergic input in the forelimb region of M1 significantly reduced motor coordination in the contralateral forelimb during motor behavior. Thus, our findings provide a mechanism for how noradrenergic neuromodulation and network-driven input changes bidirectionally modulate M1 output during self-paced voluntary movement.

## Results

### Membrane Potential Dynamics of L5B Pyramidal Neurons during Self-Paced, Voluntary Movement

**Figure 1:**
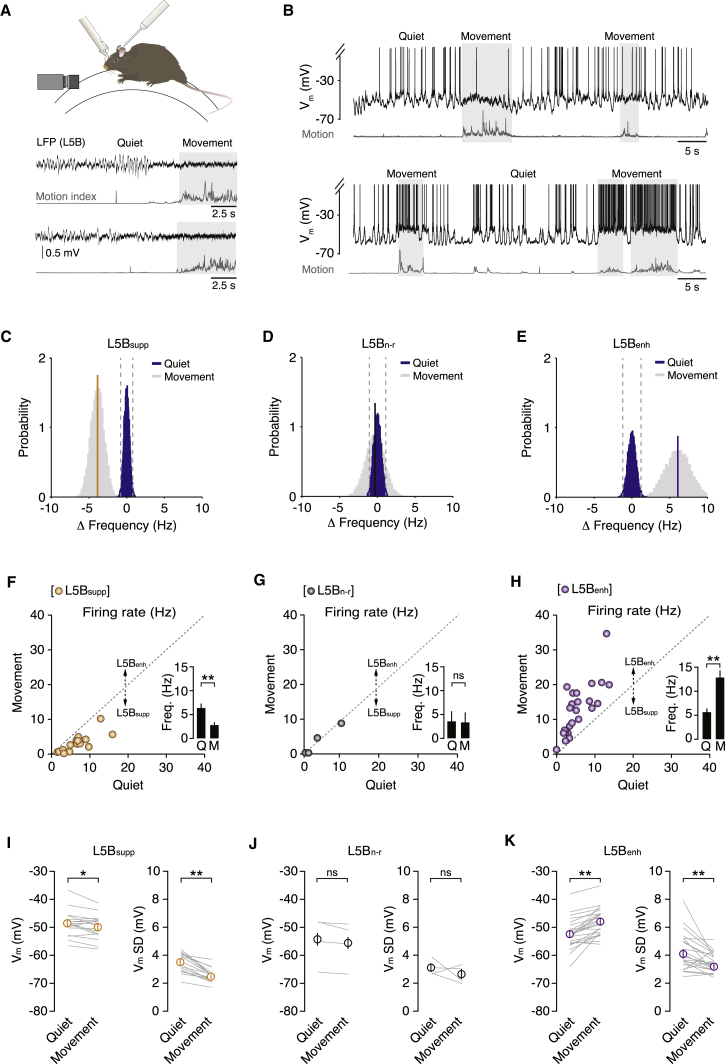
Membrane Potential Dynamics of L5B Pyramidal Neurons in M1 during Self-Paced, Voluntary Movement (A) Patch-clamp recording configuration in head-fixed mice mounted on a single-axis, cylindrical treadmill. Local field potential (LFP) recordings (black traces, n = 2 mice) of L5B activity and moderate speed (60 frames/s) digital imaging were used to confirm changes in behavioral state (quiet wakefulness to movement) and to calculate motion index (gray traces). (B) Representative voltage traces from two L5B pyramidal neurons that displayed either a decrease (top) or increase (bottom) in firing rate during movement (light gray shading). The motion index (dark gray) defines the magnitude and duration of each forelimb movement. In this figure and all subsequent figures, action potentials have been truncated to highlight subthreshold V_m_ changes during movement. (C–E) Representative change in firing rate probability distributions during quiet wakefulness (blue) and movement (gray) in L5B_suppressed_ (C), L5B_non-responding_ (D), and L5B_enhanced_ (E) neurons. Gray dotted lines represent the 1^st^ (left) and 99^th^ (right) percentiles. Solid colored lines represent the average firing rate change in L5B_suppressed_ (yellow), L5B_non-responding_ (black), and L5B_enhanced_ (purple) neurons during movement. (F–H) Average firing rate during quiet wakefulness and movement in L5B_suppressed_ (F, n = 17), L5B_non-responding_ (G, n = 4), and L5B_enhanced_ (H, n = 24) neurons. Filled circles represent data from individual neurons. Insets depict the average firing rate ± SEM during quiet wakefulness (Q) and movement (M). ^∗∗^p < 0.01; ns, non-significant. (I–K) Average V_m_ (left-hand plot) and V_m_ SD (right-hand plot) in L5B_suppressed_ (I, n = 17), L5B_non-responding_ (J, n = 4), and L5B_enhanced_ (K, n = 24) neurons during quiet wakefulness and movement. Solid gray lines represent data from individual neurons while open symbols represent mean ± SEM. ^∗^p < 0.05, ^∗∗^p < 0.01; ns, non-significant. See also Figures S1–S4 and Tables S1 and S2.

To investigate the cellular mechanisms underpinning behavioral state-dependent modulation of M1 output, we obtained whole-cell patch-clamp recordings from L5B pyramidal neurons (forelimb motor cortex, 620–880 μm from the pial surface; see Experimental Procedures; n = 45 neurons) during quiet wakefulness and self-paced, voluntary movements (i.e., walking, running, or grooming on a single-axis, cylindrical treadmill; Figure 1A). During periods of quiet wakefulness, all L5B pyramidal neurons displayed large-amplitude V_m_ fluctuations (V_m_ SD = 3.8 ± 0.2 mV) and a relatively depolarized average V_m_ (V_m_ = −51.1 ± 0.8 mV). The interplay among mean V_m_, distance from threshold, and V_m_ variability resulted in moderate basal firing rates (5.7 ± 0.6 Hz, range: 0.0–15.9 Hz; Figures 1B–1K and S1).

During switches in behavioral state (i.e., quiet wakefulness to movement), characterized by a low-amplitude, high-frequency local field potential signal in L5B (Figure 1A), the vast majority of L5B pyramidal neurons (∼90%) displayed significant modulation of their basal firing rates. To functionally classify individual neurons, we compared the variability in quiet wakefulness firing rate with the average firing rate during self-paced movement (see Experimental Procedures). If the average movement-related firing rate was lower than the first percentile of the distribution of firing rate changes during quiet wakefulness, neurons were classified as suppressed (L5B_supp_, n = 17; Figures 1C and 1F; Table S1), while neurons that displayed an average movement-related firing rate above the 99^th^ percentile were classified as enhanced (L5B_enh_, n = 24; Figures 1E and 1H; Table S1). A small proportion of L5B neurons (n = 4/45) did not significantly change their firing rates during movement and were classified as non-responding neurons (L5B_n-r_; Figures 1D and 1G). The proportion of L5 pyramidal neurons in which spike frequency decreased (38%), increased (53%), or did not change (9%) during movement was consistent with previous reports (Beloozerova et al., 2003; Costa et al., 2004). Moreover, the functional classification of individual neurons remained consistent during repeated bouts of movement and was not dependent on the type of motor behavior being executed (Figure S1). To further demonstrate the coexistence of functionally distinct subpopulations of L5B pyramidal neurons in M1, we performed multiple recordings from the same mouse and identified L5B_enh_, L5B_supp_, and L5B_n-r_ pyramidal neurons during the execution of similar forelimb movements (i.e., repeated forepaw swing/stance cycles, n = 8 recordings from three mice; L5B_supp_/L5B_enh_/L5B_n-r_ ratio: 4:3:1, note similar ratio of functionally classified neurons when compared to the population data in Figure 1; Figure S1).

We next investigated the subthreshold mechanisms underpinning bidirectional modulation of M1 output. During movement, L5B_supp_ neurons displayed ∼1 mV hyperpolarization in mean V_m_ (p = 2 × 10^−2^) and reduced V_m_ variability (V_m_ SD quiet = 3.5 ± 0.2 mV, V_m_ SD movement = 2.5 ± 0.1 mV, p = 3 × 10^−4^), which lowered the probability of reaching threshold and reduced overall firing rates (quiet 6.4 ± 1.0 Hz, movement 2.8 ± 0.6 Hz, p = 3 × 10^−4^; Figures 1F and 1I). In L5B_enh_ neurons, movement also reduced V_m_ variability (V_m_ SD quiet = 4.1 ± 0.3 mV, V_m_ SD movement = 3.2 ± 0.2 mV, p = 2 × 10^−3^), but this was counteracted by a depolarization in average V_m_ (quiet −52.4 ± 1.1 mV, movement −47.9 ± 1.0 mV, p = 2 × 10^−6^), which significantly increased spike probability and firing rates (quiet 5.7 ± 0.8 Hz, movement 12.9 ± 1.5 Hz, p = 2 × 10^−5^; Figures 1H and 1K). Moreover, movement-related firing rate changes strongly correlated with the level of V_m_ depolarization in individual L5B_enh_ neurons (Figure S2). By contrast, V_m_ dynamics and firing rates of L5B_n-r_ neurons were not affected by the transition from quiet wakefulness to movement (Figures 1G and 1J). Interestingly, the functional classification of L5B pyramidal neurons (L5B_supp_ versus L5B_enh_) was not dependent on their basic electrophysiological properties (Table S1) or the projection-class identity of individual neurons based on retrograde tracing and selective expression of the transcription factors CTIP2 (thick-tufted pyramidal tract [PT]-type neurons) and SATB2 (thin-tufted intratelencephalic [IT]-type neurons; Leone et al., 2008; Figures S3 and S4; Table S2). Together, our results suggest that movement-related modulation of L5B_enh_ firing rates is primarily mediated by a tonic depolarization in V_m_, while reduced firing rates in L5B_supp_ neurons result from a moderate hyperpolarization and significant reduction in V_m_ variance.

### L5B Input-Output Transformations during Voluntary Movement

**Figure 2:**
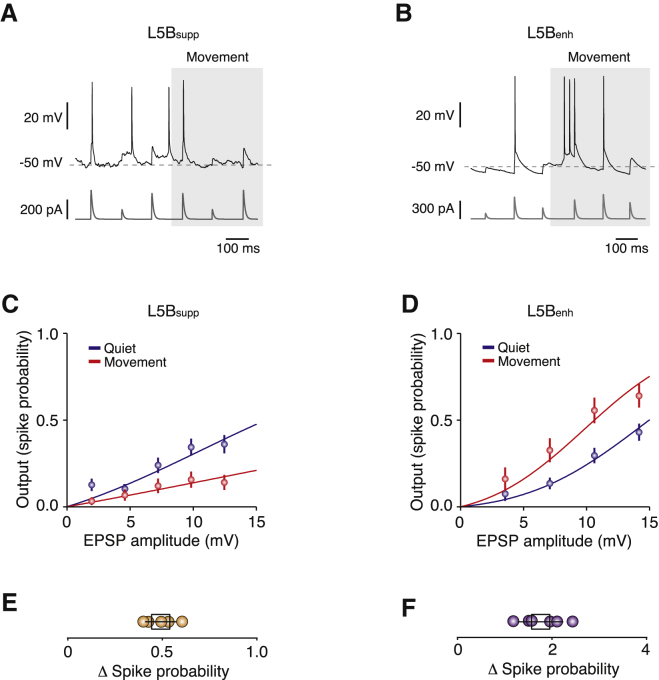
Input-Output Transformations in L5B_supp_ and L5B_enh_ Pyramidal Neurons during Movement (A and B) Representative voltage traces (upper trace, black) during somatic EPSC-like current injections in vivo (lower trace, dark gray) in L5B_supp_ (A) and L5B_enh_ (B) pyramidal neurons during quiet wakefulness and movement (light gray shading). (C and D) Input-output transformations in L5B_supp_ (C, n = 5) and L5B_enh_ (D, n = 6) neurons recorded in vivo during quiet wakefulness (blue) and movement (red). Symbols represent mean ± SD; solid lines are fits to a truncated error function. (E and F) Mean change in spike probability for L5B_supp_ (E, n = 5) and L5B_enh_ (F, n = 6) neurons. Filled symbols represent data from individual neurons and black open squares represent mean ± SD. See also Figure S2.

Behavioral state-dependent changes in V_m_ dynamics can profoundly affect the integrative mode and output firing patterns of neocortical neurons. What effects do movement-related changes in V_m_ dynamics have on input-output transformations in M1 L5B pyramidal neurons? In principle, both changes in V_m_ SD and mean can influence the responsiveness and firing dynamics of a neuron (Chance et al., 2002; Hô and Destexhe, 2000). To test this, we performed current injection experiments (i.e., somatic injection of excitatory postsynaptic current [EPSC]-like waveforms) in a subset of L5B_supp_ and L5B_enh_ neurons in vivo (Figures 2A and 2B; Supplemental Experimental Procedures) and measured the spike probability during quiet wakefulness and voluntary movement. Although current injection at the soma disregards dendritic non-linearities, synaptic properties, and locations, it provides a robust measure to assess the relationship between synaptic conductances arriving at the soma and spike output probability during behavior. During movement L5B_supp_ neurons, which experience a decrease in V_m_ SD with relatively little change in mean V_m_ (Figure 1), displayed a 2-fold reduction in spike probability (Δ Spike probability = 0.6 ± 0.1, n = 5; Figures 2C and 2E). By contrast, L5B_enh_ neurons, which experience a decrease in V_m_ SD and an increase in mean V_m_ (Figure 1), displayed a 2-fold increase in spike probability (Δ Spike probability = 1.7 ± 0.4, n = 6; Figures 2D and 2F). Although both L5B subpopulations displayed moderate changes in input resistance during movement, they did not significantly differ from quiet wakefulness (n = 5 and 5, respectively, p = 0.32; Figure S2).

### Changes in L5B Input Structure during Movement

**Figure 3:**
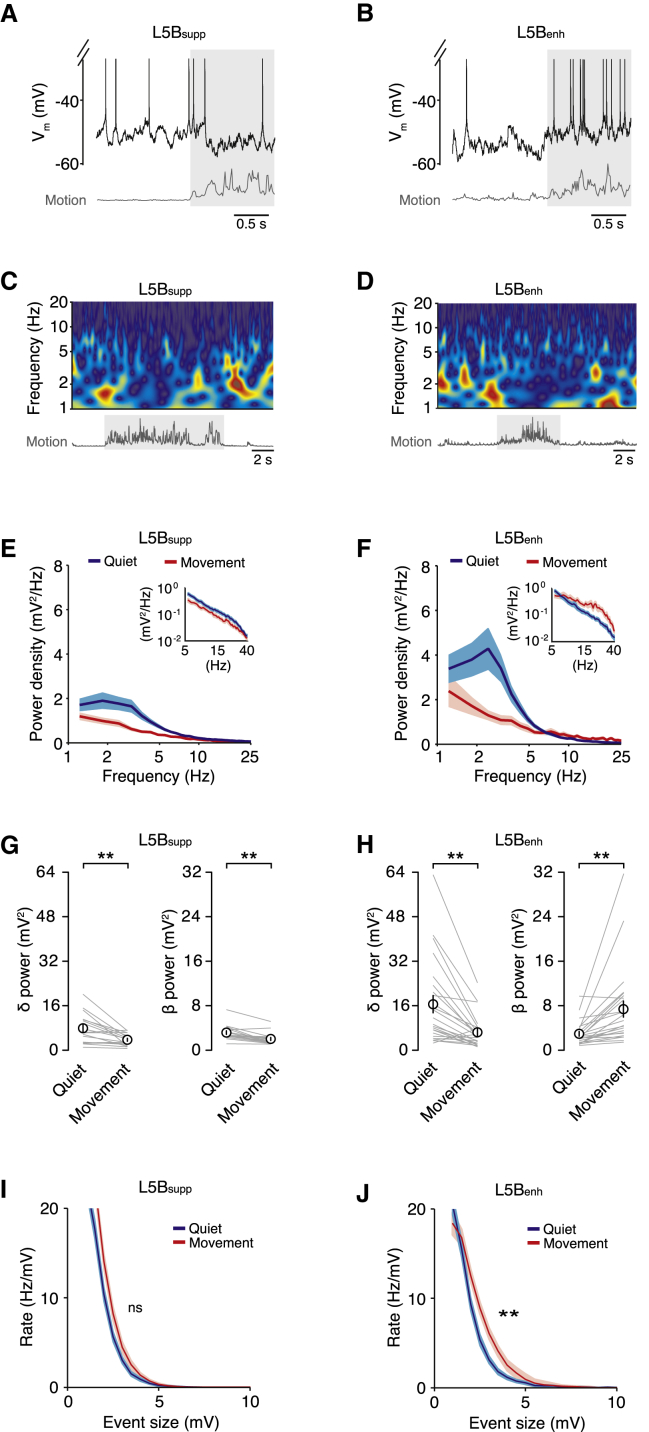
Movement Reduces Slow, Large-Amplitude V_m_ Fluctuations but Increases Excitatory Drive in L5B_enh_ Neurons (A and B) Representative high-time resolution voltage traces for L5B_supp_ (A) and L5B_enh_ (B) neurons during quiet wakefulness and movement (gray shading). (C and D) Low-time resolution wavelet spectrograms for L5B_supp_ (C) and L5B_enh_ (D) neurons during quiet wakefulness and movement. Representative examples correspond to neurons shown in (A and B). (E and F) Average V_m_ power density for L5B_supp_ (E, n = 17) and L5B_enh_ (F, n = 24) pyramidal neurons during quiet wakefulness (blue) and movement (red). Data represent mean ± SD. Insets show average V_m_ power density between 5 and 40 Hz. (G and H) Average V_m_ power in δ (1.5–4 Hz) and β (12–30 Hz) frequency bands in L5B_supp_ (G, n = 17) and L5B_enh_ (H, n = 24) pyramidal neurons during quiet wakefulness and movement. Gray lines represent data from individual neurons and black symbols represent mean ± SEM. ^∗∗^p < 0.01. (I and J) Average rate density of compound synaptic events in L5B_supp_ (I, n = 17) and L5B_enh_ (J, n = 24) pyramidal neurons during quiet wakefulness (blue) and movement (red). Data represent mean ± SD. ^∗∗^p < 0.01; ns, non-significant. See also Figure S2.

To further investigate the mechanisms underpinning L5B_supp_ and L5B_enh_ neuron V_m_ dynamics, we explored changes in V_m_ spectral components before and after movement onset. During quiet wakefulness, we observed slow (1.5–4 Hz, δ frequency band), large-amplitude V_m_ fluctuations in all L5B pyramidal neurons (Figures 3A–3D), which were suppressed during movement (L5B_supp_ quiet 7.8 ± 1.3 mV^2^, movement 3.6 ± 0.5 mV^2^, n = 17, p = 2 × 10^−3^; L5B_enh_ quiet 16.4 ± 3.1 mV^2^, movement 6.2 ± 1.2 mV^2^, n = 24, p = 1 × 10^−4^; Figures 3A–3H). The reduction in δ power led to reduced V_m_ SD, which together with a moderate hyperpolarization (∼1 mV) could account for the reduction in spike probability observed in L5B_supp_ pyramidal neurons during movement (Figures 1 and S2). In L5B_enh_ neurons, the suppression of slow V_m_ fluctuations was counteracted by an increase in power (12–30 Hz) in the β frequency band (12–30 Hz; quiet 3.0 ± 0.4 mV^2^, movement 7.4 ± 1.4 mV^2^, n = 24, p = 3 × 10^−5^; Figures 3F and 3H). The magnitude of increased β power displayed a strong positive correlation with the magnitude of V_m_ depolarization in individual L5B_enh_ neurons (Figure S2), suggesting this could be the source of the increased excitatory drive.

To examine this further, we developed an event detection algorithm to estimate the level of excitatory input during quiet wakefulness and movement. Due to the high frequency of afferent input (estimated range: 5–15 kHz, data not shown), we were unable to isolate single excitatory postsynaptic potentials (EPSPs). However, we could reliably detect compound synaptic inputs (≥1 mV) occurring in a time window (5 ms) shorter than the average membrane time constant (8.2 ± 0.7 ms, n = 10; Figure S2). The detection threshold corresponded to twice the size of the average unitary synaptic response measured in L5 pyramidal neurons in vitro (Deuchars et al., 1994; Reyes and Sakmann, 1999). Events that occurred within ±10 ms of a spike were excluded from the analysis. During quiet wakefulness, we detected fast-rising compound EPSPs (range: 1–9.7 mV) with similar rates in both L5B_supp_ and L5B_enh_ pyramidal neurons (Figures 3I and 3J), indicating both subpopulations of neurons receive a comparable level of excitatory drive. During movement, the rate of compound events in L5B_supp_ neurons was not affected (n = 17; Figure 3I), whereas L5B_enh_ neurons displayed a significant increase in compound EPSP rate (n = 24; Figure 3J). Remarkably, we did not detect any compound events with amplitudes greater than 9.4 mV, even though neurons spent approximately 50% of the time >10 mV from threshold. Thus, L5B_enh_ neurons appear to preferentially receive a net increase in excitatory drive during movement, which enhances the firing rate by depolarizing mean V_m_ and increasing spike probability.

### Effects of Local and Long-Range Input to L5B Pyramidal Neurons during Self-Paced Movement

**Figure 4:**
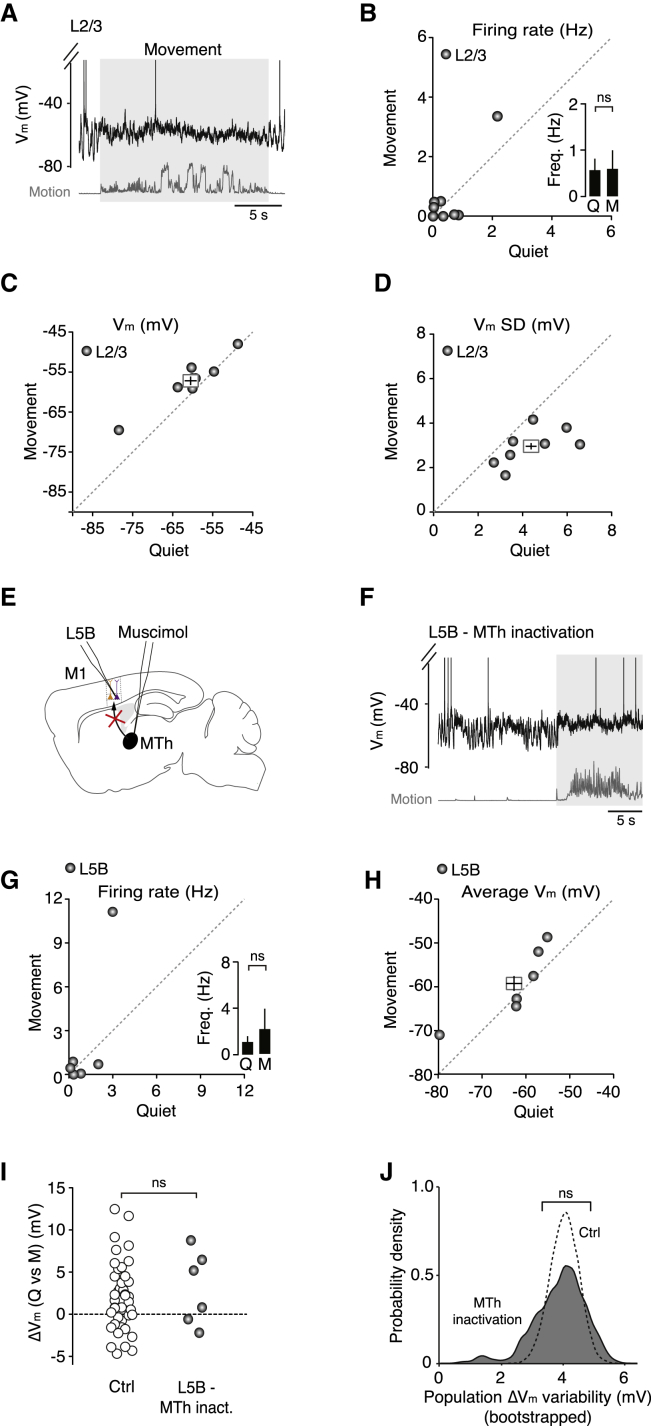
Effect of Descending L2/3 and Ascending Motor Thalamic Input on L5B Pyramidal Neuron V_m_ Dynamics during Quiet Wakefulness and Movement (A) Representative voltage trace shows an L2/3 pyramidal neuron during quiet wakefulness and movement (gray shading). (B–D) Average firing rate (B), mean V_m_ (C), and V_m_ SD (D) in L2/3 pyramidal neurons (gray symbols, n = 8) before and after movement. Filled circles represent data from individual neurons while square symbols represent mean ± SEM. Inset in (B) depicts average L2/3 pyramidal neuron firing rate during quiet wakefulness (Q) and movement (M). ns, non-significant. (E) Schematic representation shows an L5B pyramidal neuron recording after inactivation of ipsilateral motor thalamus (MTh) by local perfusion of muscimol. (F) Representative voltage trace showing an L5B pyramidal neuron after ipsilateral inactivation of motor thalamus. (G and H) Average firing rate (G) and mean V_m_ (H) in L5B pyramidal neurons after motor thalamic inactivation (n = 6). Filled circles represent data from individual neurons while the square symbol in (H) represents the mean ± SEM. Inset in (G) depicts the average firing rate of L5B neurons during quiet wakefulness (Q) and movement (M). ns, non-significant. (I) Change in average V_m_ (ΔV_m_) during movement in the presence (Ctrl, open symbols, n = 41) and absence of motor thalamic input (gray symbols, n = 6). Control data (Ctrl) were taken from Figure 1 for comparison. Mann-Whitney U test; ns, non-significant. (J) Probability density distributions of ΔV_m_ variability across the L5B pyramidal neuron population (Ctrl and MTh inact.), measured as the SD of the ΔV_m_ distributions shown in (I) (Population ΔV_m_ SD) using bootstrap analysis (10,000 bootstrap replicates). Black dashed line represents population ΔV_m_ variability distribution in control (Ctrl), and gray shading represents population ΔV_m_ variability distribution following motor thalamic inactivation. Control data (Ctrl) were taken from Figure 1 for comparison. F test; ns, non-significant. See also Figure S5.

To investigate the possible source(s) of the increased excitatory drive to L5B_enh_ neurons, we examined the activity of local and long-range inputs from L2/3 and motor thalamus, respectively. Previous studies have shown that M1 L2/3 neurons can be highly active during head-restrained locomotion on a spherical treadmill (Dombeck et al., 2009) and this descending excitation could potentially influence the activity of output neurons in L5 (Weiler et al., 2008). To test this possibility, we obtained whole-cell patch-clamp recordings from L2/3 pyramidal neurons (180–420 μm from the pial surface; Figure 4A). During quiet wakefulness, L2/3 neurons displayed relatively low firing rates, which were unaffected by the onset of movement (quiet 0.6 ± 0.3 Hz, movement 0.6 ± 0.4 Hz, n = 8; Figure 4B). Although the average V_m_ of L2/3 neurons depolarized by ∼4 mV (Figure 4C), this was counteracted by a significant reduction in V_m_ SD (Figure 4D), which maintained baseline spike probability and firing rates. Given that our sample of L2/3 neurons displayed low spike rates during both quiet wakefulness and movement, this suggests that descending input from L2/3 is unlikely to be the primary source of the increased excitatory drive to L5B_enh_ neurons in our experimental paradigm (see also Dombeck et al., 2009 and Discussion).

Given that thalamocortical neuron firing rates vary markedly depending on behavioral state and ventroanterior/ventrolateral (VA/VL) thalamic input to M1 displays bidirectional modulation during simple locomotion (Marlinski et al., 2012), we investigated the role of motor thalamus in regulating M1 output during movement. Blocking thalamic input by local infusion of the GABA_A_ receptor agonist muscimol into the VA/VL complex (Experimental Procedures; Figure 4E) enhanced the amplitude of slow, large-amplitude V_m_ fluctuations (control V_m_ SD = 3.8 ± 0.2 mV versus thalamic inactivation SD = 5.1 ± 0.6 mV; n = 45 and n = 6, respectively; p = 3.4 × 10^−2^, Mann-Whitney U test) and produced a hyperpolarizing shift in average V_m_ (control V_m_ = −51.1 ± 0.8 mV versus thalamic inactivation V_m_ = −62.5 ± 3.6 mV; n = 45 and n = 6, respectively; p = 8.0 × 10^−4^), which significantly reduced the basal firing rate of L5B neurons compared to control conditions (control 5.7 ± 0.6 Hz versus thalamic inactivation 1.1 ± 0.5 Hz; n = 45 and n = 6, respectively; p = 1.1 × 10^−3^; Figures 4G and 4H; see also Figure 1). The hyperpolarization associated with thalamic inactivation increased the distance to threshold (data not shown) such that movement-related firing rate changes were abolished (Figure 4G), precluding the functional classification of L5B_supp_ and L5B_enh_ neurons. However, during movement 50% of L5B neurons (n = 3/6) still experienced a 5–10 mV depolarization in mean V_m_ (Figure 4I) and increased rate of compound EPSPs (Figure S5), similar to that observed in L5B_enh_ neurons under control conditions (Figures 1 and 3). We analyzed this further by plotting the ΔV_m_ variability across the L5B pyramidal neuron population, measured as the SD of the ΔV_m_ distributions shown in Figure 4I (population ΔV_m_ SD), using bootstrap analysis (10,000 bootstrap replicates; Figure 4J). We found that motor thalamic inactivation did not affect the population ΔV_m_ variability in L5B pyramidal neurons compared to control (Figures 4I and 4J), suggesting input from the motor thalamus—either direct or indirect—is essential for maintaining L5B pyramidal neuron V_m_ near threshold, but is unlikely to be the main source of the increased excitatory drive.

### Noradrenergic Neuromodulation Selectively Enhances Excitatory Drive and Signal-to-Baseline Ratio in L5B_enh_ Neurons

**Figure 5:**
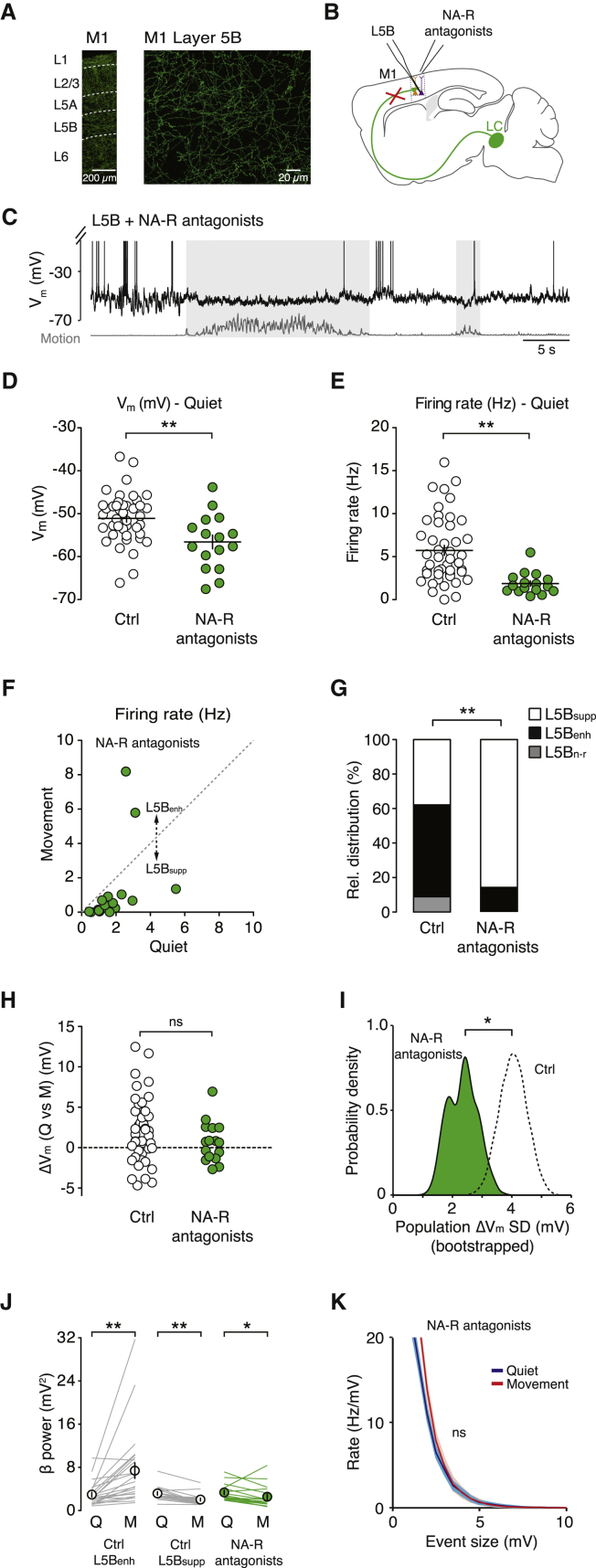
Blocking Input from the LC Reduces Behavioral State-Dependent Increase in Excitatory Drive in L5B_enh_ Neurons (A) Noradrenergic axons in the forelimb region of M1 were labeled using an anti-noradrenaline transporter antibody and secondary antibody conjugated to AlexaFluor 488. (B) Schematic representation of an L5B pyramidal neuron recording after blocking noradrenergic input from the LC. (C) Representative voltage trace from a L5B pyramidal neuron in the absence of noradrenergic input. (D and E) Average V_m_ (D) and firing rate (E) of L5B pyramidal neurons during quiet wakefulness in the absence (open symbols, n = 45) and presence (green symbols, n = 16) of noradrenergic receptor (NA-R) antagonists. Filled circles represent data from individual neurons, black bars represent mean ± SEM. Control data (Ctrl) were taken from the dataset presented in Figure 1 for comparison. Mann-Whitney U test, ^∗^p < 0.017, ^∗∗^p < 0.003. (F) Average firing rate of L5B pyramidal neurons in the presence of noradrenergic receptor antagonists (n = 16) during quiet wakefulness and movement. Filled circles represent data from individual neurons. (G) Relative distributions of L5B_supp_, L5B_enh_, and L5B_n-r_ neurons in the absence (Ctrl) and presence (NA-R antagonists, n = 16) of noradrenergic receptor antagonists. Control data (Ctrl) were taken from the dataset presented in Figure 1 for comparison. Chi-square test, ^∗∗^p < 0.01. (H) Change in average V_m_ (ΔV_m_) during movement in the absence (Ctrl, n = 41) and presence of noradrenergic receptor antagonists (green symbols, n = 16). Control data (Ctrl) were taken from Figure 1 for comparison. Mann-Whitney U test; ns, non-significant. (I) Probability density distributions of ΔV_m_ variability across the L5B pyramidal neuron population (Ctrl and NA-R antagonists), measured as the SD of the ΔV_m_ distributions shown in (H) (Population ΔV_m_ SD) using bootstrap analysis (10,000 bootstrap replicates). Black dashed line represents population ΔV_m_ variability distribution in control (Ctrl), and green shading represents population ΔV_m_ variability distribution following noradrenergic receptor blockade. Control data (Ctrl) were taken from Figure 1 for comparison. F test, ^∗^p < 0.025. (J) Average L5B pyramidal neuron V_m_ power in the β frequency band (12–30 Hz) during quiet wakefulness and movement in the presence (Ctrl: L5B_supp_, n = 17 and L5B_enh_, n = 24) and absence of noradrenergic input (NA-R antagonists, n = 16). Solid lines represent data from individual neurons, symbols represent mean ± SEM. Control data (Ctrl) were taken from Figure 3 for comparison. ^∗^p < 0.05. (K) Average rate density of compound synaptic events in L5B pyramidal neurons during quiet wakefulness (blue) and movement (red) in the absence of noradrenergic input (n = 16). Compare with Figures 3I and 3J. See also Figures S5 and S6.

Given that the movement-related increase in excitatory drive and tonic depolarization in L5B_enh_ neurons could not be directly explained by increased excitation from L2/3 or motor thalamus, we next explored the role of noradrenergic neuromodulation, which has been shown to be important during changes in arousal, attention, and behavioral state (Berridge and Waterhouse, 2003; Carter et al., 2010; Castro-Alamancos and Gulati, 2014; Constantinople and Bruno, 2011; Polack et al., 2013). Selective immunohistochemical staining for the noradrenaline transporter (NAT), expressed exclusively in noradrenergic axons (Lorang et al., 1994), revealed dense axonal innervation of all layers in forelimb M1 (Figure 5A). To test the importance of noradrenergic input in regulating L5B pyramidal neuron V_m_ dynamics during movement, we topically applied α1, α2, and β noradrenergic receptor antagonists (1 mM prazosin, 1 mM yohimbine, and 1 mM propranolol, respectively) to the forelimb region of M1 (Figures 5B and 5C). The local infusion of noradrenergic receptor antagonists via the craniotomy selectively disrupted noradrenergic signaling in forelimb M1 (Figure S6), whereas direct manipulation of LC activity, via electrical stimulation or optogenetics, would have widespread effects across many brain areas and spinal cord circuits. Moreover, topical application was preferred due to the technical limitations of simultaneously pressure ejecting drugs at multiple sites along the entire somatodendritic length of L5B pyramidal neurons during intracellular recordings.

Blocking noradrenergic receptors reduced the mean V_m_ (control −51.1 ± 0.8 mV versus noradrenergic receptor blockade −56.6 ± 1.6 mV; p = 4.0 × 10^−3^) and quiet wakefulness firing rate of L5B pyramidal neurons (control 5.7 ± 0.6 Hz versus noradrenergic receptor blockade 1.9 ± 0.3 Hz; n = 45 and n = 16, respectively; p < 1 × 10^−4^; Figures 5D and 5E), and significantly reduced the proportion of L5B neurons that displayed enhanced firing rates during movement (control L5B_enh_ 24/45 neurons [53.3%] versus noradrenergic receptor blockade L5B_enh_ 2/16 neurons [12.5%]; p < 1 × 10^−2^; Figures 5F and 5G). The change in relative distribution of L5B_supp_/L5B_enh_ neurons could be explained in part by the moderate hyperpolarization in V_m_ and increased distance to threshold during movement (Figure S6). Although noradrenergic receptor blockade did not affect the mean population ΔV_m_ compared to control conditions, due to both distributions being centered around 0 mV (Figure 5H), we did observe a significant decrease in ΔV_m_ variability across the L5B pyramidal neuron population, measured as the SD of the ΔV_m_ distributions shown in Figure 5H (population ΔV_m_ SD) using bootstrap analysis (10,000 bootstrap replicates; Figure 5I). Consistent with the idea that noradrenergic signaling underpins a large proportion of the increased excitatory drive to L5B_enh_ neurons during movement, blocking noradrenergic receptors also abolished the increase in V_m_ β-band power (Figure 5J) and rate of compound synaptic events associated with movement (Figures 5K and S5).

**Figure 6:**
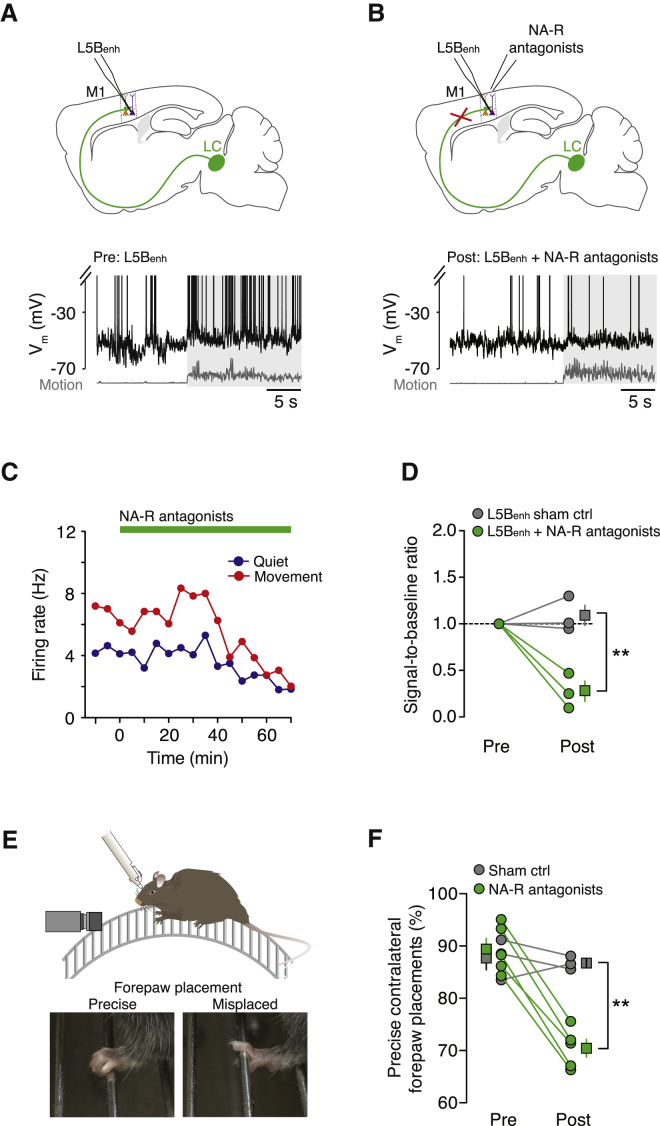
Blocking Noradrenergic Input Reduces the SBR in L5B_enh_ Neurons and Impairs Contralateral Forepaw Motor Coordination (A and B) Schematic representation of the experimental design and representative voltage traces from an L5B_enh_ pyramidal neuron prior to (A) and after (B, >30 min) topical application of noradrenergic receptor antagonists. Gray shading depicts movement. (C) Time course shows quiet (blue) and movement-related (red) firing rates in an L5B_enh_ pyramidal neuron before and after noradrenergic receptor blockade (green bar). (D) Movement-induced SBR in L5B_enh_ pyramidal neurons before and >30 min after application of noradrenergic receptor antagonists (green circles, n = 3) or saline (gray circles, n = 3). Square symbols represent mean ± SEM. Unpaired t test, ^∗∗^p < 0.01. (E) Behavioral assessment of forepaw placement precision in head-fixed mice mounted on a single-axis, cylindrical runged treadmill. Video sequences were used to score contralateral and ipsilateral forepaw placements. (F) Percentage of precise contralateral forepaw placements before and 60 min after application of noradrenergic receptor antagonists (green circles, n = 5) or saline (gray circles, n = 3). Square symbols represent mean ± SEM. Unpaired t test, ^∗∗^p < 0.01. See also Figure S6.

Given that pre-application of noradrenergic receptor antagonists precludes the prior identification of L5B_enh_ neurons prior to receptor blockade, we also performed long-term (40- to 80-min) recordings from identified L5B_enh_ neurons before (Figure 6A) and after (Figure 6B) receptor block. If noradrenergic neuromodulation underpins the V_m_ depolarization in L5B_enh_ neurons during movement, then blocking noradrenergic receptors should have a disproportionately larger effect on movement-related firing rates compared to quiet firing rates. Accordingly, we found that receptor blockade resulted in a modest, time-dependent reduction in L5B_enh_ basal firing rates and a strong suppression of movement-related firing (Figure 6C). The drug diffusion and time dependency of the antagonist effects in L5B were consistent with our dye diffusion mapping results (Figure S6). To assess the extent to which noradrenaline facilitates L5B_enh_ output during movement, we examined the Signal-to-Baseline Ratio (SBR), defined as the ratio of the movement-related spike rate to the spike rate during quiet wakefulness. Blocking noradrenergic neurotransmission significantly reduced the SBR compared to control conditions (sham control SBR: 1.1 ± 0.1, noradrenergic receptor antagonist SBR: 0.3 ± 0.1; p = 6 × 10^−3^; n = 3 and 3, respectively; Figure 6D).

Since descending M1 output is essential for maintaining normal locomotor function (Armstrong and Drew, 1984a; Beloozerova et al., 2003; Orlovsky, 1972; Ueno and Yamashita, 2011), we investigated whether there was a behavioral correlate of reduced M1 output during noradrenergic receptor blockade by conducting a series of behavioral experiments using head-restrained mice habituated to walk/run on a cylindrical runged treadmill (Figure 6E). This experimental paradigm facilitates the analysis of precise forepaw placements during locomotion, which was not possible on the conventional single-axis cylindrical treadmill shown in Figure 1A. Although classified as complex locomotion, this paradigm generates only subtle differences in forelimb muscle activity/wrist movements and comparable changes in M1 activity when compared to simple locomotion on a linear treadmill (Beloozerova et al., 2010; Marlinski et al., 2012). Selectively blocking noradrenergic receptors in forelimb M1 significantly decreased the number of precise contralateral forepaw placements compared to sham controls (precise forepaw placements 60 min after: sham saline 86.8% ± 0.7%, noradrenergic receptor antagonists 70.5% ± 1.7%; n = 3 and 5, respectively; p < 4.0 × 10^−4^; Figure 6F) or ipsilateral forepaw placements (data not shown). Together, our results demonstrate that noradrenergic input from the LC is necessary for controlling M1 output and motor coordination during self-paced voluntary movement.

## Discussion

In this paper we present three main findings. First, we show that behavioral state-dependent bidirectional modulation of M1 output is governed by two opposing subthreshold mechanisms (1) a global decrease in network-driven, slow, large-amplitude V_m_ fluctuations, which reduced V_m_ variability, spike probability, and firing rates in L5B_supp_ neurons; and (2) a coincident increase in excitatory drive in a subpopulation of L5B neurons (L5B_enh_), which increased spike probability and firing rates. Second, we demonstrate that the movement-related tonic depolarization in L5B_enh_ neurons requires the interplay between ascending input from the motor thalamus, which maintained V_m_ near threshold, and noradrenergic input from the LC, which enhanced the SBR for movement-evoked responses. Finally, we show that selective blockade of noradrenaline signaling in forelimb M1 reduces motor coordination in the contralateral forelimb, resulting in a significant decrease in precise forepaw placements. Together, our findings reveal the subthreshold and circuit mechanisms that regulate behavioral state-dependent bidirectional modulation of M1 output during self-paced, voluntary movement.

### Behavioral State-Dependent Modulation of Input-Output Transformations in L5

Physiologically relevant changes in V_m_ variance or mean have been shown to profoundly influence neuronal input-output transformations (Chance et al., 2002; Hô and Destexhe, 2000). But, this has never been explored in L5 pyramidal neurons in the awake cortex. Our current injection experiments in vivo demonstrate that changes in V_m_ SD (L5B_supp_) or V_m_ SD and mean (L5B_enh_) have quantitatively similar—but functionally opposing—effects on spike probability when examined over a behaviorally relevant input amplitude range (1–10 mV). This similarity arises due to the non-linear relationship between V_m_ and firing probability, such that moderate depolarization can produce a non-linear additive increase in the sensitivity of a neuron to small-amplitude inputs, while decreased V_m_ SD produces a divisive reduction in input sensitivity (Brozović et al., 2008; Murphy and Miller, 2003). The behavioral state-dependent bidirectional modulation of neuronal responsiveness in L5B pyramidal neurons (i.e., increased or decreased spike probability) could facilitate the routing of sensorimotor information through specific M1 neuronal assemblies during movement.

### Local and Long-Range Inputs to M1 during Self-Paced Voluntary Movement

M1 receives input from a variety of brain areas (e.g., ipsilateral primary and secondary somatosensory cortices, secondary motor cortex, and orbitofrontal cortex), with ascending input from motor thalamus and descending input from L2/3 providing strong feedforward excitation directly to L5B neurons (Castro-Alamancos and Connors, 1996; Hooks et al., 2013; Weiler et al., 2008). We found that movement did not affect firing rates in our sample of L2/3 pyramidal neurons, suggesting that descending excitation from L2/3 may not be the primary source of the tonic depolarization in L5B_enh_ neurons during simple locomotion on a cylindrical treadmill. These findings are in direct contrast to a previous study by Dombeck and colleagues, where locomotion on a spherical treadmill resulted in large-scale, clustered activity of L2/3 neurons in mouse forelimb M1 (Dombeck et al., 2009). The reason for this discrepancy is unclear. One possibility is that our recordings undersampled L2/3 population activity; however, if locomotion induced dense activity similar to that observed in Dombeck et al., (2009), we would have expected to observe movement-related firing rate changes in a significant proportion of our intracellular recordings. Moreover, Dombeck and colleagues did not identify individual neuronal subtypes, so the large-scale activity could be due, in part, to elevated L2/3 interneuron activity. Alternatively, dense L2/3 activity could result from mice having to balance and oppose the inertial forces of a rotating air-cushioned ball when changing direction. In principle, this could generate a sensorimotor mismatch between the rotational direction of the ball and the intended movement trajectory of the mouse, leading to continuous sensory feedback to M1. Thus, it will be important for future studies to investigate the extent to which descending L2/3 input contributes to L5B frequency modulation during simple versus complex motor behaviors.

Direct thalamic input to cortical pyramidal neurons can drive output by reducing slow V_m_ fluctuations, depolarizing mean V_m_, and reducing the distance to threshold (Castro-Alamancos and Connors, 1996; Constantinople and Bruno, 2013; Hirata and Castro-Alamancos, 2010; Poulet et al., 2012). Consistent with previous findings in sensory cortex, we found that inactivation of thalamus (VA/VL region) increased slow, large-amplitude V_m_ fluctuations, but did not abolish the activated state during behavior (Hirata and Castro-Alamancos, 2010; Poulet et al., 2012), suggesting thalamic input to M1 is sufficient but not necessary for generating the activated cortical state. However, ascending motor thalamic input—direct or indirect—appears to be necessary for maintaining the average V_m_ relatively close to threshold, providing a mechanism whereby subtle changes in input structure can generate positive or negative changes in M1 output during movement.

### Noradrenergic Neuromodulation

We have shown that noradrenaline release during different behavioral states (i.e., quiet wakefulness versus movement) has profound effects on M1 cortical dynamics. Similar to thalamic inactivation, blocking noradrenergic input from the LC reduced basal firing rates by hyperpolarizing mean V_m_ and increasing distance to threshold, suggesting tonic input from both the LC and motor thalamus are necessary to generate moderate firing rates in L5B pyramidal neurons during quiet wakefulness. Our finding that noradrenaline generated a tonic depolarization in a selected subpopulation of L5B pyramidal neurons differs from results obtained in superficial layers of sensory cortex (Polack et al., 2013), highlighting the importance of understanding the sublayer-specific effects of noradrenaline in the awake cortex. In primary visual cortex (V1), locomotion-dependent noradrenaline release generates a global depolarization of L2/3 pyramidal neurons, which may enhance visual attention by increasing the gain and signal-to-noise ratio of visually evoked responses (Bennett et al., 2013; Polack et al., 2013).

The fact that we also observed a movement-related tonic depolarization in the majority of M1 L2/3 pyramidal neurons, which was abolished by noradrenergic receptor blockade (Figure S6), suggests that noradrenaline may differentially affect cortical processing in superficial versus deep-layer pyramidal neurons during active behavior. Topical application of high concentrations of noradrenergic receptor antagonists could potentially produce off-target effects. However, given that low doses of antagonists affect L2/3 V_m_ dynamics in the same way as high concentrations, albeit smaller in magnitude, suggests relatively selective antagonist effects (Polack et al., 2013). Although noradrenaline appears to underpin the majority of the locomotion-dependent V_m_ depolarization in V1, cholinergic disinhibition of somatostatin-containing interneurons is likely to further enhance behavioral state-dependent gain modulation (Fu et al., 2014). We did not directly test the role of acetylcholine in our study, but given its importance in regulating V_m_ dynamics in other cortical areas (Constantinople and Bruno, 2011; Eggermann et al., 2014; Favero et al., 2012; Fu et al., 2014; Polack et al., 2013), it will be important for future studies to investigate its role in M1 during motor behavior.

How does noradrenaline generate the tonic depolarization in L5B_enh_ neurons during movement? Previous studies have shown that noradrenaline modulates voltage-dependent and voltage-independent potassium conductances and hyperpolarization-activated cyclic nucleotide-gated (HCN) channels, thus generating a tonic depolarization by reducing the spike after-hyperpolarization and prolonging the depolarizing effect of excitatory synaptic inputs (Favero et al., 2012; Sheets et al., 2011; Wang et al., 2007; Wang and McCormick, 1993). This combined with modulation of basal firing rates is thought to alter the signal-to-noise ratio of neuronal responses to synaptic input (Berridge and Waterhouse, 2003). Alternatively, we cannot rule out the possibility that noradrenaline selectively reduces the activity of local GABAergic interneurons, thus releasing L5B_enh_ neurons from inhibition and generating a depolarization in V_m_. Therefore, identifying the specific expression patterns and subcellular localization of α and β adrenergic receptors in excitatory and inhibitory neurons in M1 will be an important next step in understanding how noradrenaline exerts its sublayer- and cell-type specific effects.

### Functional Implications

What function does behavioral state-dependent bidirectional modulation of L5 output serve? The flexible modulation of L5B output channels (PT type and IT type) provides an important control mechanism to modulate and update activity patterns in downstream cortical and subcortical areas during changes in behavioral state. PT output provides online information about the state of cortical activation to downstream areas involved in motor control. This continuously updating flow of information generates a basic pattern of input to brainstem and spinal cord circuits in order to generate appropriate behavioral responses in accordance with changes in behavioral state. We demonstrate that blocking noradrenergic receptors in forelimb M1 selectively disrupts motor coordination in the contralateral forepaw, thus confirming the importance of noradrenergic neuromodulation and descending M1 output for motor control. Given that output from sensory and non-sensory cortices have overlapping downstream targets (Hattox and Nelson, 2007; Kita and Kita, 2012), we speculate that our findings might generalize to other cortical output layers and that noradrenergic neuromodulation and network-driven input changes are common mechanisms to bidirectionally modulate cortical output during active behavior.

## Experimental Procedures

### Animals and Surgery

All experiments and procedures involving animals were approved by the University of Edinburgh local ethical review committee and performed under license from the UK Home Office in accordance with the Animal (Scientific Procedures) Act 1986. Male C57BL/6 mice (5–12 weeks old, 20–25 g, two to six animals per cage, maintained on a reversed 12:12-hr light:dark cycle with ad libitum access to food and water) were implanted with a small lightweight headplate (0.75 g) using cyano-acrylate glue and dental acrylic. All surgical procedures were performed under 1.5% isoflurane anesthesia. After 24-to 48-hr recovery, a craniotomy (300 × 300 μm) was performed and the dura removed above the right forelimb region of M1. Using intracortical microstimulation (see Supplemental Experimental Procedures), the center of M1_FL_ was located 0.7 mm rostral and 1.5 mm lateral to bregma. The craniotomy was sealed with (1.5%) agar and Kwik-Cast sealant (WPI) and mice recovered for 2 hr before recording commenced.

### Motion Index and Motor Pattern Discrimination

An optical encoder was used to capture movement of the treadmill and locomotion was defined as periods with speed > 0.01 m/s for more than 2 s. Changes in behavioral state (quiet wakefulness to movement [grooming or locomotion]) were captured using an elevated, front-mounted, moderate-speed (60 frames/s) digital video camera synchronized with each electrophysiological recording.

### In Vivo Electrophysiology and Pharmacology

Mice were habituated to the head restraint and experimental setup for 45–60 min before each recording session. Whole-cell patch-clamp recordings were obtained from awake head-restrained mice at a depth of 180–420 μm (layer 2/3) or 620–880 μm (layer 5B) from the pial surface, using a Multiclamp 700B amplifier (Molecular Devices). The signal was filtered at 10 kHz and acquired at 20 kHz using PClamp 10 software in conjunction with a DigiData 1440 DAC interface (Molecular Devices). No bias current was injected during recordings and the membrane potential was not corrected for junction potential. Resting membrane potentials were recorded immediately after attaining the whole-cell configuration (break-in). Series resistances (R_s_) ranged from 15 to 40 MΩ and experiments were terminated if R_s_ exceeded 60 MΩ. Current injection was performed only if R_s_ < 35 MΩ. Patch pipettes (5–7 MΩ) were filled with internal solution (285–295 mOsm) containing: 135 mM K-gluconate, 4 mM KCl, 10 mM HEPES, 10 mM sodium phosphocreatine, 2 mM MgATP, 2 mM Na_2_ATP, 0.5 mM Na_2_GTP, and 2 mg/ml biocytin (pH adjusted to 7.2 with KOH). External solution contained: 150 mM NaCl, 2.5 mM KCl, 10 mM HEPES, 1 mM CaCl_2_, and 1 mM MgCl_2_ (adjusted to pH 7.3 with NaOH).

For inactivation of the motor thalamus, the GABA_A_ receptor agonist muscimol (1 mM muscimol hydrobromide, Sigma-Aldrich) was dissolved in external solution, and 100 nl was stereotaxically injected into the right VA/VL complex (−1 mm caudal, 1.1 mm lateral to bregma, and 3.2 mm below the pial surface). Whole-cell patch-clamp recordings of L5B pyramidal neurons were carried out approximately 2 hr after muscimol injection.

To block noradrenergic receptors, a mixture of α1, α2, and β noradrenergic receptor antagonists (1 mM prazosin, yohimbine, and propranolol; Sigma-Aldrich) in external solution (adjusted to pH 7.3) was applied topically to the craniotomy and recordings were performed >40 min after antagonist application.

### Functional Classification of Recorded Neurons

For each L5B cell, we (1) divided quiet periods into 1-s epochs; (2) randomly assigned each epoch into two groups, quiet 1 (q1) and quiet 2 (q2); and (3) calculated the firing rate difference between q1 and q2. We repeated steps (1) to (3) 10,000 times for each cell to obtain the distribution probability of the difference of firing rate in q1 and q2 (see Figures 1C–1E). If during movement the firing rate change was higher than the 99^th^ percentile or lower than the 1^st^ percentile, we classified the neuron as enhanced or suppressed, respectively. If the firing rate change fell within the first and 99^th^ percentiles, the cell was classified as non-responding.

### Statistical Analyses

Summary data are expressed as mean ± SEM unless otherwise stated. Statistical significance was determined using Wilcoxon signed-rank tests (paired data) and rank-sum tests (unpaired data) unless otherwise stated. Wilcoxon signed-rank tests on the areas underlying the rate-density curves were used in Figures 3I and 3J. The relative distribution of functional phenotypes (L5B_supp_, L5B_enh_, and L5B_n-r_) was analyzed using Pearson chi-square test statistics (based on 10^6^ permutations). Statistical significance in population ΔV_m_ variability (Figures 4J and 5I) was determined using two-sample F tests. To depict the variance of the underlying populations, 10,000 bootstrap samples (random samples with replacement) of each population were taken, and a probability density function of the variances of the bootstrap samples was plotted. For statistical tests, p < 0.05 was considered significant (^∗^p < 0.05 and ^∗∗^p < 0.01). For repeated statistical comparisons with the control dataset, resulting p values were compared to Bonferroni-corrected alpha levels and stated accordingly.

## Author Contributions

J.S., P.P., M.C.W.v.R., and I.D. designed the experiments. All experiments were carried out by J.S., P.P., and J.D. with help from I.D. Analysis was performed by P.P., J.S., M.P., and J.D. A.D. wrote MATLAB scripts to extract forepaw motion statistics. Neuronal reconstructions were carried out by J.S., P.P., J.D., and I.D. I.D., P.P., and J.S. wrote the manuscript, and all authors contributed to discussion and interpretation of the results.

## Supplemental Information

Document S1. Supplemental Experimental Procedures, Figures S1–S6, and Tables S1 and S2

Document S2. Article plus Supplemental Information
