# Supplementary material for: Cellular Mechanisms Underlying Behavioral State-Dependent Bidirectional Modulation of Motor Cortex Output
Source: Cell Rep. 2015 May 14;11(8):1319–30. doi: 10.1016/j.celrep.2015.04.042 (PMC4451462; doi:10.1016/j.celrep.2015.04.042)
Supplement: Document S1. Supplemental Experimental Procedures, Figures S1–S6, and Tables S1 and S2 [file mmc1.pdf]

Cell Reports

Supplemental Information

Cellular Mechanisms Underlying Behavioral State-Dependent Bidirectional Modulation of Motor Cortex Output

Julia Schiemann, Paolo Puggioni, Joshua Dacre, Miha Pelko, Aleksander Domanski, Mark C.W. van Rossum, and Ian Duguid

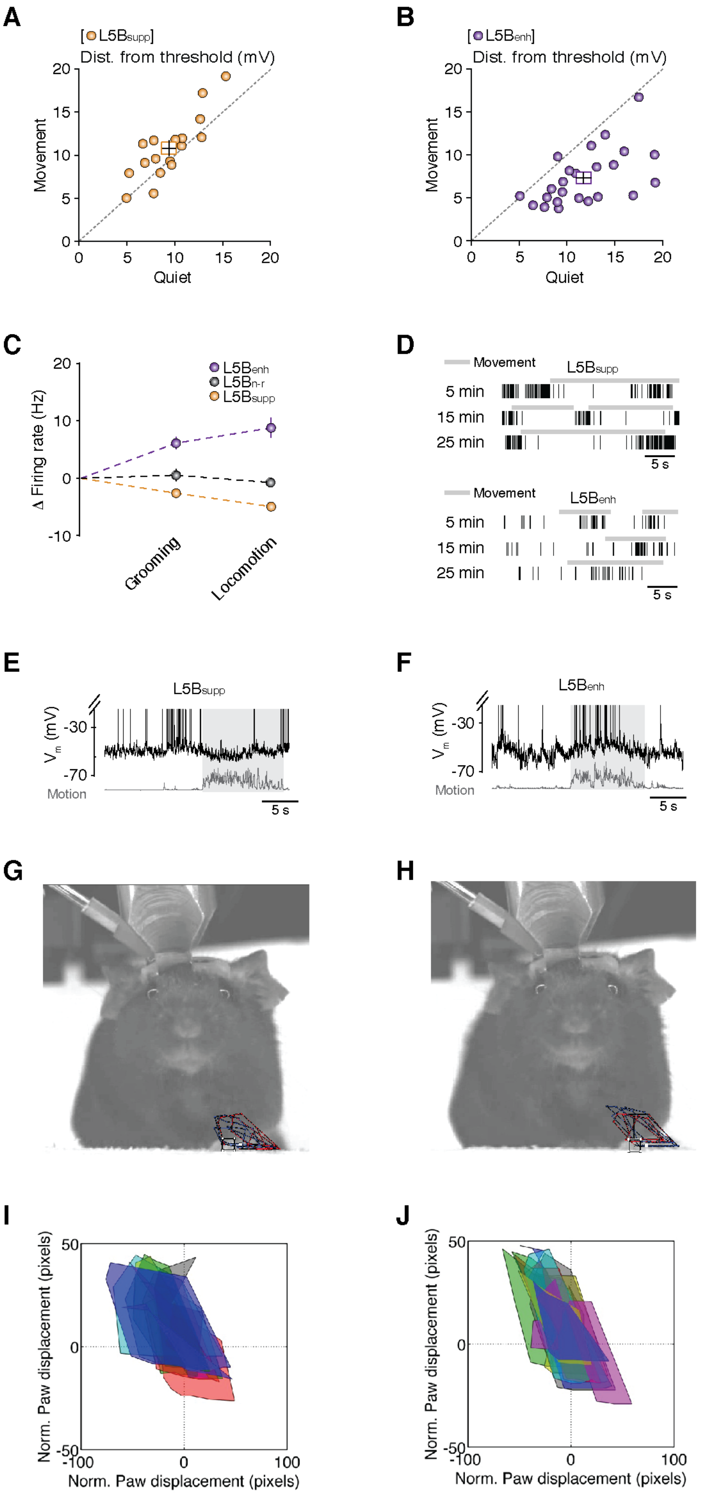

Figure S1. Membrane potential dynamics of $L5B_{supp}$ and $L5B_{enh}$ neurons during quiet wakefulness and self-paced voluntary movement. Related to Figure 1.

(**A-B**) Average distance from threshold in $L5B_{supp}$ (A, *n* = 17) and $L5B_{enh}$ (B, *n* = 24) pyramidal neurons during quiet wakefulness and movement. Filled circles represent data from individual neurons while square symbols represent mean ± s.e.m..

(**C**) Average firing rate changes in $L5B_{supp}$ (yellow, n = 17), $L5B_{non\text{-}responding}$ (gray, n = 4) and $L5B_{enh}$ (purple, n = 24) neurons during grooming or locomotion.

(**D**) Representative spike raster plots from a $L5B_{supp}$ (upper panel) and a $L5B_{enh}$ (lower panel) neuron during movement phases occurring 5, 15 and 25 minutes after 'break-through'. Note functional classification of neurons is unchanged over time.

(**E-F**) Representative voltage traces from a $L5B_{supp}$ (E) and $L5B_{enh}$ (F) pyramidal neuron recorded in the same mouse during similar forelimb movements (gray shading) as characterized in G-J.

(**G-H**) Video tracking of contralateral forelimb movements – repeated swing/stance cycles – associated with activity in a $L5B_{supp}$ (G) and $L5B_{enh}$ (H) neuron. Tracking lines represent similar forelimb movements during the 25 frames preceding (blue) and 25 frames after (red) the present video frame.

(**I-J**) Extracted vertical and horizontal paw displacements of individual contralateral forelimb step cycles associated with activity in a $L5B_{supp}$ (I) and $L5B_{enh}$ (J) neuron. Individual steps are represented by different colors. Quantification of the similarity of forelimb movements identified no significant difference. Step path circumference: $L5B_{supp}$ neuron 211 ± 15 pixels, $L5B_{enh}$ 238 ± 16 pixels, p = 0.23. Step path area: $L5B_{supp}$ neuron 2018 ± 313 pixels, $L5B_{enh}$ 2197 ± 243 pixels, p = 0.65.

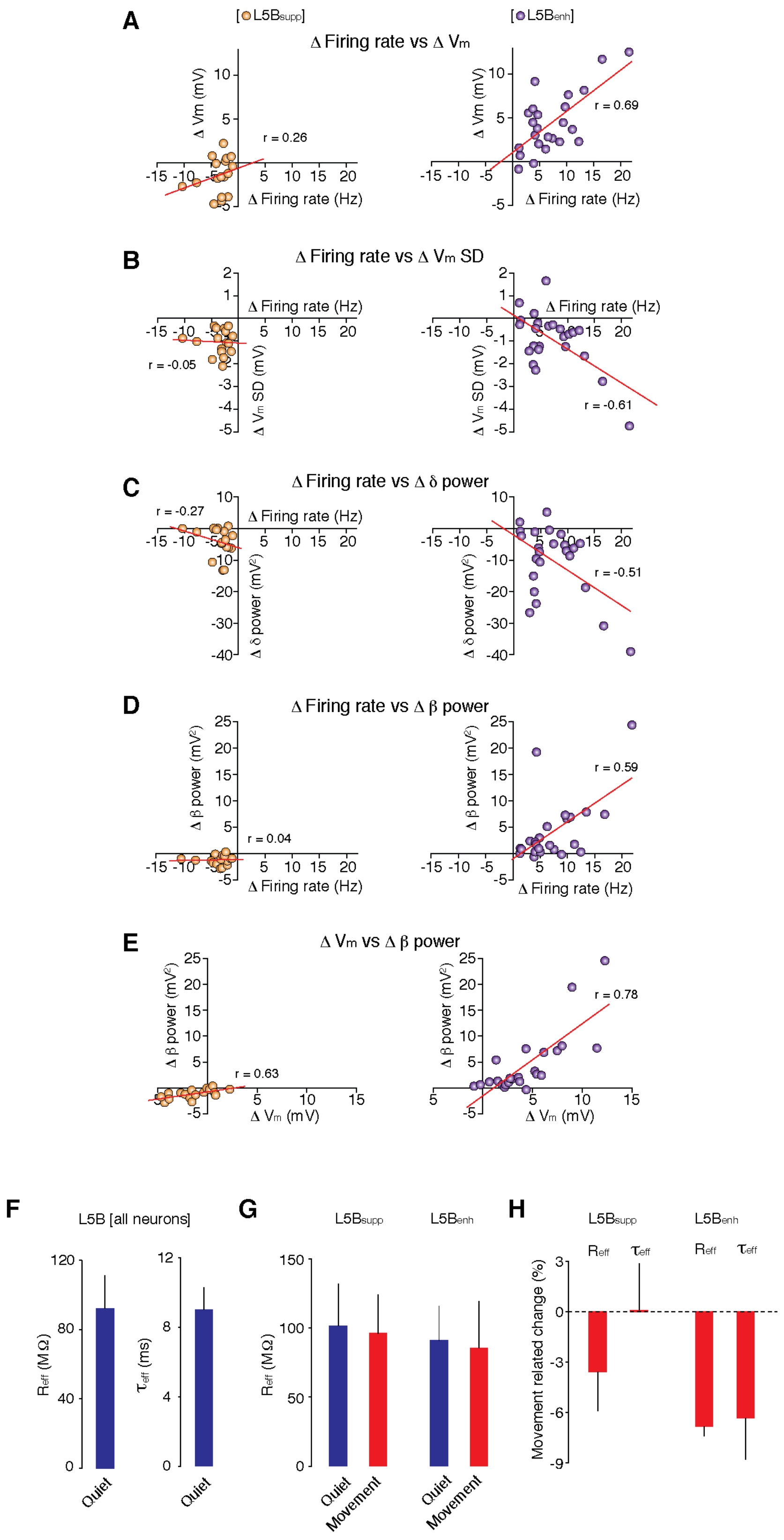

Figure S2. Movement-related changes in subthreshold V_m dynamics, firing rates, effective input resistance and membrane time constant in L5B pyramidal neurons. Related to Figures 1, 2 and 3.

(**A-D**) Relationship between Δ firing rate and Δ V_m (A), Δ V_m SD (B), Δ V_m power in δ (1.5 – 4 Hz, C) and β (12 – 30 Hz, D) frequency bands in $L5B_{supp}$ (yellow, *n* = 17) and $L5B_{enh}$ (purple, *n* = 24) neurons during movement.

(**E**) Relationship between Δ V_m and Δ V_m power in β (12 – 30 Hz) frequency band in $L5B_{supp}$ (yellow, *n* = 17) and $L5B_{enh}$ (purple, *n* = 24) neurons during self-paced voluntary movement.

(**F**) Input resistance (R_{eff}, left) and membrane time constant (τ_{eff}, right) of L5B neurons (*n* = 10) during quiet wakefulness.

(**G**) Input resistance of $L5B_{supp}$ (*n* = 5, left) and $L5B_{enh}$ (*n* = 5, right) pyramidal neurons during quiet wakefulness (blue) and movement (red).

(**H**) Movement-related changes in effective input resistance (R_{eff}) and membrane time constant (τ_{eff}) for $L5B_{supp}$ (*n* = 5) and $L5B_{enh}$ (*n* = 5) pyramidal neurons. Bars represent median values ± median absolute deviation.

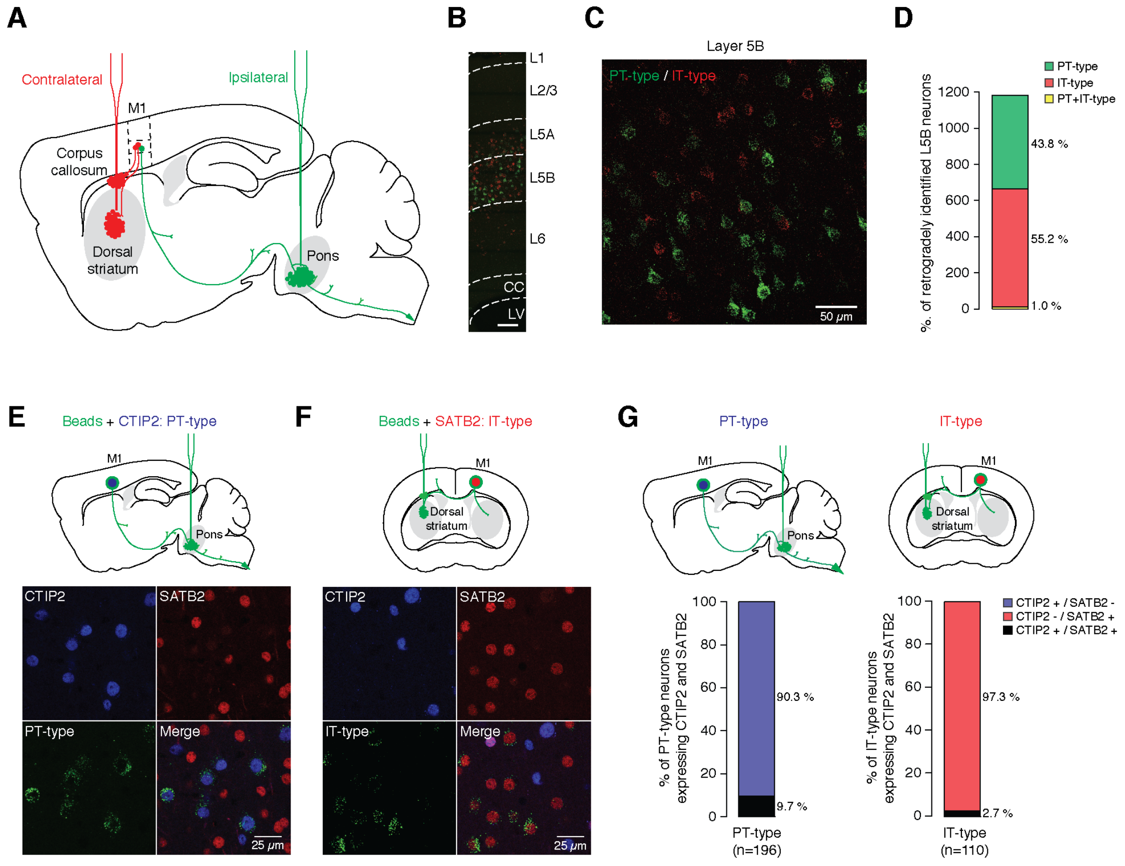

Figure S3. Classification of L5B projection neurons based on retrograde tracing and molecular marker expression. Related to Figure 1.

(**A**) Schematic parasagittal brain section illustrating retrograde fluorescent bead injection sites. Red fluorescent beads were injected into the contralateral dorsal striatum and corpus callosum to identify intratelencephalic (IT-type) projection neurons. Green fluorescent beads were injected into the ipsilateral pons to identify pyramidal tract (PT-type) projection neurons.

(**B-C**) Distribution of PT-type (green) and IT-type (red) projection neurons across different cortical layers (B) and within L5B (C) of the forelimb region of M1. LV: lateral ventricle; CC: corpus callosum; scale bar 100 μm.

(**D**) Relative distributions of PT-type (green) and IT-type (red) projection neurons in L5B of forelimb motor cortex (n = 1183 neurons, 50 slices, 6 mice).

(**E-F**) Single retrograde tracing and immunohistochemical staining confirming transcription factors CTIP2 and SATB2 are molecular markers for retrogradely identified PT-type (ipsilateral pons injection, E) and IT-type (contralateral dorsal striatum injection, F) neurons, respectively.

(**G**) Relative distribution of retrogradely identified L5B neurons expressing CTIP2 or SATB2 (n = 306 neurons, 24 slices, 4 mice).

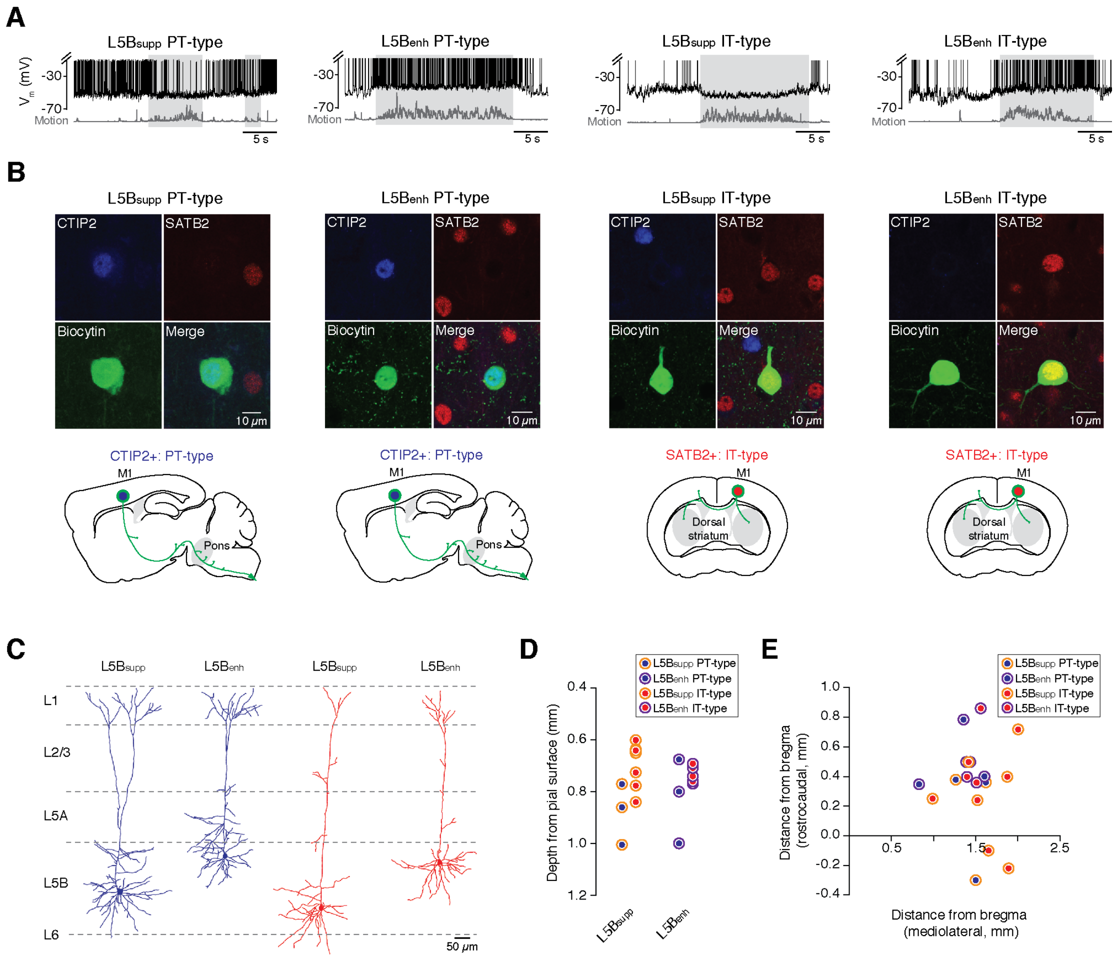

Figure S4. Projection class identity of individually recorded $L5B_{supp}$ and $L5B_{enh}$ neurons. Related to Figure 1.

(**A**) Representative voltage traces from identified: $L5B_{supp}$ PT-type; $L5B_{enh}$ PT-type; $L5B_{supp}$ IT-type; and $L5B_{enh}$ IT-type during quiet wakefulness and movement (light gray shading and dark gray motion index).

(**B**) Upper panels: single cell biocytin labeling (green) and post-hoc immunohistochemical staining for CTIP2 (blue) and SATB2 (red) confirmed the projection class identity of the individually recorded L5B pyramidal neurons shown in panel A. Lower panels: schematic brain sections showing $L5B_{supp}$ and $L5B_{enh}$ neurons contain a mixture of PT-type and IT-type projection neurons.

(**C**) Representative morphological reconstructions of $L5B_{supp}$ and $L5B_{enh}$ neurons color-coded by projection class identity (blue = PT-type, red = IT-type).

(**D**) Average depth of individually recorded $L5B_{supp}$ (n = 10) and $L5B_{enh}$ (n = 8) neurons color-coded by projection class identity.

(**E**) Neuroanatomical coordinates of individually recorded L5B pyramidal neurons plotted according to their position relative to bregma (n = 10 $L5B_{supp}$ neurons; n = 8 $L5B_{enh}$ neurons).

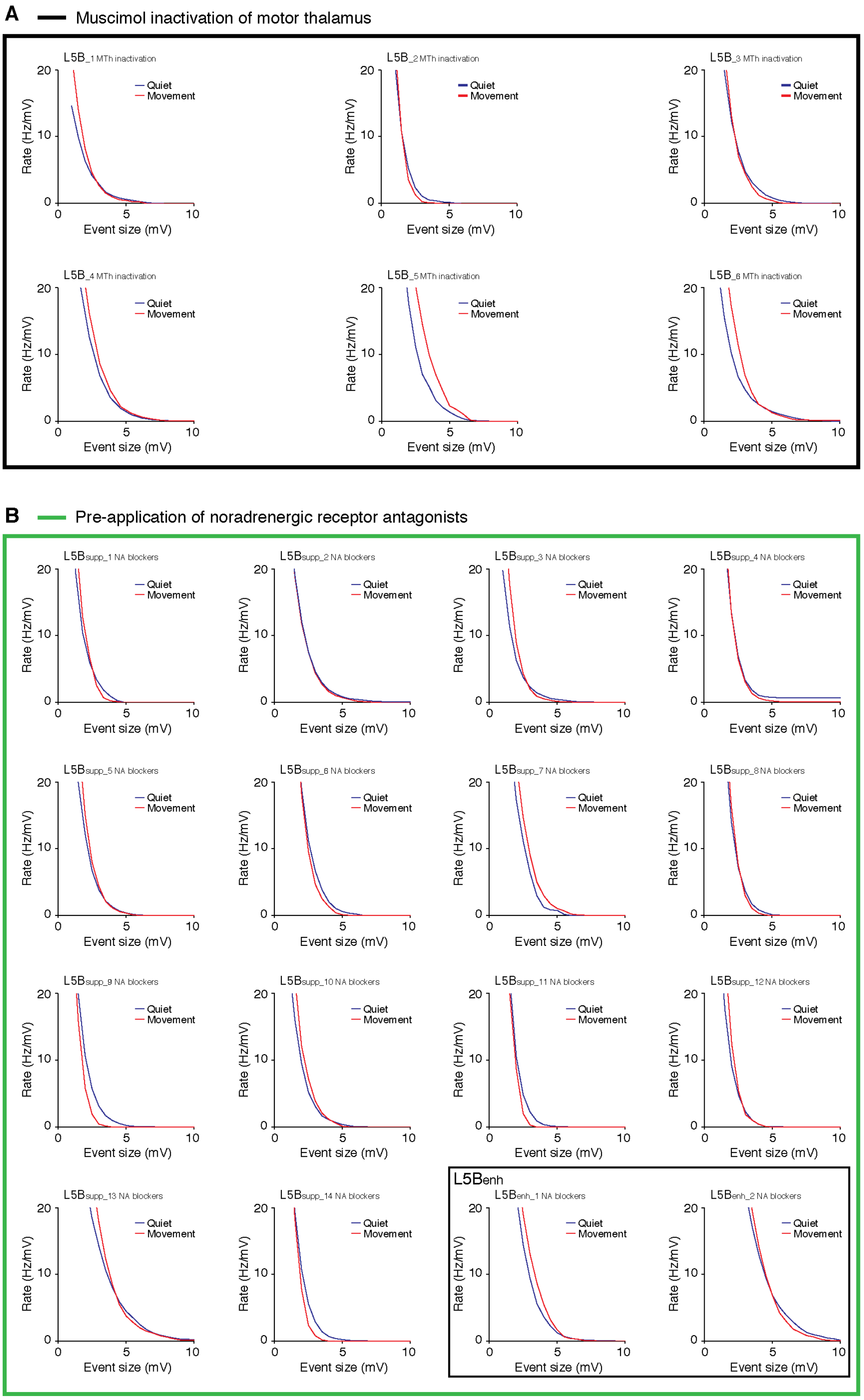

Figure S5. Compound event detection in L5B pyramidal neurons in the absence of motor thalamic input or noradrenergic input. Related to Figures 4 and 5.

(**A**) Average rate density of compound synaptic events in individual L5B pyramidal neurons (*n* = 6) during quiet wakefulness (blue) and movement (red). Note upper three panels depict neurons in which there was no change in rate density during movement, while bottom three panels depict cells that show an increased frequency of events.

(**B**) Average rate density of compound synaptic events in individual $L5B_{supp}$ (*n* = 14) and $L5B_{enh}$ (black box, *n* = 2) pyramidal neurons during quiet wakefulness (blue) and movement (red) in the absence of noradrenergic input. Note that the majority of L5B neurons do not show a significant increase in rate density during movement.

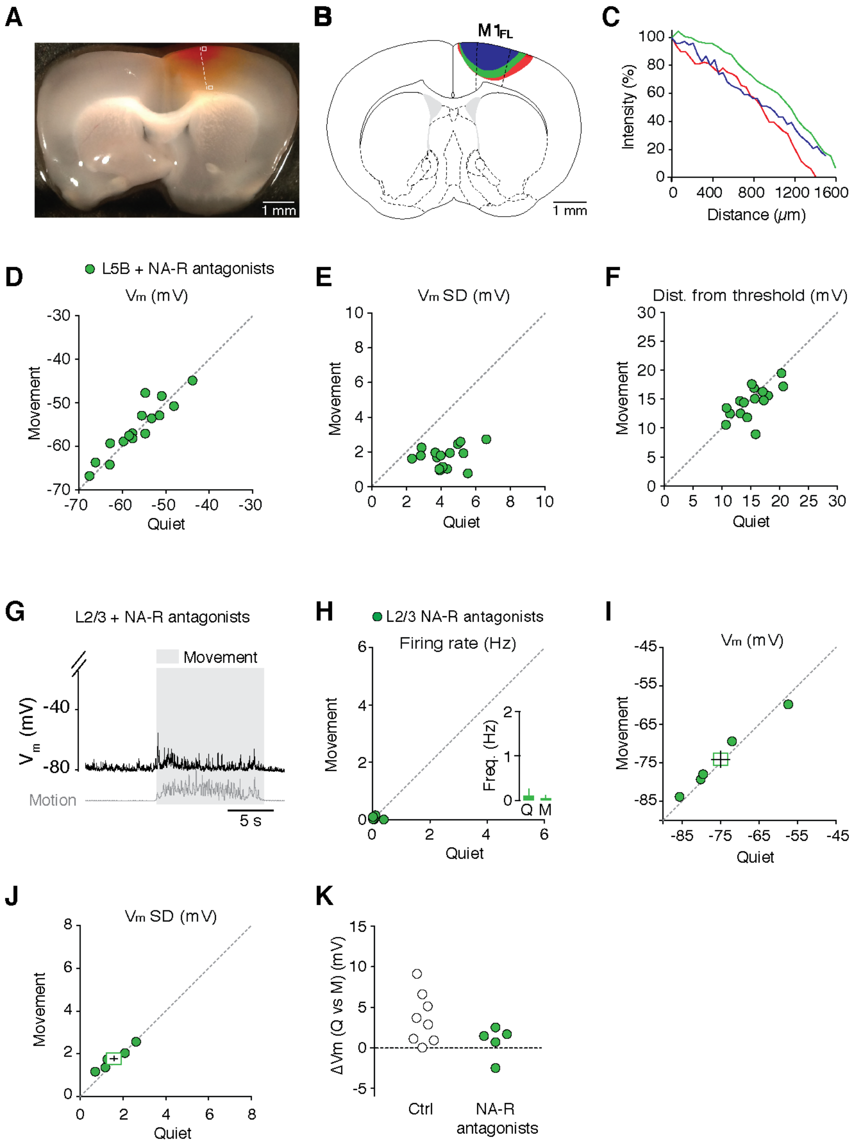

Figure S6. Drug diffusion through cortical layers and effects of noradrenergic receptor blockade on V_m dynamics of L5B and L2/3 pyramidal neurons during quiet wakefulness and movement. Related to Figures 5 and 6.

(**A**) Brightfield image showing dye diffusion through cortical layers after 60-minute application of phenol red (10-50 mM) to the surface of $M1_{FL}$.

(**B**) Schematic coronal brain section depicting dye diffusion through cortical layers after 60-minute application of phenol red (10-50 mM) to the surface in N = 3 mice. Note dye diffusion is centered around $M1_{FL}$ and does not penetrate subcortical areas.

(**C**) Dye intensity plotted as function of distance from the surface of the cortex (see dashed line in panel A).

(**D-F**) Average V_m (D), V_m SD (E) and distance from threshold (F) in L5B pyramidal neurons (green symbols, *n* = 16) during quiet wakefulness and movement in the absence of noradrenergic input. Filled circles represent data from individual neurons.

(**G**) Representative voltage trace from a L2/3 pyramidal neuron in the absence of noradrenergic input. Gray shading denotes movement.

(**H-J**) Average firing rate (H), V_m (I) and V_m SD (J) in L2/3 pyramidal neurons during quiet wakefulness and movement in the absence of noradrenergic input (n = 5). Filled circles represent data from individual neurons while square symbols represent mean ± s.e.m.. Inset in (H) depicts the average L2/3 neuron firing rate during quiet wakefulness (Q) and movement (M).

(**K**) Change in average V_m (Δ V_m) during movement in the absence (Ctrl, n = 8) and presence of noradrenergic receptor antagonists (green symbols, n = 5).

Table S1. Intrinsic properties of $M1_{FL}$ $L5B_{supp}$ and $L5B_{enh}$ pyramidal neurons recorded during quiet wakefulness. Related to Figure 1.

	$L5B_{supp}$			n	$L5B_{enh}$			n	p-value
**Firing rate (Hz)**	6.3	±	3.9	17	5.7	±	3.8	24	0.587
**Mean Vm (mV)**	-49.0	±	4.7	17	-52.0	±	5.3	24	0.061
**Std. Vm (mV)**	3.5	±	0.8	17	4.1	±	1.1	24	0.375
**C (pF)**	85	±	17	5	76	±	16	5	0.217
**Input Resistance (MΩ)**	85.3	±	23.7	5	96.4	±	17.5	5	0.487
**Membrane time constant (ms)**	8.6	±	2.0	5	7.8	±	1.1	5	0.625
**Spike threshold (mV)**	-39.1	±	5.2	17	-40.6	±	4.0	24	0.390
**Spike FWHM (ms)**	1.1	±	0.4	17	1.0	±	0.4	24	0.420
**Spike dV/dt peak:trough**	3.0	±	0.8	17	3.0	±	0.6	24	0.989
**Accommodation**	0.6	±	0.3	4	0.8	±	0.4	7	0.297
**Sag (%)**	3.8	±	2.4	6	4.3	±	4.4	15	0.974

Table S2. Intrinsic properties of identified $M1_{FL}$ PT-type and IT-type projection neurons recorded during quiet wakefulness. Related to Figure 1.

	IT-type			n	PT-type			n	p-value
**Firing rate (Hz)**	4.0	±	2.9	12	6.7	±	4.7	6	0.125
**Mean Vm (mV)**	-48.6	±	2.6	12	-47.6	±	7.1	6	0.553
**Std. Vm (mV)**	4.2	±	1.1	12	3.3	±	0.8	6	0.151
**Spike threshold (mV)**	-37.1	±	4.1	12	-39.7	±	7.1	6	0.094
**Spike FWHM (ms)**	1.2	±	0.3	12	1.1	±	0.4	6	0.250
**Spike dV/dt peak:trough**	3.3	±	0.6	12	2.8	±	0.5	6	0.068
**Accommodation**	0.6	±	0.3	5	0.9	±	0.6	3	0.393
**Sag (%)**	3.5	±	2.4	6	7.2	±	6.0	5	0.247

Supplemental Experimental Procedures

Mapping M1$_{FL}$ using intracortical microstimulation

The forelimb region of the primary motor cortex (M1$_{FL}$) was mapped using intracortical microstimulation (ICMS) in 7 male mice under 0.5 % isoflurane anesthesia. Glass micropipettes (1-2 MΩ) filled with extracellular solution were targeted to layer 5 (620 – 800 μm) and cathodal pulse trains (50 ms train duration, 30-350 μA, 300 μs pulse duration, 333 Hz) (A.M.P.I. Iso-flex) were applied through the stimulating electrode and slowly increased until contralateral forelimb muscle twitches could be observed. Stimulation sites were spaced approximately 250 μm apart and 24 ICMS sites were used to map M1$_{FL}$. Movements were visually scored and movement probability maps were generated for forelimb, hindlimb, wrist/digits and trunk.

Motion index and motor pattern discrimination

We defined a region-of-interest (ROI) covering the contralateral (left) forelimb and calculated the motion index (MI) for each successive frame as $\mathrm{MI}_f = \Sigma_{i=1}^{N}(c_{f+1,i} - c_{f,i})^2$, where $c_{f,i}$ is the grayscale level of the pixel i of the ROI in the frame f (see e.g. Figure 1A). We defined grooming as periods with no locomotion and MI> θ_m, where threshold θ_m was determined by visual inspection. Periods shorter than 2 s were not included in the analysis.

The position of the left paw was semi-automatically tracked offline using motion capture software (Blender ver. 2.7.1). In MATLAB horizontal and vertical pixel coordinates of the paw position during each 16.6 ms frame (paw accelerations > 5 pixels.s^{-2}, sustained at > 3 motions.s^{-1}) were re-centered around the average paw rest position for the 1 s preceding the onset of the running bout. To extract repeatable phases of each step during running, movements between local minima in the 1st principal component of the horizontal and vertical motion were classified as individual steps. Horizontal and vertical coordinates between each step onset were then extracted. To quantify similarity of paw movement between individual steps, total path lengths of 2-dimensional paw trajectories and area of enclosed space were calculated for each individual stepping motion.

In vivo electrophysiology and pharmacology

To confirm changes in behavioral state – quiet wakefulness to movement – local field potentials (LFPs) were recorded in M1$_{FL}$ L5B of head-restrained mice. Low-resistance ($\sim$1-2 MΩ) glass micropipettes were filled with external solution and LFP signals were high-pass filtered at 1 Hz.

For pre-application of noradrenergic receptor antagonists (Figure 5), recordings of L5B pyramidal neurons were performed > 40 minutes after drug application. For long-term recordings (whole-cell or cell-attached, Figure 6) the pre-application phase (5-10 mins) involved recording L5B pyramidal neuron activity in the presence of external solution before replacing with a mixture of noradrenergic receptor antagonists. Recordings

continued for > 30 minutes to ensure equilibrium antagonist concentration across the entire somatodendritic structure of L5B pyramidal neurons. Due to the time-dependent drift of V_m during long whole-cell recordings (pre: -51.6 mV, after 40 min: -39.7 mV, n=5) the mean membrane potential was corrected by -12 mV in Figure 6B.

Behavioral testing

To assess motor coordination following local noradrenergic receptor blockade in $M1_{FL}$, mice were head-restrained and habituated (20-45 min, over 2-6 days) to walk / run on a cylindrical runged treadmill. Craniotomies were performed above the right $M1_{FL}$ (see Animals and surgery) and after 1.5 h recovery, mice were head-restrained and 2-min video sequences were captured using a digital video camera (25 fps) before and 60 minutes after drug application (1 mM prazosin, yohimbine and propranolol; see *In vivo* electrophysiology and pharmacology in main Experimental Procedures). For sham controls external saline solution was added to the open craniotomy. Left (contralateral) and right (ipsilateral) forepaw placements were scored independently by two researchers and classified as precise forepaw placements or misplacements (i.e. incomplete forepaw placement, single/double digit contact, altered wrist angle, forepaw slips – in front or behind rungs; see Figure 6E).

Drug diffusion kinetics

To estimate the drug diffusion dynamics of topically applied drugs we applied phenol red (10-50 mM, in external solution) to the open craniotomy above $M1_{FL}$ for 60 minutes before slicing the brain in 1 mm sections. Brightfield images were converted to grayscale and pixel intensities were measured along an axis perpendicular to the brain surface. Tissue background pixel intensity was subtracted and pixel intensity was normalized to peak dye concentration at the brain surface.

Retrograde tracing of projection targets

Projection targets of individual neurons were identified by injecting fluorescent retrograde microspheres (RetroBeads, Lumafluor, USA) into the ipsilateral pons and contralateral hemisphere (dorsal striatum, corpus callosum and overlying cortex). Stereotaxic coor- dinates and volumes for pons injections were: 0.4 mm lateral, 3.9 and 4.1 mm posterior to bregma, 0.2 (75 nl), 0.4 (75 nl) and 0.6 mm (50 nl) from the ventral surface of the brain, green beads. For the dorsal striatum: 2.0 mm lateral, 0.2 and 0.6 mm anterior to bregma and 2.7, 2.5 and 2.2 mm ventral to the pial surface, 6x 50 nl, red beads. 2-3 days after injection mice were transcardially perfused with 4 % paraformaldehyde and brains processed to identify injection sites and retrograde staining in cortex.

Reconstruction of neuronal morphology and multi-labeling immunohistochemistry

After each recording, deeply anesthetized mice were transcardially perfused with 4 % paraformaldehyde. Mouse brains were post-fixed overnight and coronal sections (60 or 100 μm) of M1 and tracer injection sites were cut using a vibrating microtome. For morphological reconstructions, sections were incubated in streptavidin AlexaFluor-488

(1:1000, Molecular Probes) in 0.1 M phosphate buffered saline (PBS) containing 0.5 % Triton X-100 then mounted (Vectashield, VectorLabs) and imaged using a Zeiss LSM 510 Meta confocal microscope (20x objective). Morphological reconstructions were generated from 60 μm z-stacks using NeuronJ (ImageJ plugin). To identify projection targets of individually recorded neurons, sections were further processed by heat-mediated antigen retrieval in 10 mM sodium citrate buffer (pH 6.0) for 3 hrs at 80 °C. Sections were incubated in blocking solution (0.01 M PBS, 10 % normal goat serum (vol/vol), 0.5 % Triton X-100 (vol/vol) at 22 °C for 2 hrs and incubated overnight at 22 °C in a primary antibody mixture containing mouse monoclonal anti-SATB2 (1:200, Cat. No. ab51502, Abcam) and rat monoclonal anti-CTIP2 (1:1000, Cat. No. ab18465, Abcam) dissolved in carrier solution (0.01 M PBS, 1 % goat serum, 0.5 % Triton X-100). Slices were then incubated overnight at 22 °C in a secondary antibody mixture containing AlexaFluor-568 goat anti-mouse (1:750, Molecular Probes) and AlexaFluor-647 goat anti-rat (1:750, Molecular Probes) dissolved in carrier solution (0.01 M PBS, 1 % goat serum, 0.5 % Triton X-100), mounted and imaged using a Nikon A1R FLIM confocal microscope (Nikon, Europe). A sequential imaging protocol was used to minimize cross talk between fluorophores. Images were analyzed offline using ImageJ.

To validate the selective expression of CTIP2 and SATB2 in PT and IT neurons respectively, we combined retrograde tracing with post hoc CTIP2/SATB2 immunohistochemistry (see Figure S3 and Figure S4). Quantification of CTIP2 or SATB2 expression in individual, retrogradely identified L5B pyramidal neurons confirmed that all retrogradely labeled PT neurons were CTIP2 immunopositive, with only a small proportion of neurons co-expressing CTIP2 and SATB2 (9.7 %). Similarly, all retrogradely identified IT neurons expressed SATB2, with a minority expressing both molecular markers (2.7 %). For a subset of neurons we employed a dual strategy combining post hoc immunohistochemistry and morphological analysis to confirm the mixed PT- and IT-type projection-class identity of L5B$_{enh}$ and L5B$_{supp}$ subpopulations.

To label noradrenergic fibers in M1, coronal sections (60 μm) were first incubated in blocking solution (0.01 M PBS, 10 % normal goat serum (vol/vol), 0.5 % Triton X-100 (vol/vol) at 22 °C for 2 hrs, then incubated in rabbit polyclonal anti-noradrenaline transporter antibody (anti-NAT; 1:2000, Cat. No. 260003, Synaptic Systems) and then in AlexaFluor-488 goat anti-rabbit antibody (1:750, Molecular Probes). The primary and secondary antibodies were diluted in carrier solution (0.01 M PBS, 1 % goat serum, 0.5 % Triton X-100) and incubated overnight at 22 °C. Slices were mounted and z-stack images were acquired using a Nikon A1R FLIM confocal microscope (40x objective) (Nikon, Europe).

Data analysis

Intrinsic biophysical properties

Spike threshold was defined as the maximum of the second derivative of the voltage trace $V_m(t)$. Spike FWHM is the width of the spike measured at half of the distance between the threshold and the peak. dV/dt peak:trough is the ratio of the peak to the trough of the voltage derivative. Spike accommodation was measured by injecting 60 depolarizing current steps (0.5 s long, 100-500 pA scaled according to input resistance). The accommodation index was defined as $(ISI_2 - ISI_1)/ISI_1$, where ISI_1 and ISI_2 are the first and second inter-spike intervals at the beginning of each current step. Membrane potential sag was measured using hyperpolarizing current steps (250 ms, –100-500 pA scaled according to input resistance) and calculated as the percentage difference between the peak amplitude of the initial response (0-0.1 s) relative to the peak amplitude of the steady state response (0.15-0.25 s).

Membrane potential dynamics

Analysis of the subthreshold V_m dynamics was performed after clipping spikes from threshold to 3 ms after the peak (changing the clipping window to 10 ms before and 20 ms after each spike did not alter the results). The average standard deviation of the voltage (V_m SD) was calculated as the average over non-overlapping periods of 1 s.

We measured the power-spectra of V_m with 75 % overlapping Bartlett 1 s windows. The mean population spectrum was the average of the single cell spectra. The time-frequency spectrograms of the subthreshold voltage were computed with a continuous Morlet wavelet transform.

Detection of compound synaptic events

We could faithfully detect compound EPSPs occurring in a time window (5 ms) shorter than the average membrane time constant (9.0 $\pm$ 2.6 ms) and with a detection threshold of 1 mV. Events that occurred within $\pm$ 10 ms of a spike were discarded. To assess the reliability of our event detection algorithm we simulated noisy membrane potential traces (100 s in duration using Ornstein-Uhlenbeck processes) and manually added events of known amplitude at frequencies ranging from 5-100 Hz. For each frequency range 30 simulations were run and the mean detection efficiency computed (range 90-95 %) across the entire frequency range.

Effective input resistance and membrane time constant

We defined the effective input resistance as $R_{\text{eff}} = 1/G_{\text{tot}}$, the inverse of the total conductance $G_{\text{tot}} = G_L + \langle G_e \rangle + \langle G_i \rangle$, sum of the leak conductance G_L and the average synaptic excitatory and inhibitory conductances $\langle G_e \rangle$ and $\langle G_i \rangle$. For estimating the non-synaptic leak we obtained the typical R_{input} ($\sim$180 MΩ, G_{leak} = 1/R_{input}) from published *in vitro* cortical L5 pyramidal neuron data (Lefort et al., 2009; Sheets et al., 2011). The estimation of excitatory and inhibitory conductances are based on a population mean and the

distribution of R$_{input}$ typically displayed a standard deviation of $\sim$20 MΩ. Since we recorded $\sim$20 cells per group, the error on the estimation of the mean R$_{input}$ is $\sim 20/\sqrt{20}$) = 5 MΩ. If we estimate G$_e$ and G$_i$ by varying R$_{input}$ by $\pm$5 MΩ, the results will differ by < 5 %. Even in the extreme case where R$_{input}$ = 160 MΩ ($\sim$20 MΩ change), our estimation of G$_e$ and G$_i$ only varied by $\sim$10-15 %. The effective time constant is $\tau_{\text{eff}} = R_{\text{eff}}C$, where C is the capacitance. R_{eff} and τ_{eff} were estimated from the current injection experiment, calculating the average response to subthreshold injections with more than 20 pulses. We computed the complex impedance $Z(f) = V(f)/I(f)$, where $V(f)$ and $I(f)$ are respectively the Fourier transform of average voltage response and injected current, $|Z(f)| = R_{\text{eff}}/\sqrt{1+(2\pi f \tau_{\text{eff}})^2}$, to estimate R_{eff} and τ_{eff} (Figure S2).

Current injection and spiking probability

To measure the firing probability in response to injected EPSCs, we injected exponentially decaying EPSCs $I(t) = ae^{-t/\tau}$ of variable size a and fixed decay $\tau_s = 5$ ms. EPSCs of different amplitude (in total 80 for each amplitude, range 40-1200 pA) were injected in random order with 100 ± 10 ms intervals. To calculate the relationship between EPSP and EPSC size, we injected a series of 30 EPSCs and measured the average peak EPSP.

To estimate the probability p_i of producing an extra spike, we counted the number of spikes in the 10 ms following and in the 10 ms preceding the EPSC injection. We calculated p_i as the fraction of EPSCs injections of size a_i that produce an extra spike. The probability function $P(w)$ that an EPSP of size w causes a spike is $P(w) = \int_{\theta_r - w}^{\theta_r} p(V)dV$, defining the membrane potential distribution $p(V)$ and the spike threshold θ_r relative to the mean V_m. If we assume a Gaussian distribution for $p(V)$, with zero mean and standard deviation σ, one has $P(w) = \frac{1}{2}[\text{erf}(\theta_r/\sigma\sqrt{2}) - \text{erf}((\theta_r - w)/\sigma\sqrt{2}))]$, where erf is the error function. For each neuron i, we fitted $P_{quiet}(w)$ and $P_{mov}(w)$. For each neuron we calculated the Δ spike probability $S(w) = P_{mov}(w)/P_{quiet}(w)$ and its average between 0-8 mV.

Supplemental References

Lefort, S., Tomm, C., Sarria, J.-C. F. and Petersen, C. C. H. (2009). The excitatory neuronal network of the C2 barrel column in mouse primary somatosensory cortex. Neuron *61*, 301–16.

Sheets, P. L., Suter, B. A., Kiritani, T., Chan, C. S., Surmeier, D. J. and Shepherd, G. M. G. (2011). Corticospinal-specific HCN expression in mouse motor cortex: I(h)-dependent synaptic integration as a candidate microcircuit mechanism involved in motor control. J Neurophysiol *106*, 2216–31.
