# Supplementary material for: Cellular Mechanisms Underlying Behavioral State-Dependent Bidirectional Modulation of Motor Cortex Output
Source: Cell Rep. 2015 May 14;11(8):1319–30. doi: 10.1016/j.celrep.2015.04.042 (PMC4451462; doi:10.1016/j.celrep.2015.04.042)
Supplement: Document S2. Article plus Supplemental Information [file mmc2.pdf]

Cell Reports

Article

Cellular Mechanisms Underlying Behavioral State-Dependent Bidirectional Modulation of Motor Cortex Output

Graphical Abstract

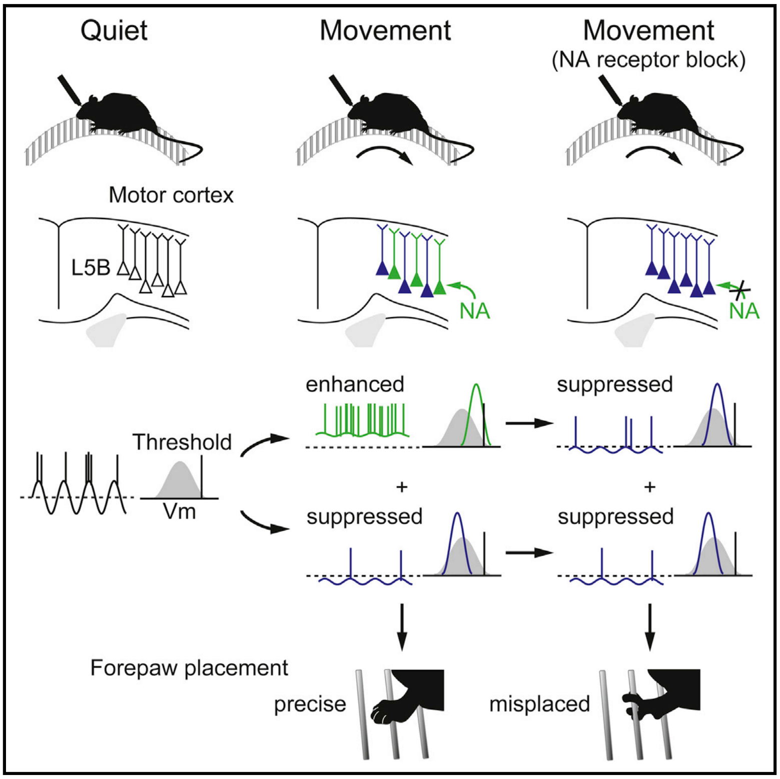

Highlights

- During movement, reduced V_m variance lowers spike probability in L5B output neurons
- Noradrenaline mediates a tonic depolarization in $L5B_{enh}$ neurons during movement
- Noradrenaline selectively enhances signal-to-baseline ratio of $L5B_{enh}$ neurons
- Blocking noradrenaline receptors in M1 reduces contralateral forepaw motor coordination

Authors

Julia Schiemann, Paolo Puggioni, ..., Mark C.W. van Rossum, Ian Duguid

Correspondence

ian.duguid@ed.ac.uk

In Brief

Schiemann et al. show that, in mouse motor cortex, layer 5B pyramidal neuron firing rates are suppressed or enhanced during movement due to a global reduction in membrane potential variability and coincident noradrenaline-mediated depolarization in a subpopulation of neurons, respectively. Blocking noradrenergic input to M1 impairs motor coordination.

Schiemann et al., 2015, Cell Reports *11*, 1319–1330
May 26, 2015 ©2015 The Authors
http://dx.doi.org/10.1016/j.celrep.2015.04.042

Cell Reports
Article

OPEN ACCESS
CellPress

Cellular Mechanisms Underlying Behavioral State-Dependent Bidirectional Modulation of Motor Cortex Output

Julia Schiemann,[1,4] Paolo Puggioni,[1,2,3,4] Joshua Dacre,[1] Miha Pelko,[2,3] Aleksander Domanski,[1] Mark C.W. van Rossum,[2] and Ian Duguid[1,*]
[1]Centre for Integrative Physiology and Patrick Wild Centre, University of Edinburgh, Hugh Robson Building, George Square, Edinburgh EH8 9XD, UK
[2]Institute for Adaptive and Neural Computation, School of Informatics, University of Edinburgh, Edinburgh EH8 9AB, UK
[3]Neuroinformatics Doctoral Training Centre, School of Informatics, University of Edinburgh, Edinburgh EH8 9AB, UK
[4]Co-first author
*Correspondence: ian.duguid@ed.ac.uk
http://dx.doi.org/10.1016/j.celrep.2015.04.042
This is an open access article under the CC BY-NC-ND license (http://creativecommons.org/licenses/by-nc-nd/4.0/).

SUMMARY

Neuronal activity in primary motor cortex (M1) correlates with behavioral state, but the cellular mechanisms underpinning behavioral state-dependent modulation of M1 output remain largely unresolved. Here, we performed in vivo patch-clamp recordings from layer 5B (L5B) pyramidal neurons in awake mice during quiet wakefulness and self-paced, voluntary movement. We show that L5B output neurons display bidirectional (i.e., enhanced or suppressed) firing rate changes during movement, mediated via two opposing subthreshold mechanisms: (1) a global decrease in membrane potential variability that reduced L5B firing rates ($L5B_{suppressed}$ neurons), and (2) a coincident noradrenaline-mediated increase in excitatory drive to a subpopulation of L5B neurons ($L5B_{enhanced}$ neurons) that elevated firing rates. Blocking noradrenergic receptors in forelimb M1 abolished the bidirectional modulation of M1 output during movement and selectively impaired contralateral forelimb motor coordination. Together, our results provide a mechanism for how noradrenergic neuromodulation and network-driven input changes bidirectionally modulate M1 output during motor behavior.

INTRODUCTION

Neuronal activity in layer 5 (L5) of primary motor cortex (M1) correlates with rhythmic voluntary movements (Armstrong and Drew, 1984a, 1984b). During walking or running, pyramidal neurons display changes in firing rate that reflect periods of coordinated muscle activity (Armstrong and Drew, 1984a; Beloozerova et al., 2003). Although spontaneous locomotor activity can be controlled by central pattern generators (CPGs) in the spinal cord (Forssberg et al., 1980; Grillner, 1981; Grillner and Zangger, 1979), descending motor commands from M1 are integrated with ongoing rhythmic spinal cord signals and sensory input from the periphery to initiate, adjust, and maintain locomotor function (Armstrong and Drew, 1984a; Beloozerova et al., 2003; Orlovsky, 1972; Ueno and Yamashita, 2011). In lower mammals, such as cats, rabbits, and mice, discrete subpopulations of L5 output neurons display enhanced or suppressed (i.e., bidirectional) firing rate changes during locomotion (Armstrong and Drew, 1984a; Beloozerova et al., 2003; Costa et al., 2004). In rodents, these changes can be either abrupt, sustained changes—so-called on-off responses—or gradual frequency changes linked to the velocity of running (Costa et al., 2004). Although we are now beginning to understand how patterns of motor cortex activity relate to changes in behavioral state in rodents (i.e., quiet wakefulness to movement), the cellular mechanisms underpinning bidirectional modulation of M1 output during self-paced movement remain largely unresolved.

Several mechanisms could underlie the bidirectional modulation of M1 output, such as a change in cortical state-dependent network-driven input structure, intracortical or long-range excitatory input, and/or neuromodulation. During quiet wakefulness or slow-wave sleep, cortical networks remain in a synchronized state that consists of slow, large-amplitude oscillations in neuronal population activity (Cowan and Wilson, 1994; Steriade et al., 1993c). During active behavior, cortical networks enter an activated state characterized by a reduction in slow oscillations and, in some cases, an increase in higher frequency activity (Steriade et al., 1993b; Timofeev et al., 2001). This change profoundly alters the subthreshold V_m dynamics and spike output patterns of cortical pyramidal neurons (Castro-Alamancos, 2004; Castro-Alamancos and Oldford, 2002; Constantinople and Bruno, 2011; Crochet and Petersen, 2006). Thalamic activation promotes the cortical awake state and direct depolarization of superficial and deep-layer pyramidal neurons (Castro-Alamancos and Connors, 1996; Castro-Alamancos and Oldford, 2002; Constantinople and Bruno, 2013; Hirata and Castro-Alamancos, 2010; Poulet et al., 2012), suggesting input from the thalamus could contribute to changes in M1 output during motor

CrossMark

OPEN ACCESS
CellPress

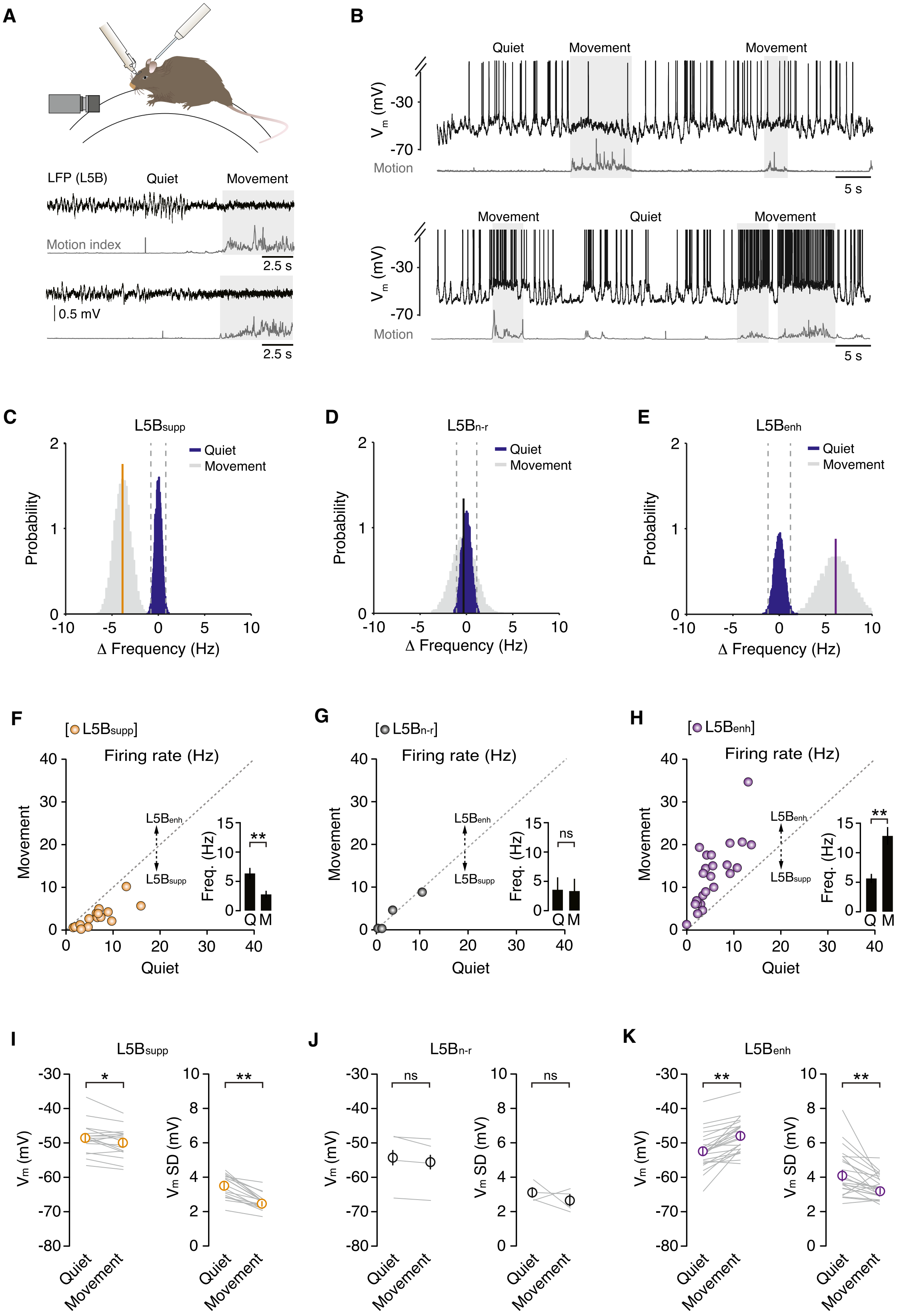

(legend on next page)

behavior. Anatomical and functional connectivity mapping have shown the presence of a strong top-down laminar organization of local excitatory microcircuits in M1, with feedforward projections from layer 2/3 (L2/3) targeting multiple classes of projection neurons in L5 (Kaneko et al., 1994; Weiler et al., 2008). Given that L2/3 pyramidal neurons can display dense clustered activity during head-restrained locomotion in mice (Dombeck et al., 2009), changes in descending excitation from upper-layer pyramidal neurons could be a mechanism for generating bidirectional modulation of M1 output. Alternatively, neuromodulators are important for cortical processing, with noradrenaline and acetylcholine release being associated with changes in arousal, vigilance, and behavioral state (Berridge and Waterhouse, 2003; Carter et al., 2010; Castro-Alamancos and Gulati, 2014; Constantinople and Bruno, 2011; Eggermann et al., 2014; Fu et al., 2014; Polack et al., 2013; Steriade et al., 1993a). Thus, how local, long-range, and neuromodulatory inputs regulate L5 pyramidal neuron V_m dynamics during changes in behavioral state remains to be fully established.

Here we combined in vivo patch-clamp recordings in awake mice with selective pharmacology to investigate the cellular mechanisms underpinning behavioral state-dependent modulation of motor cortex output. We found that changing behavioral state, from quiet wakefulness to movement, bidirectionally modulated (i.e., enhanced or suppressed) M1 output via two opposing subthreshold mechanisms: (1) a global decrease in network-driven, slow, large-amplitude V_m fluctuations, which reduced V_m variability, spike probability, and firing rates in L5B pyramidal neurons ($L5B_{suppressed}$ neurons); and (2) a coincident increase in excitatory drive to a subpopulation of L5B neurons ($L5B_{enhanced}$), which depolarized mean V_m and enhanced firing rates. We found that the movement-related tonic depolarization in $L5B_{enh}$ neurons was dependent on the interplay between ascending motor thalamic input, which maintained V_m near threshold, and noradrenergic input from the locus coeruleus (LC). The behavioral state-dependent release of noradrenaline increased the signal-to-baseline ratio (SBR) for movement-evoked responses in $L5B_{enh}$ neurons. Selectively blocking noradrenergic input in the forelimb region of M1 significantly reduced motor coordination in the contralateral forelimb during motor behavior. Thus, our findings provide a mechanism for how noradrenergic neuromodulation and network-driven input changes bidirectionally modulate M1 output during self-paced voluntary movement.

RESULTS

Membrane Potential Dynamics of L5B Pyramidal Neurons during Self-Paced, Voluntary Movement

To investigate the cellular mechanisms underpinning behavioral state-dependent modulation of M1 output, we obtained whole-cell patch-clamp recordings from L5B pyramidal neurons (forelimb motor cortex, 620–880 μm from the pial surface; see Experimental Procedures; n = 45 neurons) during quiet wakefulness and self-paced, voluntary movements (i.e., walking, running, or grooming on a single-axis, cylindrical treadmill; Figure 1A). During periods of quiet wakefulness, all L5B pyramidal neurons displayed large-amplitude V_m fluctuations (V_m SD = 3.8 ± 0.2 mV) and a relatively depolarized average V_m (V_m = −51.1 ± 0.8 mV). The interplay among mean V_m, distance from threshold, and V_m variability resulted in moderate basal firing rates (5.7 ± 0.6 Hz, range: 0.0–15.9 Hz; Figures 1B–1K and S1).

During switches in behavioral state (i.e., quiet wakefulness to movement), characterized by a low-amplitude, high-frequency local field potential signal in L5B (Figure 1A), the vast majority of L5B pyramidal neurons (~90%) displayed significant modulation of their basal firing rates. To functionally classify individual neurons, we compared the variability in quiet wakefulness firing rate with the average firing rate during self-paced movement (see Experimental Procedures). If the average movement-related firing rate was lower than the first percentile of the distribution of firing rate changes during quiet wakefulness, neurons were classified as suppressed ($L5B_{supp}$, n = 17; Figures 1C and 1F; Table S1), while neurons that displayed an average movement-related firing rate above the 99th percentile were classified as enhanced ($L5B_{enh}$, n = 24; Figures 1E and 1H; Table S1). A small proportion of L5B neurons (n = 4/45) did not significantly change their firing rates during movement and were classified as non-responding neurons ($L5B_{n\text{-}r}$; Figures 1D and 1G). The proportion of L5 pyramidal neurons in which spike frequency decreased (38%), increased (53%), or did not change (9%) during movement was consistent with previous reports (Beloozerova et al.,

Figure 1. Membrane Potential Dynamics of L5B Pyramidal Neurons in M1 during Self-Paced, Voluntary Movement

(A) Patch-clamp recording configuration in head-fixed mice mounted on a single-axis, cylindrical treadmill. Local field potential (LFP) recordings (black traces, n = 2 mice) of L5B activity and moderate speed (60 frames/s) digital imaging were used to confirm changes in behavioral state (quiet wakefulness to movement) and to calculate motion index (gray traces).

(B) Representative voltage traces from two L5B pyramidal neurons that displayed either a decrease (top) or increase (bottom) in firing rate during movement (light gray shading). The motion index (dark gray) defines the magnitude and duration of each forelimb movement. In this figure and all subsequent figures, action potentials have been truncated to highlight subthreshold V_m changes during movement.

(C–E) Representative change in firing rate probability distributions during quiet wakefulness (blue) and movement (gray) in $L5B_{suppressed}$ (C), $L5B_{non\text{-}responding}$ (D), and $L5B_{enhanced}$ (E) neurons. Gray dotted lines represent the 1st (left) and 99th (right) percentiles. Solid colored lines represent the average firing rate change in $L5B_{suppressed}$ (yellow), $L5B_{non\text{-}responding}$ (black), and $L5B_{enhanced}$ (purple) neurons during movement.

(F–H) Average firing rate during quiet wakefulness and movement in $L5B_{suppressed}$ (F, n = 17), $L5B_{non\text{-}responding}$ (G, n = 4), and $L5B_{enhanced}$ (H, n = 24) neurons. Filled circles represent data from individual neurons. Insets depict the average firing rate ± SEM during quiet wakefulness (Q) and movement (M). **p < 0.01; ns, non-significant.

(I–K) Average V_m (left-hand plot) and V_m SD (right-hand plot) in $L5B_{suppressed}$ (I, n = 17), $L5B_{non\text{-}responding}$ (J, n = 4), and $L5B_{enhanced}$ (K, n = 24) neurons during quiet wakefulness and movement. Solid gray lines represent data from individual neurons while open symbols represent mean ± SEM. *p < 0.05, **p < 0.01; ns, non-significant.

See also Figures S1–S4 and Tables S1 and S2.

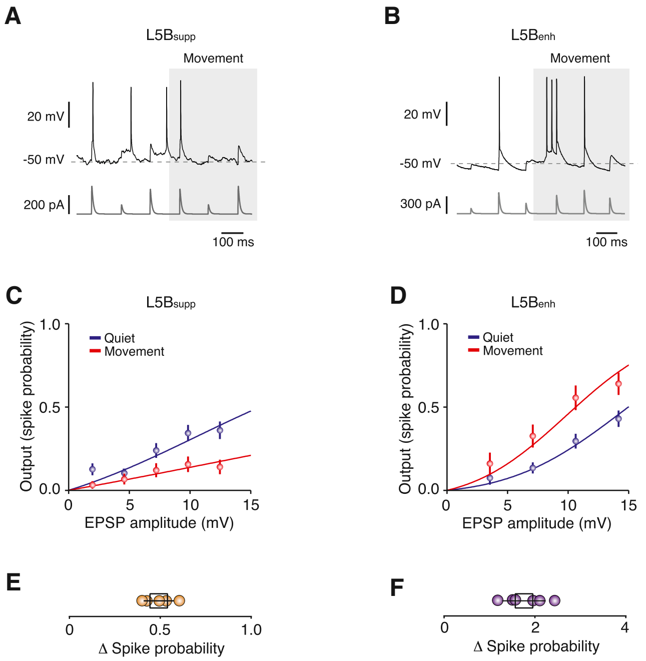

Figure 2. Input-Output Transformations in $L5B_{supp}$ and $L5B_{enh}$ Pyramidal Neurons during Movement

(A and B) Representative voltage traces (upper trace, black) during somatic EPSC-like current injections in vivo (lower trace, dark gray) in $L5B_{supp}$ (A) and $L5B_{enh}$ (B) pyramidal neurons during quiet wakefulness and movement (light gray shading).

(C and D) Input-output transformations in $L5B_{supp}$ (C, n = 5) and $L5B_{enh}$ (D, n = 6) neurons recorded in vivo during quiet wakefulness (blue) and movement (red). Symbols represent mean ± SD; solid lines are fits to a truncated error function.

(E and F) Mean change in spike probability for $L5B_{supp}$ (E, n = 5) and $L5B_{enh}$ (F, n = 6) neurons. Filled symbols represent data from individual neurons and black open squares represent mean ± SD.

See also Figure S2.

2003; Costa et al., 2004). Moreover, the functional classification of individual neurons remained consistent during repeated bouts of movement and was not dependent on the type of motor behavior being executed (Figure S1). To further demonstrate the coexistence of functionally distinct subpopulations of L5B pyramidal neurons in M1, we performed multiple recordings from the same mouse and identified $L5B_{enh}$, $L5B_{supp}$, and $L5B_{n\text{-}r}$ pyramidal neurons during the execution of similar forelimb movements (i.e., repeated forepaw swing/stance cycles, n = 8 recordings from three mice; $L5B_{supp}$/$L5B_{enh}$/$L5B_{n\text{-}r}$ ratio: 4:3:1, note similar ratio of functionally classified neurons when compared to the population data in Figure 1; Figure S1).

We next investigated the subthreshold mechanisms underpinning bidirectional modulation of M1 output. During movement, $L5B_{supp}$ neurons displayed ~1 mV hyperpolarization in mean V_m ($p = 2 \times 10^{-2}$) and reduced V_m variability (V_m SD quiet = 3.5 ± 0.2 mV, V_m SD movement = 2.5 ± 0.1 mV, $p = 3 \times 10^{-4}$), which lowered the probability of reaching threshold and reduced overall firing rates (quiet 6.4 ± 1.0 Hz, movement 2.8 ± 0.6 Hz, $p = 3 \times 10^{-4}$; Figures 1F and 1I). In $L5B_{enh}$ neurons, movement also reduced V_m variability (V_m SD quiet = 4.1 ± 0.3 mV, V_m SD movement = 3.2 ± 0.2 mV, $p = 2 \times 10^{-3}$), but this was counteracted by a depolarization in average V_m (quiet −52.4 ± 1.1 mV, movement −47.9 ± 1.0 mV, $p = 2 \times 10^{-6}$), which significantly increased spike probability and firing rates (quiet 5.7 ± 0.8 Hz, movement 12.9 ± 1.5 Hz, $p = 2 \times 10^{-5}$; Figures 1H and 1K). Moreover, movement-related firing rate changes strongly correlated with the level of V_m depolarization in individual $L5B_{enh}$ neurons (Figure S2). By contrast, V_m dynamics and firing rates of $L5B_{n\text{-}r}$ neurons were not affected by the transition from quiet wakefulness to movement (Figures 1G and 1J). Interestingly, the functional classification of L5B pyramidal neurons ($L5B_{supp}$ versus $L5B_{enh}$) was not dependent on their basic electrophysiological properties (Table S1) or the projection-class identity of individual neurons based on retrograde tracing and selective expression of the transcription factors CTIP2 (thick-tufted pyramidal tract [PT]-type neurons) and SATB2 (thin-tufted intratelencephalic [IT]-type neurons; Leone et al., 2008; Figures S3 and S4; Table S2). Together, our results suggest that movement-related modulation of $L5B_{enh}$ firing rates is primarily mediated by a tonic depolarization in V_m, while reduced firing rates in $L5B_{supp}$ neurons result from a moderate hyperpolarization and significant reduction in V_m variance.

L5B Input-Output Transformations during Voluntary Movement

Behavioral state-dependent changes in V_m dynamics can profoundly affect the integrative mode and output firing patterns of neocortical neurons. What effects do movement-related changes in V_m dynamics have on input-output transformations in M1 L5B pyramidal neurons? In principle, both changes in V_m SD and mean can influence the responsiveness and firing dynamics of a neuron (Chance et al., 2002; Hô and Destexhe, 2000). To test this, we performed current injection experiments (i.e., somatic injection of excitatory postsynaptic current [EPSC]-like waveforms) in a subset of $L5B_{supp}$ and $L5B_{enh}$ neurons in vivo (Figures 2A and 2B; Supplemental Experimental Procedures) and measured the spike probability during quiet wakefulness and voluntary movement. Although current injection at the soma disregards dendritic non-linearities, synaptic properties, and locations, it provides a robust measure to assess the relationship between synaptic conductances arriving at the soma and spike output probability during behavior. During movement $L5B_{supp}$ neurons, which experience a decrease in V_m SD with relatively little change in mean V_m (Figure 1), displayed a 2-fold reduction in spike probability (Δ Spike probability = 0.6 ± 0.1, n = 5; Figures 2C and 2E). By contrast, $L5B_{enh}$ neurons, which experience a decrease in V_m SD and an increase in mean V_m (Figure 1), displayed a 2-fold increase in spike probability (Δ Spike probability = 1.7 ± 0.4, n = 6; Figures 2D and 2F). Although both L5B subpopulations displayed moderate changes in input resistance during movement, they did not significantly differ from quiet wakefulness (n = 5 and 5, respectively, p = 0.32; Figure S2).

Changes in L5B Input Structure during Movement

To further investigate the mechanisms underpinning $L5B_{supp}$ and $L5B_{enh}$ neuron V_m dynamics, we explored changes in V_m spectral components before and after movement onset. During quiet wakefulness, we observed slow (1.5–4 Hz, δ frequency band), large-amplitude V_m fluctuations in all L5B pyramidal neurons

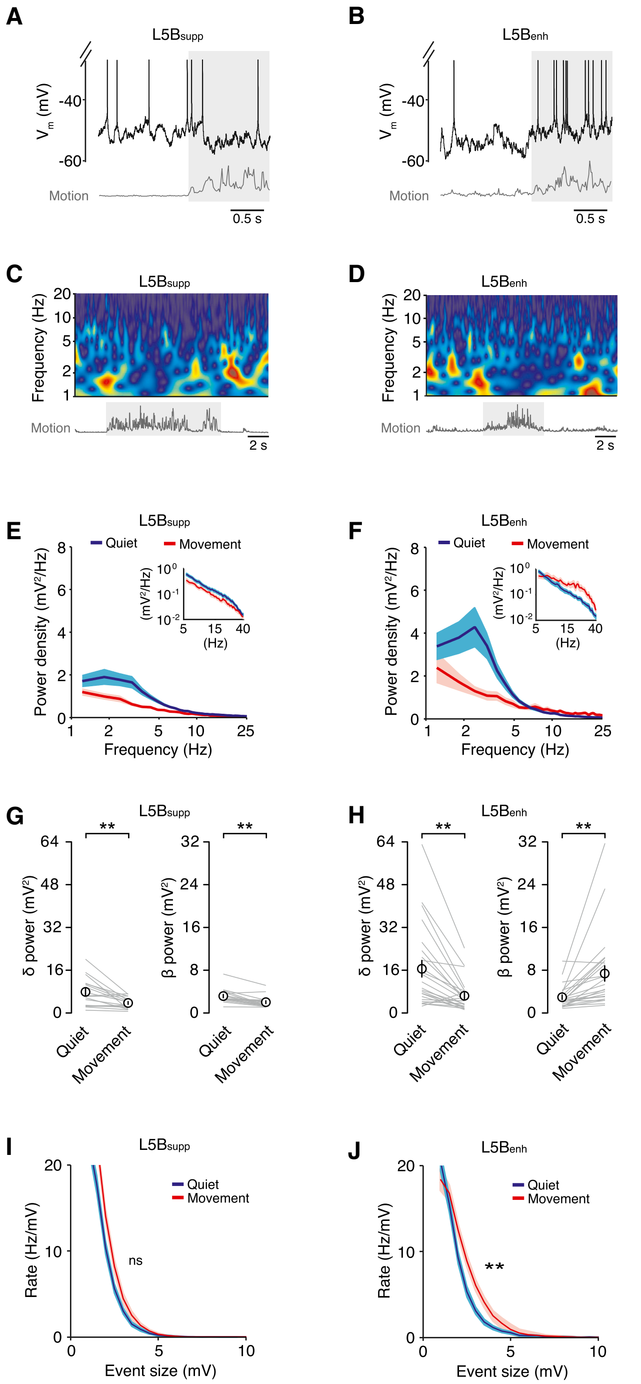

Figure 3. Movement Reduces Slow, Large-Amplitude V_m Fluctuations but Increases Excitatory Drive in $L5B_{enh}$ Neurons

(A and B) Representative high-time resolution voltage traces for $L5B_{supp}$ (A) and $L5B_{enh}$ (B) neurons during quiet wakefulness and movement (gray shading).

(C and D) Low-time resolution wavelet spectrograms for $L5B_{supp}$ (C) and $L5B_{enh}$ (D) neurons during quiet wakefulness and movement. Representative examples correspond to neurons shown in (A and B).

(E and F) Average V_m power density for $L5B_{supp}$ (E, n = 17) and $L5B_{enh}$ (F, n = 24) pyramidal neurons during quiet wakefulness (blue) and movement (red). Data represent mean ± SD. Insets show average V_m power density between 5 and 40 Hz.

(G and H) Average V_m power in δ (1.5–4 Hz) and β (12–30 Hz) frequency bands in $L5B_{supp}$ (G, n = 17) and $L5B_{enh}$ (H, n = 24) pyramidal neurons during quiet wakefulness and movement. Gray lines represent data from individual neurons and black symbols represent mean ± SEM. $^{**}p < 0.01$.

(I and J) Average rate density of compound synaptic events in $L5B_{supp}$ (I, n = 17) and $L5B_{enh}$ (J, n = 24) pyramidal neurons during quiet wakefulness (blue) and movement (red). Data represent mean ± SD. $^{**}p < 0.01$; ns, non-significant.

See also Figure S2.

(Figures 3A–3D), which were suppressed during movement ($L5B_{supp}$ quiet 7.8 ± 1.3 mV^2, movement 3.6 ± 0.5 mV^2, n = 17, $p = 2 \times 10^{-3}$; $L5B_{enh}$ quiet 16.4 ± 3.1 mV^2, movement 6.2 ± 1.2 mV^2, n = 24, $p = 1 \times 10^{-4}$; Figures 3A–3H). The reduction in δ power led to reduced V_m SD, which together with a moderate hyperpolarization (~1 mV) could account for the reduction in spike probability observed in $L5B_{supp}$ pyramidal neurons during movement (Figures 1 and S2). In $L5B_{enh}$ neurons, the suppression of slow V_m fluctuations was counteracted by an increase in power (12–30 Hz) in the β frequency band (12–30 Hz; quiet 3.0 ± 0.4 mV^2, movement 7.4 ± 1.4 mV^2, n = 24, $p = 3 \times 10^{-5}$; Figures 3F and 3H). The magnitude of increased β power displayed a strong positive correlation with the magnitude of V_m depolarization in individual $L5B_{enh}$ neurons (Figure S2), suggesting this could be the source of the increased excitatory drive.

To examine this further, we developed an event detection algorithm to estimate the level of excitatory input during quiet wakefulness and movement. Due to the high frequency of afferent input (estimated range: 5–15 kHz, data not shown), we were unable to isolate single excitatory postsynaptic potentials (EPSPs). However, we could reliably detect compound synaptic inputs (≥1 mV) occurring in a time window (5 ms) shorter than the average membrane time constant (8.2 ± 0.7 ms, n = 10; Figure S2). The detection threshold corresponded to twice the size of the average unitary synaptic response measured in L5 pyramidal neurons in vitro (Deuchars et al., 1994; Reyes and Sakmann, 1999). Events that occurred within ±10 ms of a spike were excluded from the analysis. During quiet wakefulness, we detected fast-rising compound EPSPs (range: 1–9.7 mV) with similar rates in both $L5B_{supp}$ and $L5B_{enh}$ pyramidal neurons (Figures 3I and 3J), indicating both subpopulations of neurons receive a comparable level of excitatory drive. During movement, the rate of compound events in $L5B_{supp}$ neurons was not affected (n = 17; Figure 3I), whereas $L5B_{enh}$ neurons displayed a significant increase in compound EPSP rate (n = 24; Figure 3J). Remarkably, we did not detect any compound events with amplitudes greater than 9.4 mV, even though neurons spent approximately 50% of the time >10 mV from threshold. Thus, $L5B_{enh}$ neurons appear to preferentially receive a net increase in excitatory drive during movement, which enhances the firing rate by depolarizing mean V_m and increasing spike probability.

Effects of Local and Long-Range Input to L5B Pyramidal Neurons during Self-Paced Movement

To investigate the source(s) of the increased excitatory drive to $L5B_{enh}$ neurons, we examined the activity of local and long-range inputs from L2/3 and motor thalamus, respectively.

OPEN ACCESS
CellPress

Previous studies have shown that M1 L2/3 neurons can be highly active during head-restrained locomotion on a spherical treadmill (Dombeck et al., 2009) and this descending excitation could potentially influence the activity of output neurons in L5 (Weiler et al., 2008). To test this possibility, we obtained whole-cell patch-clamp recordings from L2/3 pyramidal neurons (180–420 μm from the pial surface; Figure 4A). During quiet wakefulness, L2/3 neurons displayed relatively low firing rates, which were unaffected by the onset of movement (quiet 0.6 ± 0.3 Hz, movement 0.6 ± 0.4 Hz, n = 8; Figure 4B). Although the average V_m of L2/3 neurons depolarized by ~4 mV (Figure 4C), this was counteracted by a significant reduction in V_m SD (Figure 4D), which maintained baseline spike probability and firing rates. Given that our sample of L2/3 neurons displayed low spike rates during both quiet wakefulness and movement, this suggests that descending input from L2/3 is unlikely to be the primary source of the increased excitatory drive to $L5B_{enh}$ neurons in our experimental paradigm (see also Dombeck et al., 2009 and Discussion).

Given that thalamocortical neuron firing rates vary markedly depending on behavioral state and ventroanterior/ventrolateral (VA/VL) thalamic input to M1 displays bidirectional modulation during simple locomotion (Marlinski et al., 2012), we investigated the role of motor thalamus in regulating M1 output during movement. Blocking thalamic input by local infusion of the $GABA_A$ receptor agonist muscimol into the VA/VL complex (Experimental Procedures; Figure 4E) enhanced the amplitude of slow, large-amplitude V_m fluctuations (control V_m SD = 3.8 ± 0.2 mV versus thalamic inactivation SD = 5.1 ± 0.6 mV; n = 45 and n = 6, respectively; $p = 3.4 \times 10^{-2}$, Mann-Whitney U test) and produced a hyperpolarizing shift in average V_m (control $V_m = -51.1 \pm 0.8$ mV versus thalamic inactivation $V_m = -62.5 \pm 3.6$ mV; n = 45 and n = 6, respectively; $p = 8.0 \times 10^{-4}$), which significantly reduced the basal firing rate of L5B neurons compared to control conditions (control 5.7 ± 0.6 Hz versus thalamic inactivation 1.1 ± 0.5 Hz; n = 45 and n = 6, respectively; $p = 1.1 \times 10^{-3}$; Figures 4G and 4H; see also Figure 1). The hyperpolarization associated with thalamic inactivation increased the distance to threshold

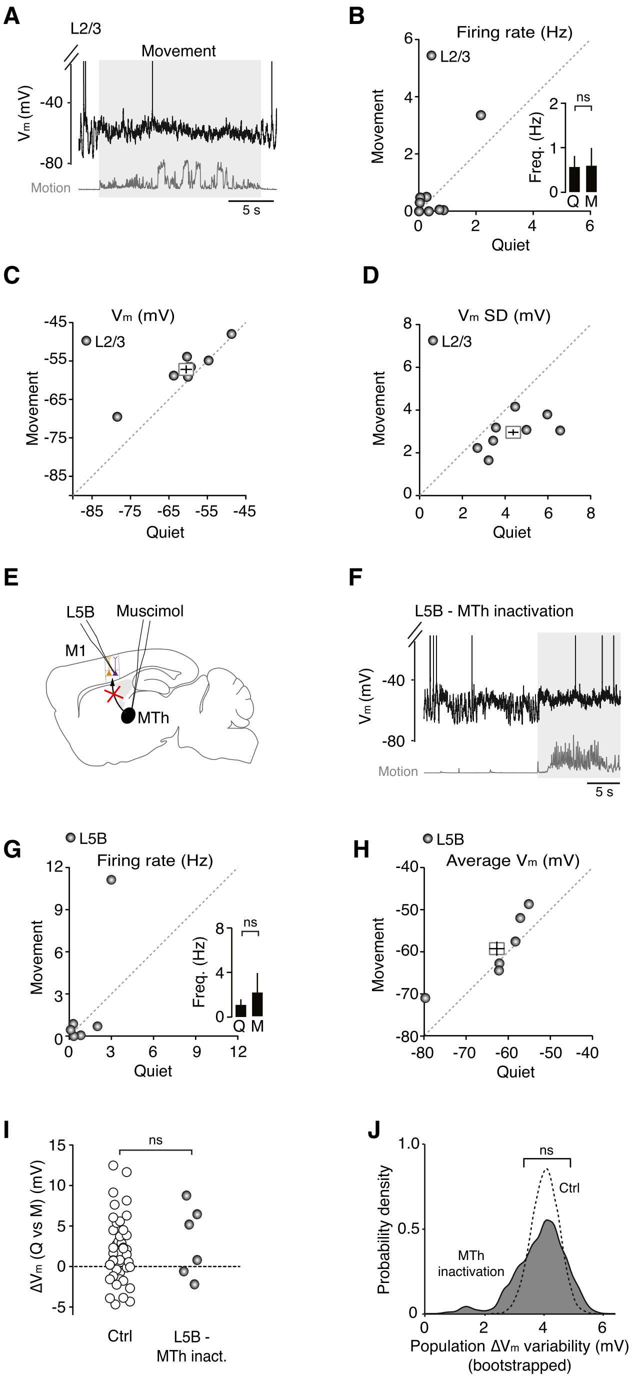

Figure 4. Effect of Descending L2/3 and Ascending Motor Thalamic Input on L5B Pyramidal Neuron V_m Dynamics during Quiet Wakefulness and Movement

(A) Representative voltage trace shows an L2/3 pyramidal neuron during quiet wakefulness and movement (gray shading).

(B–D) Average firing rate (B), mean V_m (C), and V_m SD (D) in L2/3 pyramidal neurons (gray symbols, n = 8) before and after movement. Filled circles represent data from individual neurons while square symbols represent mean ± SEM. Inset in (B) depicts average L2/3 pyramidal neuron firing rate during quiet wakefulness (Q) and movement (M). ns, non-significant.

(E) Schematic representation shows an L5B pyramidal neuron recording after inactivation of ipsilateral motor thalamus (MTh) by local perfusion of muscimol.

(F) Representative voltage trace showing an L5B pyramidal neuron after ipsilateral inactivation of motor thalamus.

(G and H) Average firing rate (G) and mean V_m (H) in L5B pyramidal neurons after motor thalamic inactivation (n = 6). Filled circles represent data from individual neurons while the square symbol in (H) represents the mean ± SEM. Inset in (G) depicts the average firing rate of L5B neurons during quiet wakefulness (Q) and movement (M). ns, non-significant.

(I) Change in average V_m (ΔV_m) during movement in the presence (Ctrl, open symbols, n = 41) and absence of motor thalamic input (gray symbols, n = 6). Control data (Ctrl) were taken from Figure 1 for comparison. Mann-Whitney U test; ns, non-significant.

(J) Probability density distributions of ΔV_m variability across the L5B pyramidal neuron population (Ctrl and MTh inact.), measured as the SD of the ΔV_m distributions shown in (I) (Population ΔV_m SD) using bootstrap analysis (10,000 bootstrap replicates). Black dashed line represents population ΔV_m variability distribution in control (Ctrl), and gray shading represents population ΔV_m variability distribution following motor thalamic inactivation. Control data (Ctrl) were taken from Figure 1 for comparison. F test; ns, non-significant.

See also Figure S5.

(data not shown) such that movement-related firing rate changes were abolished (Figure 4G), precluding the functional classification of $L5B_{supp}$ and $L5B_{enh}$ neurons. However, during movement 50% of L5B neurons (n = 3/6) still experienced a 5–10 mV depolarization in mean V_m (Figure 4I) and increased rate of compound EPSPs (Figure S5), similar to that observed in $L5B_{enh}$ neurons under control conditions (Figures 1 and 3). We analyzed this further by plotting the ΔV_m variability across the L5B pyramidal neuron population, measured as the SD of the ΔV_m distributions shown in Figure 4I (population ΔV_m SD), using bootstrap analysis (10,000 bootstrap replicates; Figure 4J). We found that motor thalamic inactivation did not affect the population ΔV_m variability in L5B pyramidal neurons compared to control (Figures 4I and 4J), suggesting input from the motor thalamus—either direct or indirect—is essential for maintaining L5B pyramidal neuron V_m near threshold, but is unlikely to be the main source of the increased excitatory drive.

Noradrenergic Neuromodulation Selectively Enhances Excitatory Drive and Signal-to-Baseline Ratio in $L5B_{enh}$ Neurons

Given that the movement-related increase in excitatory drive and tonic depolarization in $L5B_{enh}$ neurons could not be directly explained by increased excitation from L2/3 or motor thalamus, we next explored the role of noradrenergic neuromodulation, which has been shown to be important during changes in arousal, attention, and behavioral state (Berridge and Waterhouse, 2003; Carter et al., 2010; Castro-Alamancos and Gulati, 2014; Constantinople and Bruno, 2011; Polack et al., 2013). Selective immunohistochemical staining for the noradrenaline transporter (NAT), expressed exclusively in noradrenergic axons (Lorang et al., 1994), revealed dense axonal innervation of all layers in forelimb M1 (Figure 5A). To test the importance of noradrenergic input in regulating L5B pyramidal neuron V_m dynamics during movement, we topically applied $\alpha 1$, $\alpha 2$, and β noradrenergic receptor antagonists (1 mM prazosin, 1 mM yohimbine, and 1 mM propranolol, respectively) to the forelimb region of M1 (Figures 5B and 5C). The local infusion of noradrenergic receptor antagonists via the craniotomy selectively disrupted noradrenergic signaling in forelimb M1 (Figure S6), whereas direct manipulation of LC activity, via electrical stimulation or optogenetics, would have widespread effects across many brain areas and spinal cord circuits. Moreover, topical application was preferred due to the technical limitations of simultaneously pressure ejecting drugs at multiple sites along the entire somatodendritic length of L5B pyramidal neurons during intracellular recordings.

Blocking noradrenergic receptors reduced the mean V_m (control -51.1 ± 0.8 mV versus noradrenergic receptor blockade -56.6 ± 1.6 mV; $p = 4.0 \times 10^{-3}$) and quiet wakefulness firing rate of L5B pyramidal neurons (control 5.7 ± 0.6 Hz versus noradrenergic receptor blockade 1.9 ± 0.3 Hz; n = 45 and n = 16, respectively; $p < 1 \times 10^{-4}$; Figures 5D and 5E), and significantly reduced the proportion of L5B neurons that displayed enhanced firing rates during movement (control $L5B_{enh}$ 24/45 neurons [53.3%] versus noradrenergic receptor blockade $L5B_{enh}$ 2/16 neurons [12.5%]; $p < 1 \times 10^{-2}$; Figures 5F and 5G). The change in relative distribution of $L5B_{supp}$/$L5B_{enh}$ neurons could be explained in part by the moderate hyperpolarization in V_m and increased distance to threshold during movement (Figure S6). Although noradrenergic receptor blockade did not affect the mean population ΔV_m compared to control conditions, due to both distributions being centered around 0 mV (Figure 5H), we did observe a significant decrease in ΔV_m variability across the L5B pyramidal neuron population, measured as the SD of the ΔV_m distributions shown in Figure 5H (population ΔV_m SD) using bootstrap analysis (10,000 bootstrap replicates; Figure 5I). Consistent with the idea that noradrenergic signaling underpins a large proportion of the increased excitatory drive to $L5B_{enh}$ neurons during movement, blocking noradrenergic receptors also abolished the increase in V_m β-band power (Figure 5J) and rate of compound synaptic events associated with movement (Figures 5K and S5).

Given that pre-application of noradrenergic receptor antagonists precludes the prior identification of $L5B_{enh}$ neurons prior to receptor blockade, we also performed long-term (40- to 80-min) recordings from identified $L5B_{enh}$ neurons before (Figure 6A) and after (Figure 6B) receptor block. If noradrenergic neuromodulation underpins the V_m depolarization in $L5B_{enh}$ neurons during movement, then blocking noradrenergic receptors should have a disproportionately larger effect on movement-related firing rates compared to quiet firing rates. Accordingly, we found that receptor blockade resulted in a modest, time-dependent reduction in $L5B_{enh}$ basal firing rates and a strong suppression of movement-related firing (Figure 6C). The drug diffusion and time dependency of the antagonist effects in L5B were consistent with our dye diffusion mapping results (Figure S6). To assess the extent to which noradrenaline facilitates $L5B_{enh}$ output during movement, we examined the Signal-to-Baseline Ratio (SBR), defined as the ratio of the movement-related spike rate to the spike rate during quiet wakefulness. Blocking noradrenergic neurotransmission significantly reduced the SBR compared to control conditions (sham control SBR: 1.1 ± 0.1, noradrenergic receptor antagonist SBR: 0.3 ± 0.1; $p = 6 \times 10^{-3}$; n = 3 and 3, respectively; Figure 6D).

Since descending M1 output is essential for maintaining normal locomotor function (Armstrong and Drew, 1984a; Beloozerova et al., 2003; Orlovsky, 1972; Ueno and Yamashita, 2011), we investigated whether there was a behavioral correlate of reduced M1 output during noradrenergic receptor blockade by conducting a series of behavioral experiments using head-restrained mice habituated to walk/run on a cylindrical runged treadmill (Figure 6E). This experimental paradigm facilitates the analysis of precise forepaw placements during locomotion, which was not possible on the conventional single-axis cylindrical treadmill shown in Figure 1A. Although classified as complex locomotion, this paradigm generates only subtle differences in forelimb muscle activity/wrist movements and comparable changes in M1 activity when compared to simple locomotion on a linear treadmill (Beloozerova et al., 2010; Marlinski et al., 2012). Selectively blocking noradrenergic receptors in forelimb M1 significantly decreased the number of precise contralateral forepaw placements compared to sham controls (precise forepaw placements 60 min after: sham saline $86.8\% \pm 0.7\%$, noradrenergic receptor antagonists $70.5\% \pm 1.7\%$; n = 3 and 5, respectively; $p < 4.0 \times 10^{-4}$; Figure 6F) or ipsilateral forepaw placements

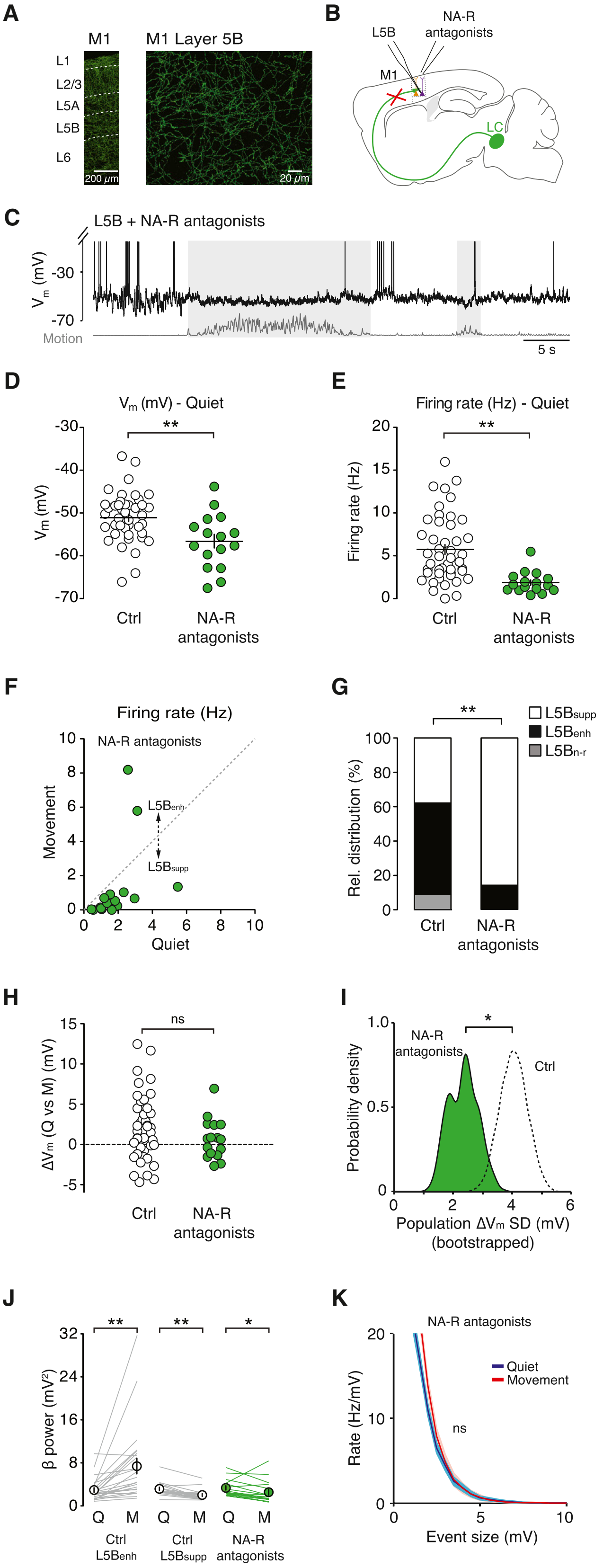

Figure 5. Blocking Input from the LC Reduces Behavioral State-Dependent Increase in Excitatory Drive in $L5B_{enh}$ Neurons

(A) Noradrenergic axons in the forelimb region of M1 were labeled using an anti-noradrenaline transporter antibody and secondary antibody conjugated to AlexaFluor 488.

(B) Schematic representation of an L5B pyramidal neuron recording after blocking noradrenergic input from the LC.

(C) Representative voltage trace from a L5B pyramidal neuron in the absence of noradrenergic input.

(D and E) Average V_m (D) and firing rate (E) of L5B pyramidal neurons during quiet wakefulness in the absence (open symbols, n = 45) and presence (green symbols, n = 16) of noradrenergic receptor (NA-R) antagonists. Filled circles represent data from individual neurons, black bars represent mean ± SEM. Control data (Ctrl) were taken from the dataset presented in Figure 1 for comparison. Mann-Whitney U test, $^*p < 0.017$, $^{**}p < 0.003$.

(F) Average firing rate of L5B pyramidal neurons in the presence of noradrenergic receptor antagonists (n = 16) during quiet wakefulness and movement. Filled circles represent data from individual neurons.

(G) Relative distributions of $L5B_{supp}$, $L5B_{enh}$, and $L5B_{n\text{-}r}$ neurons in the absence (Ctrl) and presence (NA-R antagonists, n = 16) of noradrenergic receptor antagonists. Control data (Ctrl) were taken from the dataset presented in Figure 1 for comparison. Chi-square test, $^{**}p < 0.01$.

(H) Change in average V_m (ΔV_m) during movement in the absence (Ctrl, n = 41) and presence of noradrenergic receptor antagonists (green symbols, n = 16). Control data (Ctrl) were taken from Figure 1 for comparison. Mann-Whitney U test; ns, non-significant.

(I) Probability density distributions of ΔV_m variability across the L5B pyramidal neuron population (Ctrl and NA-R antagonists), measured as the SD of the ΔV_m distributions shown in (H) (Population ΔV_m SD) using bootstrap analysis (10,000 bootstrap replicates). Black dashed line represents population ΔV_m variability distribution in control (Ctrl), and green shading represents population ΔV_m variability distribution following noradrenergic receptor blockade. Control data (Ctrl) were taken from Figure 1 for comparison. F test, $^*p < 0.025$.

(J) Average L5B pyramidal neuron V_m power in the β frequency band (12–30 Hz) during quiet wakefulness and movement in the presence (Ctrl: $L5B_{supp}$, n = 17 and $L5B_{enh}$, n = 24) and absence of noradrenergic input (NA-R antagonists, n = 16). Solid lines represent data from individual neurons, symbols represent mean ± SEM. Control data (Ctrl) were taken from Figure 3 for comparison. $^*p < 0.05$.

(K) Average rate density of compound synaptic events in L5B pyramidal neurons during quiet wakefulness (blue) and movement (red) in the absence of noradrenergic input (n = 16). Compare with Figures 3I and 3J.

See also Figures S5 and S6.

(data not shown). Together, our results demonstrate that noradrenergic input from the LC is necessary for controlling M1 output and motor coordination during self-paced voluntary movement.

DISCUSSION

In this paper we present three main findings. First, we show that behavioral state-dependent bidirectional modulation of M1 output is governed by two opposing subthreshold mechanisms (1) a global decrease in network-driven, slow, large-amplitude V_m fluctuations, which reduced V_m variability, spike probability, and firing rates in $L5B_{supp}$ neurons; and (2) a coincident increase in excitatory drive in a subpopulation of L5B neurons ($L5B_{enh}$), which increased spike probability and firing rates. Second, we demonstrate that the movement-related tonic depolarization in $L5B_{enh}$ neurons requires the interplay between ascending input from the motor thalamus, which maintained V_m near threshold, and noradrenergic input from the LC, which enhanced the SBR for movement-evoked responses. Finally, we show that selective

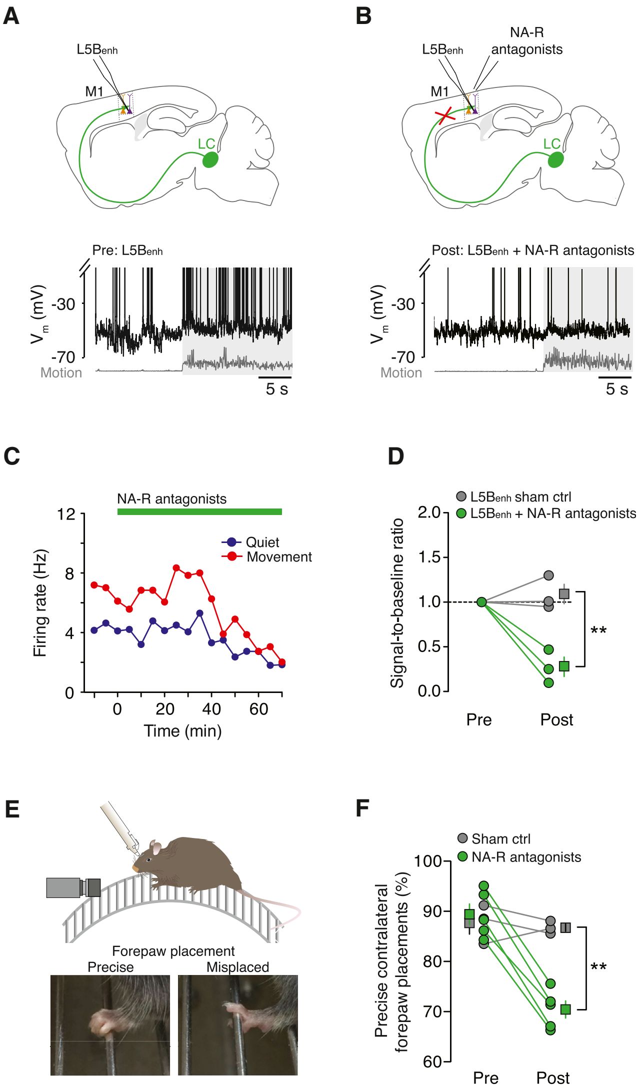

Figure 6. Blocking Noradrenergic Input Reduces the SBR in $L5B_{enh}$ Neurons and Impairs Contralateral Forepaw Motor Coordination

(A and B) Schematic representation of the experimental design and representative voltage traces from an $L5B_{enh}$ pyramidal neuron prior to (A) and after (B, >30 min) topical application of noradrenergic receptor antagonists. Gray shading depicts movement.

(C) Time course shows quiet (blue) and movement-related (red) firing rates in an $L5B_{enh}$ pyramidal neuron before and after noradrenergic receptor blockade (green bar).

(D) Movement-induced SBR in $L5B_{enh}$ pyramidal neurons before and >30 min after application of noradrenergic receptor antagonists (green circles, n = 3) or saline (gray circles, n = 3). Square symbols represent mean ± SEM. Unpaired t test, $^{**}p < 0.01$.

(E) Behavioral assessment of forepaw placement precision in head-fixed mice mounted on a single-axis, cylindrical runged treadmill. Video sequences were used to score contralateral and ipsilateral forepaw placements.

(F) Percentage of precise contralateral forepaw placements before and 60 min after application of noradrenergic receptor antagonists (green circles, n = 5) or saline (gray circles, n = 3). Square symbols represent mean ± SEM. Unpaired t test, $^{**}p < 0.01$.

See also Figure S6.

blockade of noradrenaline signaling in forelimb M1 reduces motor coordination in the contralateral forelimb, resulting in a significant decrease in precise forepaw placements. Together, our findings reveal the subthreshold and circuit mechanisms that regulate behavioral state-dependent bidirectional modulation of M1 output during self-paced, voluntary movement.

Behavioral State-Dependent Modulation of Input-Output Transformations in L5

Physiologically relevant changes in V_m variance or mean have been shown to profoundly influence neuronal input-output transformations (Chance et al., 2002; Hô and Destexhe, 2000). But, this has never been explored in L5 pyramidal neurons in the awake cortex. Our current injection experiments in vivo demonstrate that changes in V_m SD ($L5B_{supp}$) or V_m SD and mean ($L5B_{enh}$) have quantitatively similar—but functionally opposing—effects on spike probability when examined over a behaviorally relevant input amplitude range (1–10 mV). This similarity arises due to the non-linear relationship between V_m and firing probability, such that moderate depolarization can produce a non-linear additive increase in the sensitivity of a neuron to small-amplitude inputs, while decreased V_m SD produces a divisive reduction in input sensitivity (Brozović et al., 2008; Murphy and Miller, 2003). The behavioral state-dependent bidirectional modulation of neuronal responsiveness in L5B pyramidal neurons (i.e., increased or decreased spike probability) could facilitate the routing of sensorimotor information through specific M1 neuronal assemblies during movement.

Local and Long-Range Inputs to M1 during Self-Paced Voluntary Movement

M1 receives input from a variety of brain areas (e.g., ipsilateral primary and secondary somatosensory cortices, secondary motor cortex, and orbitofrontal cortex), with ascending input from motor thalamus and descending input from L2/3 providing strong feedforward excitation directly to L5B neurons (Castro-Alamancos and Connors, 1996; Hooks et al., 2013; Weiler et al., 2008). We found that movement did not affect firing rates in our sample of L2/3 pyramidal neurons, suggesting that descending excitation from L2/3 may not be the primary source of the tonic depolarization in $L5B_{enh}$ neurons during simple locomotion on a cylindrical treadmill. These findings are in direct contrast to a previous study by Dombeck and colleagues, where locomotion on a spherical treadmill resulted in large-scale, clustered activity of L2/3 neurons in mouse forelimb M1 (Dombeck et al., 2009). The reason for this discrepancy is unclear. One possibility is that our recordings undersampled L2/3 population activity; however, if locomotion induced dense activity similar to that observed in Dombeck et al., (2009), we would have expected to observe movement-related firing rate changes in a significant proportion of our intracellular recordings. Moreover, Dombeck and colleagues did not identify individual neuronal subtypes, so the large-scale activity could be due, in part, to elevated L2/3 interneuron activity. Alternatively, dense L2/3 activity could result from mice having to balance and oppose the inertial forces of a rotating air-cushioned ball when changing direction. In principle, this could generate a sensorimotor

OPEN
ACCESS
CellPress

mismatch between the rotational direction of the ball and the intended movement trajectory of the mouse, leading to continuous sensory feedback to M1. Thus, it will be important for future studies to investigate the extent to which descending L2/3 input contributes to L5B frequency modulation during simple versus complex motor behaviors.

Direct thalamic input to cortical pyramidal neurons can drive output by reducing slow V_m fluctuations, depolarizing mean V_m, and reducing the distance to threshold (Castro-Alamancos and Connors, 1996; Constantinople and Bruno, 2013; Hirata and Castro-Alamancos, 2010; Poulet et al., 2012). Consistent with previous findings in sensory cortex, we found that inactivation of thalamus (VA/VL region) increased slow, large-amplitude V_m fluctuations, but did not abolish the activated state during behavior (Hirata and Castro-Alamancos, 2010; Poulet et al., 2012), suggesting thalamic input to M1 is sufficient but not necessary for generating the activated cortical state. However, ascending motor thalamic input—direct or indirect—appears to be necessary for maintaining the average V_m relatively close to threshold, providing a mechanism whereby subtle changes in input structure can generate positive or negative changes in M1 output during movement.

Noradrenergic Neuromodulation

We have shown that noradrenaline release during different behavioral states (i.e., quiet wakefulness versus movement) has profound effects on M1 cortical dynamics. Similar to thalamic inactivation, blocking noradrenergic input from the LC reduced basal firing rates by hyperpolarizing mean V_m and increasing distance to threshold, suggesting tonic input from both the LC and motor thalamus are necessary to generate moderate firing rates in L5B pyramidal neurons during quiet wakefulness. Our finding that noradrenaline generated a tonic depolarization in a selected subpopulation of L5B pyramidal neurons differs from results obtained in superficial layers of sensory cortex (Polack et al., 2013), highlighting the importance of understanding the sublayer-specific effects of noradrenaline in the awake cortex. In primary visual cortex (V1), locomotion-dependent noradrenaline release generates a global depolarization of L2/3 pyramidal neurons, which may enhance visual attention by increasing the gain and signal-to-noise ratio of visually evoked responses (Bennett et al., 2013; Polack et al., 2013).

The fact that we also observed a movement-related tonic depolarization in the majority of M1 L2/3 pyramidal neurons, which was abolished by noradrenergic receptor blockade (Figure S6), suggests that noradrenaline may differentially affect cortical processing in superficial versus deep-layer pyramidal neurons during active behavior. Topical application of high concentrations of noradrenergic receptor antagonists could potentially produce off-target effects. However, given that low doses of antagonists affect L2/3 V_m dynamics in the same way as high concentrations, albeit smaller in magnitude, suggests relatively selective antagonist effects (Polack et al., 2013). Although noradrenaline appears to underpin the majority of the locomotion-dependent V_m depolarization in V1, cholinergic disinhibition of somatostatin-containing interneurons is likely to further enhance behavioral state-dependent gain modulation (Fu et al., 2014). We did not directly test the role of acetylcholine in our study, but given its importance in regulating V_m dynamics in other cortical areas (Constantinople and Bruno, 2011; Eggermann et al., 2014; Favero et al., 2012; Fu et al., 2014; Polack et al., 2013), it will be important for future studies to investigate its role in M1 during motor behavior.

How does noradrenaline generate the tonic depolarization in $L5B_{enh}$ neurons during movement? Previous studies have shown that noradrenaline modulates voltage-dependent and voltage-independent potassium conductances and hyperpolarization-activated cyclic nucleotide-gated (HCN) channels, thus generating a tonic depolarization by reducing the spike after-hyperpolarization and prolonging the depolarizing effect of excitatory synaptic inputs (Favero et al., 2012; Sheets et al., 2011; Wang et al., 2007; Wang and McCormick, 1993). This combined with modulation of basal firing rates is thought to alter the signal-to-noise ratio of neuronal responses to synaptic input (Berridge and Waterhouse, 2003). Alternatively, we cannot rule out the possibility that noradrenaline selectively reduces the activity of local GABAergic interneurons, thus releasing $L5B_{enh}$ neurons from inhibition and generating a depolarization in V_m. Therefore, identifying the specific expression patterns and subcellular localization of α and β adrenergic receptors in excitatory and inhibitory neurons in M1 will be an important next step in understanding how noradrenaline exerts its sublayer- and cell-type specific effects.

Functional Implications

What function does behavioral state-dependent bidirectional modulation of L5 output serve? The flexible modulation of L5B output channels (PT type and IT type) provides an important control mechanism to modulate and update activity patterns in downstream cortical and subcortical areas during changes in behavioral state. PT output provides online information about the state of cortical activation to downstream areas involved in motor control. This continuously updating flow of information generates a basic pattern of input to brainstem and spinal cord circuits in order to generate appropriate behavioral responses in accordance with changes in behavioral state. We demonstrate that blocking noradrenergic receptors in forelimb M1 selectively disrupts motor coordination in the contralateral forepaw, thus confirming the importance of noradrenergic neuromodulation and descending M1 output for motor control. Given that output from sensory and non-sensory cortices have overlapping downstream targets (Hattox and Nelson, 2007; Kita and Kita, 2012), we speculate that our findings might generalize to other cortical output layers and that noradrenergic neuromodulation and network-driven input changes are common mechanisms to bidirectionally modulate cortical output during active behavior.

EXPERIMENTAL PROCEDURES

Animals and Surgery

All experiments and procedures involving animals were approved by the University of Edinburgh local ethical review committee and performed under license from the UK Home Office in accordance with the Animal (Scientific Procedures) Act 1986. Male C57BL/6 mice (5–12 weeks old, 20–25 g, two to six animals per cage, maintained on a reversed 12:12-hr light:dark cycle with ad libitum access to food and water) were implanted with a small lightweight headplate (0.75 g) using cyano-acrylate glue and dental acrylic. All surgical

procedures were performed under 1.5% isoflurane anesthesia. After 24-to 48-hr recovery, a craniotomy (300 × 300 μm) was performed and the dura removed above the right forelimb region of M1. Using intracortical microstimulation (see Supplemental Experimental Procedures), the center of $M1_{FL}$ was located 0.7 mm rostral and 1.5 mm lateral to bregma. The craniotomy was sealed with (1.5%) agar and Kwik-Cast sealant (WPI) and mice recovered for 2 hr before recording commenced.

Motion Index and Motor Pattern Discrimination

An optical encoder was used to capture movement of the treadmill and locomotion was defined as periods with speed > 0.01 m/s for more than 2 s. Changes in behavioral state (quiet wakefulness to movement [grooming or locomotion]) were captured using an elevated, front-mounted, moderate-speed (60 frames/s) digital video camera synchronized with each electrophysiological recording.

In Vivo Electrophysiology and Pharmacology

Mice were habituated to the head restraint and experimental setup for 45–60 min before each recording session. Whole-cell patch-clamp recordings were obtained from awake head-restrained mice at a depth of 180–420 μm (layer 2/3) or 620–880 μm (layer 5B) from the pial surface, using a Multiclamp 700B amplifier (Molecular Devices). The signal was filtered at 10 kHz and acquired at 20 kHz using PClamp 10 software in conjunction with a DigiData 1440 DAC interface (Molecular Devices). No bias current was injected during recordings and the membrane potential was not corrected for junction potential. Resting membrane potentials were recorded immediately after attaining the whole-cell configuration (break-in). Series resistances (R_s) ranged from 15 to 40 MΩ and experiments were terminated if R_s exceeded 60 MΩ. Current injection was performed only if R_s < 35 MΩ. Patch pipettes (5–7 MΩ) were filled with internal solution (285–295 mOsm) containing: 135 mM K-gluconate, 4 mM KCl, 10 mM HEPES, 10 mM sodium phosphocreatine, 2 mM MgATP, 2 mM Na_2ATP, 0.5 mM Na_2GTP, and 2 mg/ml biocytin (pH adjusted to 7.2 with KOH). External solution contained: 150 mM NaCl, 2.5 mM KCl, 10 mM HEPES, 1 mM $CaCl_2$, and 1 mM $MgCl_2$ (adjusted to pH 7.3 with NaOH).

For inactivation of the motor thalamus, the $GABA_A$ receptor agonist muscimol (1 mM muscimol hydrobromide, Sigma-Aldrich) was dissolved in external solution, and 100 nl was stereotaxically injected into the right VA/VL complex (−1 mm caudal, 1.1 mm lateral to bregma, and 3.2 mm below the pial surface). Whole-cell patch-clamp recordings of L5B pyramidal neurons were carried out approximately 2 hr after muscimol injection.

To block noradrenergic receptors, a mixture of α1, α2, and β noradrenergic receptor antagonists (1 mM prazosin, yohimbine, and propranolol; Sigma-Aldrich) in external solution (adjusted to pH 7.3) was applied topically to the craniotomy and recordings were performed >40 min after antagonist application.

Functional Classification of Recorded Neurons

For each L5B cell, we (1) divided quiet periods into 1-s epochs; (2) randomly assigned each epoch into two groups, quiet 1 (q1) and quiet 2 (q2); and (3) calculated the firing rate difference between q1 and q2. We repeated steps (1) to (3) 10,000 times for each cell to obtain the distribution probability of the difference of firing rate in q1 and q2 (see Figures 1C–1E). If during movement the firing rate change was higher than the 99th percentile or lower than the 1st percentile, we classified the neuron as enhanced or suppressed, respectively. If the firing rate change fell within the first and 99th percentiles, the cell was classified as non-responding.

Statistical Analyses

Summary data are expressed as mean ± SEM unless otherwise stated. Statistical significance was determined using Wilcoxon signed-rank tests (paired data) and rank-sum tests (unpaired data) unless otherwise stated. Wilcoxon signed-rank tests on the areas underlying the rate-density curves were used in Figures 3I and 3J. The relative distribution of functional phenotypes ($L5B_{supp}$, $L5B_{enh}$, and $L5B_{n\text{-}r}$) was analyzed using Pearson chi-square test statistics (based on 10^6 permutations). Statistical significance in population ΔV_m variability (Figures 4J and 5I) was determined using two-sample F tests. To depict the variance of the underlying populations, 10,000 bootstrap samples (random samples with replacement) of each population were taken, and a probability density function of the variances of the bootstrap samples was plotted. For statistical tests, $p < 0.05$ was considered significant (*$p < 0.05$ and **$p < 0.01$). For repeated statistical comparisons with the control dataset, resulting p values were compared to Bonferroni-corrected alpha levels and stated accordingly.

SUPPLEMENTAL INFORMATION

Supplemental Information includes Supplemental Experimental Procedures, six figures, and two tables and can be found with this article online at http://dx.doi.org/10.1016/j.celrep.2015.04.042.

AUTHOR CONTRIBUTIONS

J.S., P.P., M.C.W.v.R., and I.D. designed the experiments. All experiments were carried out by J.S., P.P., and J.D. with help from I.D. Analysis was performed by P.P., J.S., M.P., and J.D. A.D. wrote MATLAB scripts to extract forepaw motion statistics. Neuronal reconstructions were carried out by J.S., P.P., J.D., and I.D. I.D., P.P., and J.S. wrote the manuscript, and all authors contributed to discussion and interpretation of the results.

ACKNOWLEDGMENTS

We are grateful to M. Nolan, M. London, P. Magill, K. Harris, T. Branco, L. Acerbi, M. Ludwig, N. Rochefort, and members of the I.D. lab for helpful discussions and for comments on the manuscript; M. Cowper-Coles for neuronal reconstructions; and T. Theil for advice on CTIP2/SATB2 immunohistochemistry. Confocal microscopy was performed in the IMPACT Imaging Facility at the University of Edinburgh. This work was supported by grants from the Engineering and Physical Sciences Research Council, Eurospin Erasmus Mundus Program, Leopoldina fellowship program (LPDS 2012-11 to J.S.), the Shirley Foundation, Medical Research Council (MR/K014137/1, awarded to P. Kind), and a Wellcome Trust Career Development fellowship (WT086602MF) to I.D.

Received: June 11, 2014
Revised: March 16, 2015
Accepted: April 20, 2015
Published: May 14, 2015

REFERENCES

Armstrong, D.M., and Drew, T. (1984a). Discharges of pyramidal tract and other motor cortical neurones during locomotion in the cat. J. Physiol. *346*, 471–495.

Armstrong, D.M., and Drew, T. (1984b). Locomotor-related neuronal discharges in cat motor cortex compared with peripheral receptive fields and evoked movements. J. Physiol. *346*, 497–517.

Beloozerova, I.N., Sirota, M.G., and Swadlow, H.A. (2003). Activity of different classes of neurons of the motor cortex during locomotion. J. Neurosci. *23*, 1087–1097.

Beloozerova, I.N., Farrell, B.J., Sirota, M.G., and Prilutsky, B.I. (2010). Differences in movement mechanics, electromyographic, and motor cortex activity between accurate and nonaccurate stepping. J. Neurophysiol. *103*, 2285–2300.

Bennett, C., Arroyo, S., and Hestrin, S. (2013). Subthreshold mechanisms underlying state-dependent modulation of visual responses. Neuron *80*, 350–357.

Berridge, C.W., and Waterhouse, B.D. (2003). The locus coeruleus-noradrenergic system: modulation of behavioral state and state-dependent cognitive processes. Brain Res. Brain Res. Rev. *42*, 33–84.

Brozović, M., Abbott, L.F., and Andersen, R.A. (2008). Mechanism of gain modulation at single neuron and network levels. J. Comput. Neurosci. *25*, 158–168.

Carter, M.E., Yizhar, O., Chikahisa, S., Nguyen, H., Adamantidis, A., Nishino, S., Deisseroth, K., and de Lecea, L. (2010). Tuning arousal with optogenetic modulation of locus coeruleus neurons. Nat. Neurosci. *13*, 1526–1533.

Castro-Alamancos, M.A. (2004). Absence of rapid sensory adaptation in neocortex during information processing states. Neuron *41*, 455–464.

Castro-Alamancos, M.A., and Connors, B.W. (1996). Short-term plasticity of a thalamocortical pathway dynamically modulated by behavioral state. Science *272*, 274–277.

Castro-Alamancos, M.A., and Gulati, T. (2014). Neuromodulators produce distinct activated states in neocortex. J. Neurosci. *34*, 12353–12367.

Castro-Alamancos, M.A., and Oldford, E. (2002). Cortical sensory suppression during arousal is due to the activity-dependent depression of thalamocortical synapses. J. Physiol. *541*, 319–331.

Chance, F.S., Abbott, L.F., and Reyes, A.D. (2002). Gain modulation from background synaptic input. Neuron *35*, 773–782.

Constantinople, C.M., and Bruno, R.M. (2011). Effects and mechanisms of wakefulness on local cortical networks. Neuron *69*, 1061–1068.

Constantinople, C.M., and Bruno, R.M. (2013). Deep cortical layers are activated directly by thalamus. Science *340*, 1591–1594.

Costa, R.M., Cohen, D., and Nicolelis, M.A. (2004). Differential corticostriatal plasticity during fast and slow motor skill learning in mice. Curr. Biol. *14*, 1124–1134.

Cowan, R.L., and Wilson, C.J. (1994). Spontaneous firing patterns and axonal projections of single corticostriatal neurons in the rat medial agranular cortex. J. Neurophysiol. *71*, 17–32.

Crochet, S., and Petersen, C.C. (2006). Correlating whisker behavior with membrane potential in barrel cortex of awake mice. Nat. Neurosci. *9*, 608–610.

Deuchars, J., West, D.C., and Thomson, A.M. (1994). Relationships between morphology and physiology of pyramid-pyramid single axon connections in rat neocortex *in vitro*. J. Physiol. *478*, 423–435.

Dombeck, D.A., Graziano, M.S., and Tank, D.W. (2009). Functional clustering of neurons in motor cortex determined by cellular resolution imaging in awake behaving mice. J. Neurosci. *29*, 13751–13760.

Eggermann, E., Kremer, Y., Crochet, S., and Petersen, C.C. (2014). Cholinergic signals in mouse barrel cortex during active whisker sensing. Cell Rep. *9*, 1654–1660.

Favero, M., Varghese, G., and Castro-Alamancos, M.A. (2012). The state of somatosensory cortex during neuromodulation. J. Neurophysiol. *108*, 1010–1024.

Forssberg, H., Grillner, S., Halbertsma, J., and Rossignol, S. (1980). The locomotion of the low spinal cat. II. Interlimb coordination. Acta Physiol. Scand. *108*, 283–295.

Fu, Y., Tucciarone, J.M., Espinosa, J.S., Sheng, N., Darcy, D.P., Nicoll, R.A., Huang, Z.J., and Stryker, M.P. (2014). A cortical circuit for gain control by behavioral state. Cell *156*, 1139–1152.

Grillner, S. (1981). Control of locomotion in bipeds, tetrapods and fish. In Handbook of Physiology (Bethesda: American Physiology Society), pp. 1179–1236.

Grillner, S., and Zangger, P. (1979). On the central generation of locomotion in the low spinal cat. Exp. Brain Res. *34*, 241–261.

Hattox, A.M., and Nelson, S.B. (2007). Layer V neurons in mouse cortex projecting to different targets have distinct physiological properties. J. Neurophysiol. *98*, 3330–3340.

Hirata, A., and Castro-Alamancos, M.A. (2010). Neocortex network activation and deactivation states controlled by the thalamus. J. Neurophysiol. *103*, 1147–1157.

Hô, N., and Destexhe, A. (2000). Synaptic background activity enhances the responsiveness of neocortical pyramidal neurons. J. Neurophysiol. *84*, 1488–1496.

Hooks, B.M., Mao, T., Gutnisky, D.A., Yamawaki, N., Svoboda, K., and Shepherd, G.M. (2013). Organization of cortical and thalamic input to pyramidal neurons in mouse motor cortex. J. Neurosci. *33*, 748–760.

Kaneko, T., Caria, M.A., and Asanuma, H. (1994). Information processing within the motor cortex. II. Intracortical connections between neurons receiving somatosensory cortical input and motor output neurons of the cortex. J. Comp. Neurol. *345*, 172–184.

Kita, T., and Kita, H. (2012). The subthalamic nucleus is one of multiple innervation sites for long-range corticofugal axons: a single-axon tracing study in the rat. J. Neurosci. *32*, 5990–5999.

Leone, D.P., Srinivasan, K., Chen, B., Alcamo, E., and McConnell, S.K. (2008). The determination of projection neuron identity in the developing cerebral cortex. Curr. Opin. Neurobiol. *18*, 28–35.

Lorang, D., Amara, S.G., and Simerly, R.B. (1994). Cell-type-specific expression of catecholamine transporters in the rat brain. J. Neurosci. *14*, 4903–4914.

Marlinski, V., Nilaweera, W.U., Zelenin, P.V., Sirota, M.G., and Beloozerova, I.N. (2012). Signals from the ventrolateral thalamus to the motor cortex during locomotion. J. Neurophysiol. *107*, 455–472.

Murphy, B.K., and Miller, K.D. (2003). Multiplicative gain changes are induced by excitation or inhibition alone. J. Neurosci. *23*, 10040–10051.

Orlovsky, G.N. (1972). The effect of different descending systems on flexor and extensor activity during locomotion. Brain Res. *40*, 359–371.

Polack, P.O., Friedman, J., and Golshani, P. (2013). Cellular mechanisms of brain state-dependent gain modulation in visual cortex. Nat. Neurosci. *16*, 1331–1339.

Poulet, J.F., Fernandez, L.M., Crochet, S., and Petersen, C.C. (2012). Thalamic control of cortical states. Nat. Neurosci. *15*, 370–372.

Reyes, A., and Sakmann, B. (1999). Developmental switch in the short-term modification of unitary EPSPs evoked in layer 2/3 and layer 5 pyramidal neurons of rat neocortex. J. Neurosci. *19*, 3827–3835.

Sheets, P.L., Suter, B.A., Kiritani, T., Chan, C.S., Surmeier, D.J., and Shepherd, G.M.G. (2011). Corticospinal-specific HCN expression in mouse motor cortex: I(h)-dependent synaptic integration as a candidate microcircuit mechanism involved in motor control. J. Neurophysiol. *106*, 2216–2231.

Steriade, M., Amzica, F., and Nuñez, A. (1993a). Cholinergic and noradrenergic modulation of the slow (approximately 0.3 Hz) oscillation in neocortical cells. J. Neurophysiol. *70*, 1385–1400.

Steriade, M., McCormick, D.A., and Sejnowski, T.J. (1993b). Thalamocortical oscillations in the sleeping and aroused brain. Science *262*, 679–685.

Steriade, M., Nuñez, A., and Amzica, F. (1993c). A novel slow (< 1 Hz) oscillation of neocortical neurons in vivo: depolarizing and hyperpolarizing components. J. Neurosci. *13*, 3252–3265.

Timofeev, I., Grenier, F., and Steriade, M. (2001). Disfacilitation and active inhibition in the neocortex during the natural sleep-wake cycle: an intracellular study. Proc. Natl. Acad. Sci. USA *98*, 1924–1929.

Ueno, M., and Yamashita, T. (2011). Kinematic analyses reveal impaired locomotion following injury of the motor cortex in mice. Exp. Neurol. *230*, 280–290.

Wang, Z., and McCormick, D.A. (1993). Control of firing mode of corticotectal and corticopontine layer V burst-generating neurons by norepinephrine, acetylcholine, and 1S,3R-ACPD. J. Neurosci. *13*, 2199–2216.

Wang, M., Ramos, B.P., Paspalas, C.D., Shu, Y., Simen, A., Duque, A., Vijayraghavan, S., Brennan, A., Dudley, A., Nou, E., et al. (2007). Alpha2A-adrenoceptors strengthen working memory networks by inhibiting cAMP-HCN channel signaling in prefrontal cortex. Cell *129*, 397–410.

Weiler, N., Wood, L., Yu, J., Solla, S.A., and Shepherd, G.M. (2008). Top-down laminar organization of the excitatory network in motor cortex. Nat. Neurosci. *11*, 360–366.

Cell Reports

Supplemental Information

Cellular Mechanisms Underlying Behavioral State-Dependent Bidirectional Modulation of Motor Cortex Output

Julia Schiemann, Paolo Puggioni, Joshua Dacre, Miha Pelko, Aleksander Domanski, Mark C.W. van Rossum, and Ian Duguid

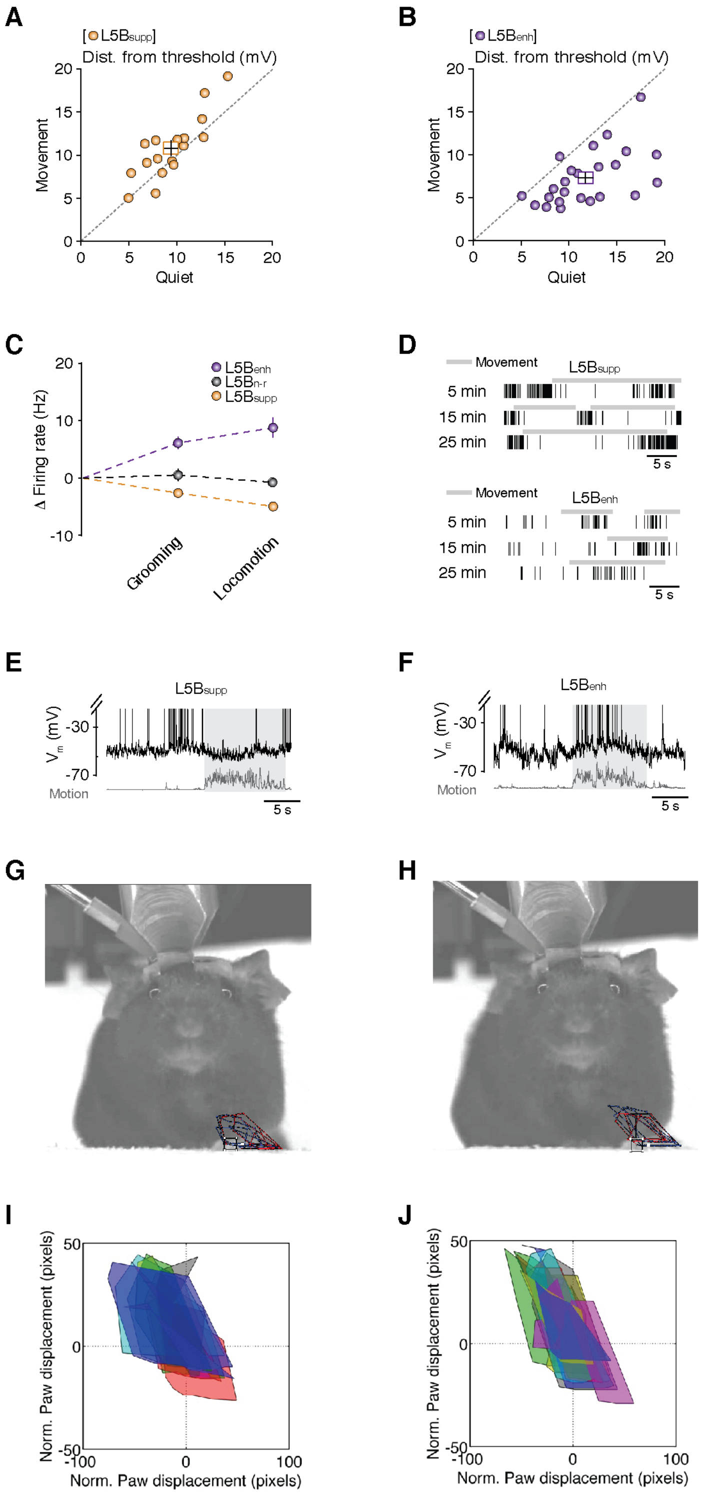

Figure S1. Membrane potential dynamics of $L5B_{supp}$ and $L5B_{enh}$ neurons during quiet wakefulness and self-paced voluntary movement. Related to Figure 1.

(**A-B**) Average distance from threshold in $L5B_{supp}$ (A, n = 17) and $L5B_{enh}$ (B, n = 24) pyramidal neurons during quiet wakefulness and movement. Filled circles represent data from individual neurons while square symbols represent mean ± s.e.m..

(**C**) Average firing rate changes in $L5B_{supp}$ (yellow, n = 17), $L5B_{non\text{-}responding}$ (gray, n = 4) and $L5B_{enh}$ (purple, n = 24) neurons during grooming or locomotion.

(**D**) Representative spike raster plots from a $L5B_{supp}$ (upper panel) and a $L5B_{enh}$ (lower panel) neuron during movement phases occurring 5, 15 and 25 minutes after 'break-through'. Note functional classification of neurons is unchanged over time.

(**E-F**) Representative voltage traces from a $L5B_{supp}$ (E) and $L5B_{enh}$ (F) pyramidal neuron recorded in the same mouse during similar forelimb movements (gray shading) as characterized in G-J.

(**G-H**) Video tracking of contralateral forelimb movements – repeated swing/stance cycles – associated with activity in a $L5B_{supp}$ (G) and $L5B_{enh}$ (H) neuron. Tracking lines represent similar forelimb movements during the 25 frames preceding (blue) and 25 frames after (red) the present video frame.

(**I-J**) Extracted vertical and horizontal paw displacements of individual contralateral forelimb step cycles associated with activity in a $L5B_{supp}$ (I) and $L5B_{enh}$ (J) neuron. Individual steps are represented by different colors. Quantification of the similarity of forelimb movements identified no significant difference. Step path circumference: $L5B_{supp}$ neuron 211 ± 15 pixels, $L5B_{enh}$ 238 ± 16 pixels, p = 0.23. Step path area: $L5B_{supp}$ neuron 2018 ± 313 pixels, $L5B_{enh}$ 2197 ± 243 pixels, p = 0.65.

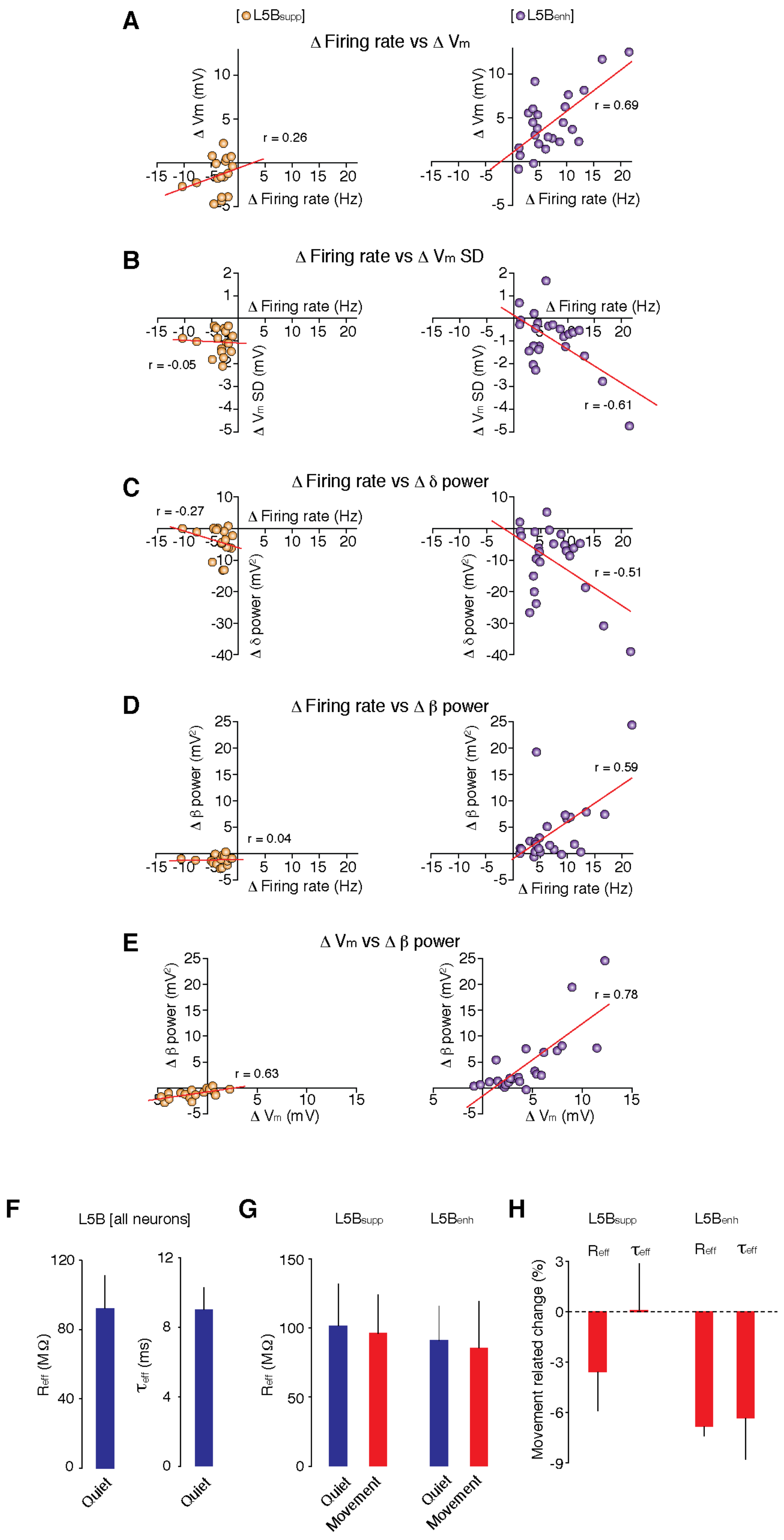

Figure S2. Movement-related changes in subthreshold V_m dynamics, firing rates, effective input resistance and membrane time constant in L5B pyramidal neurons. Related to Figures 1, 2 and 3.

(**A-D**) Relationship between Δ firing rate and Δ V_m (A), Δ V_m SD (B), Δ V_m power in δ (1.5 – 4 Hz, C) and β (12 – 30 Hz, D) frequency bands in $L5B_{supp}$ (yellow, *n* = 17) and $L5B_{enh}$ (purple, *n* = 24) neurons during movement.

(**E**) Relationship between Δ V_m and Δ V_m power in β (12 – 30 Hz) frequency band in $L5B_{supp}$ (yellow, *n* = 17) and $L5B_{enh}$ (purple, *n* = 24) neurons during self-paced voluntary movement.

(**F**) Input resistance (R_{eff}, left) and membrane time constant (τ_{eff}, right) of L5B neurons (*n* = 10) during quiet wakefulness.

(**G**) Input resistance of $L5B_{supp}$ (*n* = 5, left) and $L5B_{enh}$ (*n* = 5, right) pyramidal neurons during quiet wakefulness (blue) and movement (red).

(**H**) Movement-related changes in effective input resistance (R_{eff}) and membrane time constant (τ_{eff}) for $L5B_{supp}$ (*n* = 5) and $L5B_{enh}$ (*n* = 5) pyramidal neurons. Bars represent median values ± median absolute deviation.

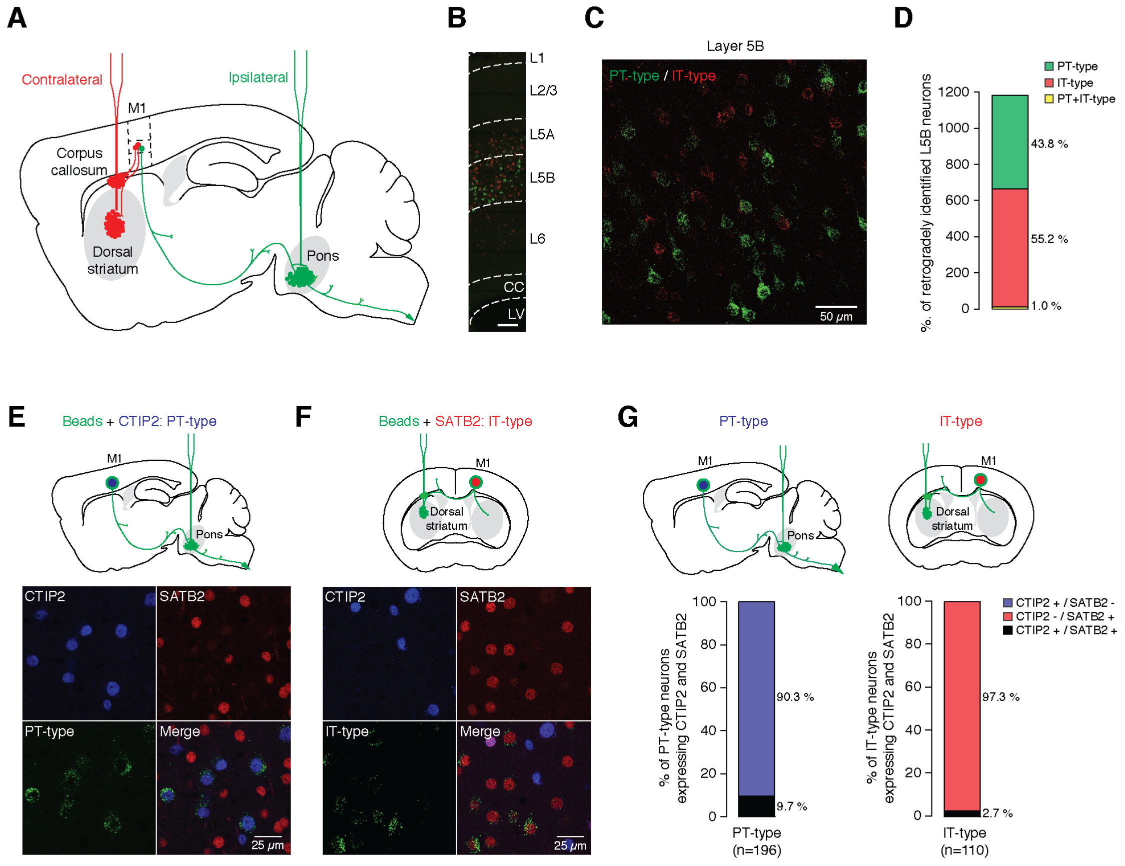

Figure S3. Classification of L5B projection neurons based on retrograde tracing and molecular marker expression. Related to Figure 1.

(**A**) Schematic parasagittal brain section illustrating retrograde fluorescent bead injection sites. Red fluorescent beads were injected into the contralateral dorsal striatum and corpus callosum to identify intratelencephalic (IT-type) projection neurons. Green fluorescent beads were injected into the ipsilateral pons to identify pyramidal tract (PT-type) projection neurons.

(**B-C**) Distribution of PT-type (green) and IT-type (red) projection neurons across different cortical layers (B) and within L5B (C) of the forelimb region of M1. LV: lateral ventricle; CC: corpus callosum; scale bar 100 μm.

(**D**) Relative distributions of PT-type (green) and IT-type (red) projection neurons in L5B of forelimb motor cortex (n = 1183 neurons, 50 slices, 6 mice).

(**E-F**) Single retrograde tracing and immunohistochemical staining confirming transcription factors CTIP2 and SATB2 are molecular markers for retrogradely identified PT-type (ipsilateral pons injection, E) and IT-type (contralateral dorsal striatum injection, F) neurons, respectively.

(**G**) Relative distribution of retrogradely identified L5B neurons expressing CTIP2 or SATB2 (n = 306 neurons, 24 slices, 4 mice).

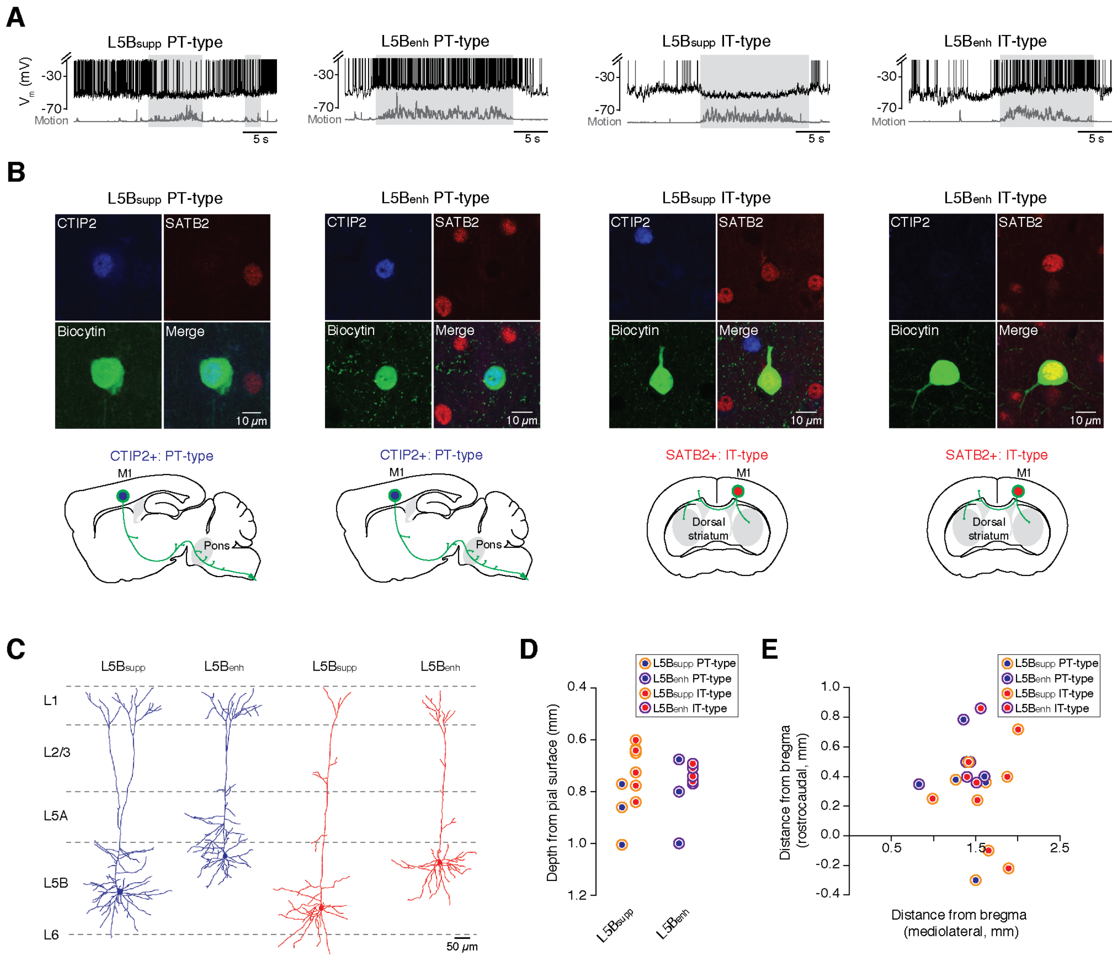

Figure S4. Projection class identity of individually recorded $L5B_{supp}$ and $L5B_{enh}$ neurons. Related to Figure 1.

(**A**) Representative voltage traces from identified: $L5B_{supp}$ PT-type; $L5B_{enh}$ PT-type; $L5B_{supp}$ IT-type; and $L5B_{enh}$ IT-type during quiet wakefulness and movement (light gray shading and dark gray motion index).

(**B**) Upper panels: single cell biocytin labeling (green) and post-hoc immunohistochemical staining for CTIP2 (blue) and SATB2 (red) confirmed the projection class identity of the individually recorded L5B pyramidal neurons shown in panel A. Lower panels: schematic brain sections showing $L5B_{supp}$ and $L5B_{enh}$ neurons contain a mixture of PT-type and IT-type projection neurons.

(**C**) Representative morphological reconstructions of $L5B_{supp}$ and $L5B_{enh}$ neurons color-coded by projection class identity (blue = PT-type, red = IT-type).

(**D**) Average depth of individually recorded $L5B_{supp}$ (n = 10) and $L5B_{enh}$ (n = 8) neurons color-coded by projection class identity.

(**E**) Neuroanatomical coordinates of individually recorded L5B pyramidal neurons plotted according to their position relative to bregma (n = 10 $L5B_{supp}$ neurons; n = 8 $L5B_{enh}$ neurons).

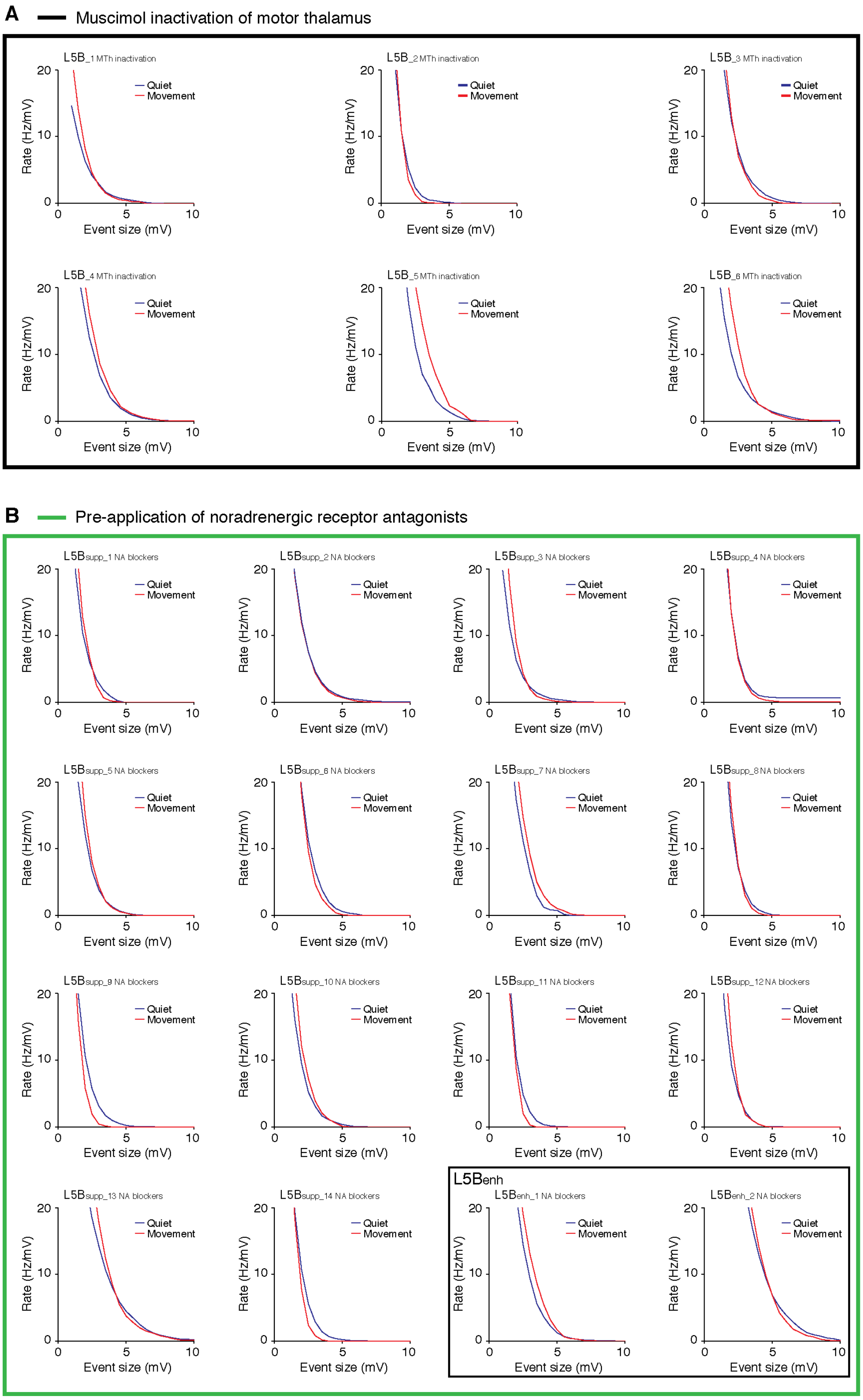

Figure S5. Compound event detection in L5B pyramidal neurons in the absence of motor thalamic input or noradrenergic input. Related to Figures 4 and 5.

(**A**) Average rate density of compound synaptic events in individual L5B pyramidal neurons (*n* = 6) during quiet wakefulness (blue) and movement (red). Note upper three panels depict neurons in which there was no change in rate density during movement, while bottom three panels depict cells that show an increased frequency of events.

(**B**) Average rate density of compound synaptic events in individual $L5B_{supp}$ (*n* = 14) and $L5B_{enh}$ (black box, *n* = 2) pyramidal neurons during quiet wakefulness (blue) and movement (red) in the absence of noradrenergic input. Note that the majority of L5B neurons do not show a significant increase in rate density during movement.

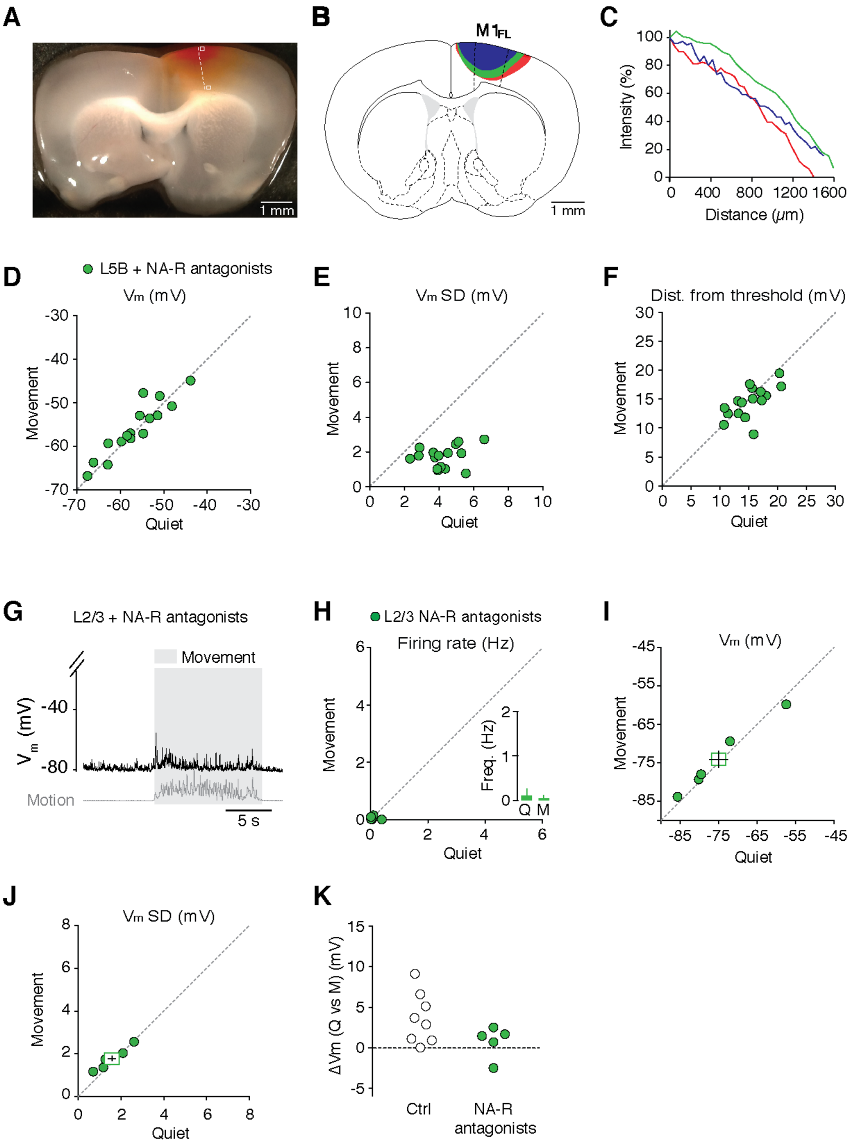

Figure S6. Drug diffusion through cortical layers and effects of noradrenergic receptor blockade on V_m dynamics of L5B and L2/3 pyramidal neurons during quiet wakefulness and movement. Related to Figures 5 and 6.

(**A**) Brightfield image showing dye diffusion through cortical layers after 60-minute application of phenol red (10-50 mM) to the surface of $M1_{FL}$.

(**B**) Schematic coronal brain section depicting dye diffusion through cortical layers after 60-minute application of phenol red (10-50 mM) to the surface in N = 3 mice. Note dye diffusion is centered around $M1_{FL}$ and does not penetrate subcortical areas.

(**C**) Dye intensity plotted as function of distance from the surface of the cortex (see dashed line in panel A).

(**D-F**) Average V_m (D), V_m SD (E) and distance from threshold (F) in L5B pyramidal neurons (green symbols, *n* = 16) during quiet wakefulness and movement in the absence of noradrenergic input. Filled circles represent data from individual neurons.

(**G**) Representative voltage trace from a L2/3 pyramidal neuron in the absence of noradrenergic input. Gray shading denotes movement.

(**H-J**) Average firing rate (H), V_m (I) and V_m SD (J) in L2/3 pyramidal neurons during quiet wakefulness and movement in the absence of noradrenergic input (*n* = 5). Filled circles represent data from individual neurons while square symbols represent mean ± s.e.m.. Inset in (H) depicts the average L2/3 neuron firing rate during quiet wakefulness (Q) and movement (M).

(**K**) Change in average V_m (Δ V_m) during movement in the absence (Ctrl, *n* = 8) and presence of noradrenergic receptor antagonists (green symbols, *n* = 5).

Table S1. Intrinsic properties of $M1_{FL}$ $L5B_{supp}$ and $L5B_{enh}$ pyramidal neurons recorded during quiet wakefulness. Related to Figure 1.

	$L5B_{supp}$			n	$L5B_{enh}$			n	p-value
**Firing rate (Hz)**	6.3	±	3.9	17	5.7	±	3.8	24	0.587
**Mean Vm (mV)**	-49.0	±	4.7	17	-52.0	±	5.3	24	0.061
**Std. Vm (mV)**	3.5	±	0.8	17	4.1	±	1.1	24	0.375
**C (pF)**	85	±	17	5	76	±	16	5	0.217
**Input Resistance (MΩ)**	85.3	±	23.7	5	96.4	±	17.5	5	0.487
**Membrane time constant (ms)**	8.6	±	2.0	5	7.8	±	1.1	5	0.625
**Spike threshold (mV)**	-39.1	±	5.2	17	-40.6	±	4.0	24	0.390
**Spike FWHM (ms)**	1.1	±	0.4	17	1.0	±	0.4	24	0.420
**Spike dV/dt peak:trough**	3.0	±	0.8	17	3.0	±	0.6	24	0.989
**Accommodation**	0.6	±	0.3	4	0.8	±	0.4	7	0.297
**Sag (%)**	3.8	±	2.4	6	4.3	±	4.4	15	0.974

Table S2. Intrinsic properties of identified $M1_{FL}$ PT-type and IT-type projection neurons recorded during quiet wakefulness. Related to Figure 1.

	IT-type			n	PT-type			n	p-value
**Firing rate (Hz)**	4.0	±	2.9	12	6.7	±	4.7	6	0.125
**Mean Vm (mV)**	-48.6	±	2.6	12	-47.6	±	7.1	6	0.553
**Std. Vm (mV)**	4.2	±	1.1	12	3.3	±	0.8	6	0.151
**Spike threshold (mV)**	-37.1	±	4.1	12	-39.7	±	7.1	6	0.094
**Spike FWHM (ms)**	1.2	±	0.3	12	1.1	±	0.4	6	0.250
**Spike dV/dt peak:trough**	3.3	±	0.6	12	2.8	±	0.5	6	0.068
**Accommodation**	0.6	±	0.3	5	0.9	±	0.6	3	0.393
**Sag (%)**	3.5	±	2.4	6	7.2	±	6.0	5	0.247

Supplemental Experimental Procedures

Mapping M1$_{FL}$ using intracortical microstimulation

The forelimb region of the primary motor cortex (M1$_{FL}$) was mapped using intracortical microstimulation (ICMS) in 7 male mice under 0.5 % isoflurane anesthesia. Glass micropipettes (1-2 MΩ) filled with extracellular solution were targeted to layer 5 (620 – 800 μm) and cathodal pulse trains (50 ms train duration, 30-350 μA, 300 μs pulse duration, 333 Hz) (A.M.P.I. Iso-flex) were applied through the stimulating electrode and slowly increased until contralateral forelimb muscle twitches could be observed. Stimulation sites were spaced approximately 250 μm apart and 24 ICMS sites were used to map M1$_{FL}$. Movements were visually scored and movement probability maps were generated for forelimb, hindlimb, wrist/digits and trunk.

Motion index and motor pattern discrimination

We defined a region-of-interest (ROI) covering the contralateral (left) forelimb and calculated the motion index (MI) for each successive frame as $\text{MI}_f = \Sigma_{i=1}^{N}(c_{f+1,i} - c_{f,i})^2$, where $c_{f,i}$ is the grayscale level of the pixel i of the ROI in the frame f (see e.g. Figure 1A). We defined grooming as periods with no locomotion and MI$> \theta_m$, where threshold θ_m was determined by visual inspection. Periods shorter than 2 s were not included in the analysis.

The position of the left paw was semi-automatically tracked offline using motion capture software (Blender ver. 2.7.1). In MATLAB horizontal and vertical pixel coordinates of the paw position during each 16.6 ms frame (paw accelerations > 5 pixels.s^{-2}, sustained at > 3 motions.s^{-1}) were re-centered around the average paw rest position for the 1 s preceding the onset of the running bout. To extract repeatable phases of each step during running, movements between local minima in the 1st principal component of the horizontal and vertical motion were classified as individual steps. Horizontal and vertical coordinates between each step onset were then extracted. To quantify similarity of paw movement between individual steps, total path lengths of 2-dimensional paw trajectories and area of enclosed space were calculated for each individual stepping motion.

In vivo electrophysiology and pharmacology

To confirm changes in behavioral state – quiet wakefulness to movement – local field potentials (LFPs) were recorded in M1$_{FL}$ L5B of head-restrained mice. Low-resistance ($\sim$1-2 MΩ) glass micropipettes were filled with external solution and LFP signals were high-pass filtered at 1 Hz.

For pre-application of noradrenergic receptor antagonists (Figure 5), recordings of L5B pyramidal neurons were performed > 40 minutes after drug application. For long-term recordings (whole-cell or cell-attached, Figure 6) the pre-application phase (5-10 mins) involved recording L5B pyramidal neuron activity in the presence of external solution before replacing with a mixture of noradrenergic receptor antagonists. Recordings

continued for >30 minutes to ensure equilibrium antagonist concentration across the entire somatodendritic structure of L5B pyramidal neurons. Due to the time-dependent drift of V_m during long whole-cell recordings (pre: -51.6 mV, after 40 min: -39.7 mV, n=5) the mean membrane potential was corrected by -12 mV in Figure 6B.

Behavioral testing

To assess motor coordination following local noradrenergic receptor blockade in $M1_{FL}$, mice were head-restrained and habituated (20-45 min, over 2-6 days) to walk / run on a cylindrical runged treadmill. Craniotomies were performed above the right $M1_{FL}$ (see Animals and surgery) and after 1.5 h recovery, mice were head-restrained and 2-min video sequences were captured using a digital video camera (25 fps) before and 60 minutes after drug application (1 mM prazosin, yohimbine and propranolol; see *In vivo* electrophysiology and pharmacology in main Experimental Procedures). For sham controls external saline solution was added to the open craniotomy. Left (contralateral) and right (ipsilateral) forepaw placements were scored independently by two researchers and classified as precise forepaw placements or misplacements (i.e. incomplete forepaw placement, single/double digit contact, altered wrist angle, forepaw slips – in front or behind rungs; see Figure 6E).

Drug diffusion kinetics

To estimate the drug diffusion dynamics of topically applied drugs we applied phenol red (10-50 mM, in external solution) to the open craniotomy above $M1_{FL}$ for 60 minutes before slicing the brain in 1 mm sections. Brightfield images were converted to grayscale and pixel intensities were measured along an axis perpendicular to the brain surface. Tissue background pixel intensity was subtracted and pixel intensity was normalized to peak dye concentration at the brain surface.

Retrograde tracing of projection targets

Projection targets of individual neurons were identified by injecting fluorescent retrograde microspheres (RetroBeads, Lumafluor, USA) into the ipsilateral pons and contralateral hemisphere (dorsal striatum, corpus callosum and overlying cortex). Stereotaxic coor- dinates and volumes for pons injections were: 0.4 mm lateral, 3.9 and 4.1 mm posterior to bregma, 0.2 (75 nl), 0.4 (75 nl) and 0.6 mm (50 nl) from the ventral surface of the brain, green beads. For the dorsal striatum: 2.0 mm lateral, 0.2 and 0.6 mm anterior to bregma and 2.7, 2.5 and 2.2 mm ventral to the pial surface, 6x 50 nl, red beads. 2-3 days after injection mice were transcardially perfused with 4 % paraformaldehyde and brains processed to identify injection sites and retrograde staining in cortex.

Reconstruction of neuronal morphology and multi-labeling immunohistochemistry

After each recording, deeply anesthetized mice were transcardially perfused with 4 % paraformaldehyde. Mouse brains were post-fixed overnight and coronal sections (60 or 100 μm) of M1 and tracer injection sites were cut using a vibrating microtome. For morphological reconstructions, sections were incubated in streptavidin AlexaFluor-488

(1:1000, Molecular Probes) in 0.1 M phosphate buffered saline (PBS) containing 0.5 % Triton X-100 then mounted (Vectashield, VectorLabs) and imaged using a Zeiss LSM 510 Meta confocal microscope (20x objective). Morphological reconstructions were generated from 60 μm z-stacks using NeuronJ (ImageJ plugin). To identify projection targets of individually recorded neurons, sections were further processed by heat-mediated antigen retrieval in 10 mM sodium citrate buffer (pH 6.0) for 3 hrs at 80 °C. Sections were incubated in blocking solution (0.01 M PBS, 10 % normal goat serum (vol/vol), 0.5 % Triton X-100 (vol/vol) at 22 °C for 2 hrs and incubated overnight at 22 °C in a primary antibody mixture containing mouse monoclonal anti-SATB2 (1:200, Cat. No. ab51502, Abcam) and rat monoclonal anti-CTIP2 (1:1000, Cat. No. ab18465, Abcam) dissolved in carrier solution (0.01 M PBS, 1 % goat serum, 0.5 % Triton X-100). Slices were then incubated overnight at 22 °C in a secondary antibody mixture containing AlexaFluor-568 goat anti-mouse (1:750, Molecular Probes) and AlexaFluor-647 goat anti-rat (1:750, Molecular Probes) dissolved in carrier solution (0.01 M PBS, 1 % goat serum, 0.5 % Triton X-100), mounted and imaged using a Nikon A1R FLIM confocal microscope (Nikon, Europe). A sequential imaging protocol was used to minimize cross talk between fluorophores. Images were analyzed offline using ImageJ.

To validate the selective expression of CTIP2 and SATB2 in PT and IT neurons respectively, we combined retrograde tracing with post hoc CTIP2/SATB2 immunohistochemistry (see Figure S3 and Figure S4). Quantification of CTIP2 or SATB2 expression in individual, retrogradely identified L5B pyramidal neurons confirmed that all retrogradely labeled PT neurons were CTIP2 immunopositive, with only a small proportion of neurons co-expressing CTIP2 and SATB2 (9.7 %). Similarly, all retrogradely identified IT neurons expressed SATB2, with a minority expressing both molecular markers (2.7 %). For a subset of neurons we employed a dual strategy combining post hoc immunohistochemistry and morphological analysis to confirm the mixed PT- and IT-type projection-class identity of L5B$_{enh}$ and L5B$_{supp}$ subpopulations.

To label noradrenergic fibers in M1, coronal sections (60 μm) were first incubated in blocking solution (0.01 M PBS, 10 % normal goat serum (vol/vol), 0.5 % Triton X-100 (vol/vol) at 22 °C for 2 hrs, then incubated in rabbit polyclonal anti-noradrenaline transporter antibody (anti-NAT; 1:2000, Cat. No. 260003, Synaptic Systems) and then in AlexaFluor-488 goat anti-rabbit antibody (1:750, Molecular Probes). The primary and secondary antibodies were diluted in carrier solution (0.01 M PBS, 1 % goat serum, 0.5 % Triton X-100) and incubated overnight at 22 °C. Slices were mounted and z-stack images were acquired using a Nikon A1R FLIM confocal microscope (40x objective) (Nikon, Europe).

Data analysis

Intrinsic biophysical properties

Spike threshold was defined as the maximum of the second derivative of the voltage trace $V_m(t)$. Spike FWHM is the width of the spike measured at half of the distance between the threshold and the peak. dV/dt peak:trough is the ratio of the peak to the trough of the voltage derivative. Spike accommodation was measured by injecting 60 depolarizing current steps (0.5 s long, 100-500 pA scaled according to input resistance). The accommodation index was defined as $(ISI_2 - ISI_1)/ISI_1$, where ISI_1 and ISI_2 are the first and second inter-spike intervals at the beginning of each current step. Membrane potential sag was measured using hyperpolarizing current steps (250 ms, –100-500 pA scaled according to input resistance) and calculated as the percentage difference between the peak amplitude of the initial response (0-0.1 s) relative to the peak amplitude of the steady state response (0.15-0.25 s).

Membrane potential dynamics

Analysis of the subthreshold V_m dynamics was performed after clipping spikes from threshold to 3 ms after the peak (changing the clipping window to 10 ms before and 20 ms after each spike did not alter the results). The average standard deviation of the voltage (V_m SD) was calculated as the average over non-overlapping periods of 1 s.

We measured the power-spectra of V_m with 75 % overlapping Bartlett 1 s windows. The mean population spectrum was the average of the single cell spectra. The time-frequency spectrograms of the subthreshold voltage were computed with a continuous Morlet wavelet transform.

Detection of compound synaptic events

We could faithfully detect compound EPSPs occurring in a time window (5 ms) shorter than the average membrane time constant (9.0 $\pm$ 2.6 ms) and with a detection threshold of 1 mV. Events that occurred within $\pm$ 10 ms of a spike were discarded. To assess the reliability of our event detection algorithm we simulated noisy membrane potential traces (100 s in duration using Ornstein-Uhlenbeck processes) and manually added events of known amplitude at frequencies ranging from 5-100 Hz. For each frequency range 30 simulations were run and the mean detection efficiency computed (range 90-95 %) across the entire frequency range.

Effective input resistance and membrane time constant

We defined the effective input resistance as $R_{\text{eff}} = 1/G_{\text{tot}}$, the inverse of the total conductance $G_{\text{tot}} = G_L + \langle G_e \rangle + \langle G_i \rangle$, sum of the leak conductance G_L and the average synaptic excitatory and inhibitory conductances $\langle G_e \rangle$ and $\langle G_i \rangle$. For estimating the non-synaptic leak we obtained the typical R_{input} ($\sim$180 MΩ, G_{leak} = 1/R_{input}) from published *in vitro* cortical L5 pyramidal neuron data (Lefort et al., 2009; Sheets et al., 2011). The estimation of excitatory and inhibitory conductances are based on a population mean and the

distribution of R_{input} typically displayed a standard deviation of ~20 MΩ. Since we recorded ~20 cells per group, the error on the estimation of the mean R_{input} is ~ $20/\sqrt{20}$) = 5 MΩ. If we estimate G_e and G_i by varying R_{input} by ± 5 MΩ, the results will differ by < 5 %. Even in the extreme case where R_{input} = 160 MΩ (~20 MΩ change), our estimation of G_e and G_i only varied by ~10-15 %. The effective time constant is $\tau_{\text{eff}} = R_{\text{eff}}C$, where C is the capacitance. R_{eff} and τ_{eff} were estimated from the current injection experiment, calculating the average response to subthreshold injections with more than 20 pulses. We computed the complex impedance $Z(f) = V(f)/I(f)$, where $V(f)$ and $I(f)$ are respectively the Fourier transform of average voltage response and injected current, $|Z(f)| = R_{\text{eff}}/\sqrt{1+(2\pi f\tau_{\text{eff}})^2}$, to estimate R_{eff} and τ_{eff} (Figure S2).

Current injection and spiking probability

To measure the firing probability in response to injected EPSCs, we injected exponentially decaying EPSCs $I(t) = ae^{-t/\tau}$ of variable size a and fixed decay $\tau_s = 5$ ms. EPSCs of different amplitude (in total 80 for each amplitude, range 40-1200 pA) were injected in random order with 100 ± 10 ms intervals. To calculate the relationship between EPSP and EPSC size, we injected a series of 30 EPSCs and measured the average peak EPSP.

To estimate the probability p_i of producing an extra spike, we counted the number of spikes in the 10 ms following and in the 10 ms preceding the EPSC injection. We calculated p_i as the fraction of EPSCs injections of size a_i that produce an extra spike. The probability function $P(w)$ that an EPSP of size w causes a spike is $P(w) = \int_{\theta_r - w}^{\theta_r} p(V)dV$, defining the membrane potential distribution $p(V)$ and the spike threshold θ_r relative to the mean V_m. If we assume a Gaussian distribution for $p(V)$, with zero mean and standard deviation σ, one has $P(w) = \frac{1}{2}[\text{erf}(\theta_r/\sigma\sqrt{2}) - \text{erf}((\theta_r - w)/\sigma\sqrt{2}))]$, where erf is the error function. For each neuron i, we fitted $P_{quiet}(w)$ and $P_{mov}(w)$. For each neuron we calculated the Δ spike probability $S(w) = P_{mov}(w)/P_{quiet}(w)$ and its average between 0-8 mV.

Supplemental References

Lefort, S., Tomm, C., Sarria, J.-C. F. and Petersen, C. C. H. (2009). The excitatory neuronal network of the C2 barrel column in mouse primary somatosensory cortex. Neuron *61*, 301–16.

Sheets, P. L., Suter, B. A., Kiritani, T., Chan, C. S., Surmeier, D. J. and Shepherd, G. M. G. (2011). Corticospinal-specific HCN expression in mouse motor cortex: I(h)-dependent synaptic integration as a candidate microcircuit mechanism involved in motor control. J Neurophysiol *106*, 2216–31.
